# A Cybertaxonomic Revision of the “*Crocidura pergrisea*” Species Complex with a Special Focus on Endemic Rocky Shrews: *Crocidura armenica* and *Crocidura arispa* (Soricidae)

**DOI:** 10.3390/biology13060448

**Published:** 2024-06-18

**Authors:** Leonid L. Voyta, Tatyana V. Petrova, Valentina A. Panitsina, Semyon Yu. Bodrov, Viola Winkler, Lyudmila Yu. Kryuchkova, Natalia I. Abramson

**Affiliations:** 1 Zoological Institute, Russian Academy of Sciences, 199034 Saint Petersburg, Russia; tatyana.petrova@zin.ru (T.V.P.); valentina.panitsina@zin.ru (V.A.P.); semyon.bodrov@zin.ru (S.Y.B.); nataliya.abramson@zin.ru (N.I.A.); 2 Natural History Museum Vienna, 1010 Vienna, Austria; viola.winkler@nhm-wien.ac.at; 3 Research Centre for X-ray Diffraction Studies, Saint Petersburg State University, 199155 Saint Petersburg, Russia

**Keywords:** Crocidurinae, *Crocidura*, endemism, morphospace, *cytb* phylogeny, micro-CT, cybertype, *Crocidura armenica*, endodont, nomenclature, abnormal dentition, morphogenesis, AProMaDesU pipeline

## Abstract

**Simple Summary:**

The genus *Crocidura* has ~200 species, which accounts for roughly half of the Soricidae family’s diversity. The “pergrisea” species group, which comprises at least four species—*Crocidura arispa*, *Crocidura pergrisea*, *Crocidura ramona*, and *Crocidura serezkyensis*—is especially interesting among *Crocidura* endemics of central and western Asian regions. The taxonomic value of a fifth species, *Crocidura armenica*, has been unclear for a long time owing to the poor condition of the skulls of both type specimens. Using a microcomputed-tomography-based cybertaxonomic (CTtax) approach and a newly developed pipeline, “AProMaDesU”, we re-evaluated the type material of the Armenian shrew and expanded the hypodigm of this species using three additional specimens. A morphospace analysis based on three-dimensional craniomandibular datasets revealed the uniqueness of *C. armenica* and *C. arispa*.

**Abstract:**

The extraction of museum DNA from a unique collection of samples of the “*Crocidura pergrisea*” species complex, which comprises local endemics of Central and West Asia, allowed us to determine their inter- and intragroup relationships. The first step of this study was the re-evaluation of heavily damaged type specimens of *C. armenica* via a microcomputed-tomography-based cybertaxonomic approach (CTtax), which enabled a precise description of the species’ morphology; three-dimensional models of the cybertypes were made available through the MorphoBank Repository. We developed the “AProMaDesU” pipeline on the basis of five requirements for micro-CT-based cyber-datasets in relation to mammalian collections. Our second step was a combination of several meticulous approaches to morphological investigation against a background of a *cytb*-based phylogeny, which helped us to make a taxonomic decision about the status of species of the “pergrisea” group, e.g., *C. arispa*, *C. armenica*, and *C. serezkyensis*, when the morphological results were partly incongruent with the molecular phylogeny. Nevertheless, under two assumptions, our findings preserved a separate species-level status of *C. serezkyensis* and *C. arispa*. In addition, we restored the species-level status of *C. armenica*. This taxonomic decision is based on our morphospace analysis, which revealed unique craniomandibular shape transformations within the rocky shrews that helped them with the transition to a new area of morphospace/trophic niches and consequently separated them from the other analyzed *Crocidura* groups.

## 1. Introduction

The family Soricidae (Mammalia: Soricomorpha) is one of the groups of modern mammals with a high level of species diversity, including more than 440 valid species, only slightly behind the rodent families Muridae (834 spp.) and Cricetidae (792 spp.) and the chiropteran family Vespertilionidae (493 spp.) [1]. In the current systematics, shrews, together with hedgehogs, moles, and solenodons, are included in the Laurasiatheria clade and the order Eulipotyphla [2]. Modern soricid species are combined into 26 genera and three subfamilies: Soricinae, Crocidurinae, and Myosoricinae (MSW 2005 [3], HMW 2018 [4]). White-toothed shrews of Crocidurinae are the largest subfamily of living eulipotyphlans [1]; among them, the genus *Crocidura* Wagler, 1832 includes ~200 species, accounting for approximately half of the family’s diversity [1,3]. Almost every year since MSW 2005, zoologists have described several new species of *Crocidura*, mostly from tropical and subtropical regions of Southeast Asia and Africa [5,6,7,8,9,10,11,12,13,14,15], etc.

Despite the success of modern molecular systematics, a number of taxonomic questions remain unresolved, for instance, those concerning some species from Central and West Asia. Among *Crocidura* endemics of these Asian regions, species of the “pergrisea” group are especially interesting [16]. This group of so-called rock shrews (rocky shrews) consists of at least four species: *C. arispa* Spitzenberger, 1971; *C. pergrisea* Miller, 1913; *C. ramona* Ivanitskaya, Shenbrot, and Nevo, 1996; and *C. serezkyensis* Laptev, 1929. For a long time, the composition of the “pergrisea” group has been uncertain because interspecies relations have been resolved exclusively by means of morphological data [17,18] owing to the extreme rarity of collection specimens of each of the aforementioned species.

**Figure 1:**
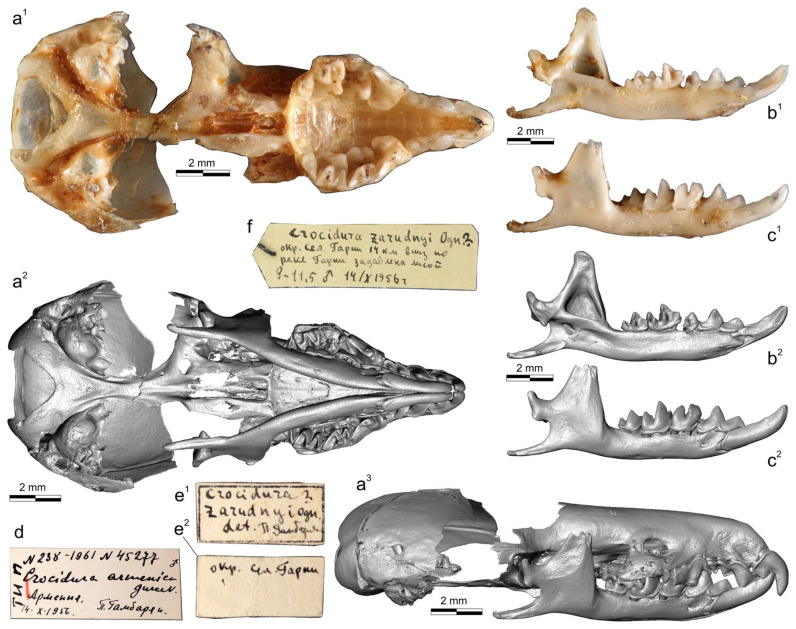
Optic photos (**a^1^**–**c^1^**) and two-dimensional images of three-dimensional reconstructions of skull and separated hemimandibles of the holotype of *Crocidura armenica* Gureev, 1963 (ZIN 45277). (**a^1^**) Skull in ventral view; (**a^2^**) 3D model of the skull in ventral view (digitally repaired in the basisphenoid area, with mandible placed into occlusion); (**a^3^**) 3D model of the skull in lateral view; (**b^1^**) Left hemimandible in medial view; (**b^2^**) 3D model of the left hemimandible; (**c^1^**) Right hemimandible in lateral view; (**c^2^**) 3D model of the right hemimandible; (**d**) Collection label after species description (a red line marks type specimen); (**e^1^**)—Face of original collection label before species description, with text: ‘Crocidura zarudnyi Ogn. det. P. Gambarian’ and (**e^2^**)—Back of e^1^ label, with text: ‘okr. sel. Garni’; (**f**) Original collection label on stuffed skin before species description.

**Figure 2:**
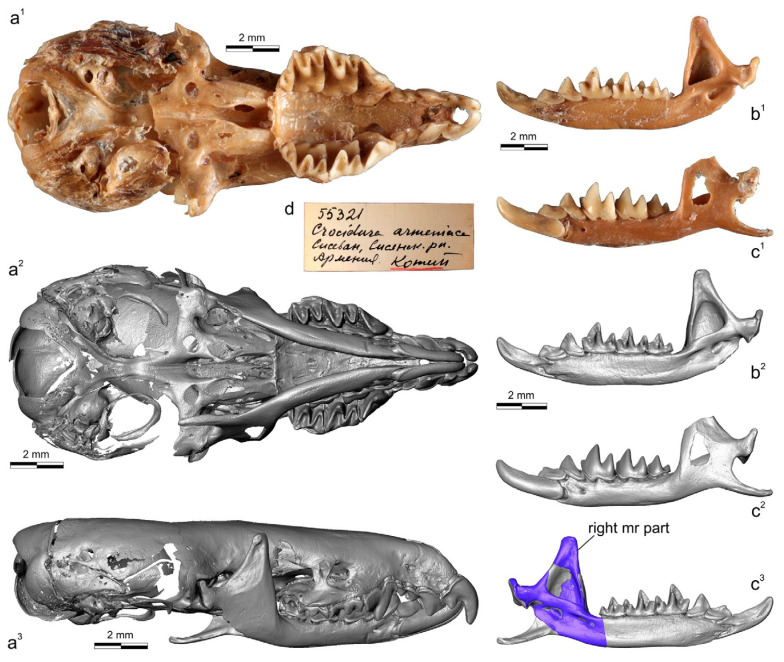
Optic photos (**a^1^**–**c^1^**) and two-dimensional images of three-dimensional reconstructions of skull and separated hemimandibles of the paratype of *Crocidura armenica* Gureev, 1963 (ZIN 55321). (**a^1^**) Skull in ventral view; (**a^2^**) 3D model of the skull in ventral view (digitally repaired in the basisphenoid area, with the mandible placed into occlusion); (**a^3^**) 3D model of the skull in lateral view; (**b^1^**) Right hemimandible in medial view; (**b^2^**) 3D model of the right hemimandible; (**c^1^**) Left hemimandible in lateral view; (**c^2^**) 3D model of the left hemimandible; (**c^3^**) 3D model of combined hemimandibles (base is left part and mandibular ramus is cut from the left part, marked by blue); (**d**) Collection label (a red line marks type specimen).

The successful use of museum DNA extracted from rare collection specimens of *C. arispa* (a local endemic of the Taurus Mts., Turkey), *C. ramona* (a local endemic of the Negev Desert, Israel), and *C. serezkyensis* (a regional endemic of Central Asia) by Bannikova’s research group has allowed researchers to determine the specific position of the “pergrisea” clade among other clades of white-toothed shrews [16]. In this context, a highly topical issue is the taxonomic position of a local endemic of Armenia, *Crocidura armenica* Gureev, 1963, currently represented only by two damaged specimens from the Theriological Collection at the Zoological Institute of the Russian Academy of Sciences (ZIN, St. Petersburg, Russia): a holotype (ZIN 45277) and a paratype (ZIN 55321) (Figure 1 and Figure 2). Because morphological (morphometric) comparisons of the type specimens were limited, the species *C. armenica* was recognized in 2014 as taxonomically invalid [19], and up until now, it has been excluded from the species list of HMW 2018 [4]. Moreover, the ZIN collection includes three *Crocidura* specimens from Nakhichevan Autonomous Republic (Azerbaijan), which is currently listed in open nomenclature as *Crocidura* cf. *pergrisea* (syn. *C. pergrisea* by Zaitsev [17,19]). In the current study, on the basis of external features (e.g., overall fur and tail coloration and ear dimensions) and measurements, we tentatively assigned these specimens to *C. armenica*. In the new context of molecular findings and of the separate position of the “pergrisea” clade [16], the next logical step should be a molecular investigation of the ZIN samples and an attempt to comprehensively synthesize the available data.

For these reasons, there is a need for an integrated approach to these taxonomic issues. Nonetheless, first, we should resolve the following question: what is the “integrated approach” within the taxonomy of the rare species of white-toothed shrews?

### 1.1. Key Provisions of the Integrative Approach

The specific features of white-toothed shrews in comparison to those of soricines are as follows: (i) morphological “plasticity”, which is shown by a high homoplasy of qualitative features [20] and a low level of congruency between morphological and molecular datasets [21]; (ii) the presence of “historical mitochondrial introgression” in some species complexes [14,22]; (iii) a usually low level of abundance in natural habitats and consequently a rarity of specimens in zoological collections; and (iv) access to their natural habitats is usually difficult. Altogether, these features make a comprehensive or “integrative” analysis difficult; such an analysis is usually needed during a taxonomic revision and is based on implementation of several modern approaches, such as molecular and karyological analyses and morphological description, including multivariate morphometric analysis (e.g., Jenkins et al. 2013 [8,11,23]. The current study is a vivid example of the inapplicability of the integrative approach in the traditional sense because rocky shrews are rare; their type specimens are either absent (e.g., for *C. serezkyensis*) or damaged to various degrees (e.g., *C. armenica* and *C. arispa*). In this case, to achieve a sound taxonomic circumscription, we tried to apply a so-called “cybertaxonomic” approach for the first time to mammals.

### 1.2. Cybertaxonomy of Mammals

Perhaps for the first time, the combination of terms “cybertaxonomy”, “cybertype”, and “micro-CT” was used by Faulwetter et al. in 2013 [24,25] for their taxonomic investigation of marine polychaetes, which involved the targeted use of micro-computed tomography (micro-CT) as an essential tool for the modern taxonomist. Nevertheless, in a broader context, applications of information and communications technologies to taxonomy (cybertaxonomy) have been discussed since the beginning of the 2010s [26,27,28].

Faulwetter et al. [24,25] proposed that the main benefit of micro-CT-based cybertaxonomy (CTtax) was the “rapid creation of high-resolution morphological three-dimensional (3D) data, with subsequent interactive manipulation and analysis” ([24], p. 3), with three basic requirements for cyber-datasets: (i) the maximal fit of 3D data (a 3D model) of a cybertype to physical type material “independently of a specific research question“ [24]; (ii) techniques of cybertype creation that are nondamaging toward a physical type specimen; and (iii) the retrievability (i.e., storage of volume CT data) and free accessibility of the cybertype data. On the basis of our experience (L.V., L.K.) with the use of micro-CT in the fields of micromammalian taxonomy and morphological analyses [20,29,30,31,32], we can expand Faulwetter’s basic requirements by two additions that seem important for mammalian type material: (iv) cybertype data should be accompanied with images of a physical type specimen to avoid the incorporation of artefacts into specimen attribution; and (v) cybertype data should be accompanied with full additional information on the scanning procedure (a log file ideally) and object dimensions.

In the current paper, CTtax is used for the first time to describe mammalian type material, at least for shrew type material, in compliance with the five aforementioned requirements. According to the difficulties of the integrative approach that are described above, we assume that there are three main benefits of CTtax as follows: (a) Cybertypes are freely accessible via existing digital repositories (e.g., MorphoBank), and each 3D model of a cybertype and additional specimens (hypodigm) is accompanied by morphological descriptions and two-dimensional images, which will contribute to comprehensibility of the description. (b) 3D datasets of particular morphological complexes (e.g., a hemimandible and isolated tooth) are obtained as raw data for a multivariate analysis, including a morphospace analysis [20,30,32] (regarding this approach, see the recent paper by Polly [33]). The morphospace analysis by means of 3D datasets is a powerful and sensitive tool for describing morphological variety and for comparisons at different levels, and is at least more sensitive than linear-data-based morphometry, which is important for rare and damaged material. Free accessibility of the datasets ensures the reliability and repeatability of results. (c) There is a possibility of “analytical” approaches to the assessment of congruence between molecular and morphological data; this can be helpful in the case of ambiguous results when using some analyses, for instance, for a choice between morphological datasets with different levels of a “phylogenetic signal” [21,34,35]. In the current study, we implemented two of these: (a) and (b).

## 2. Materials and Methods

### 2.1. Sampling

The analysis was performed on two main datasets: 39 specimens of shrews that were micro-CT scanned and represented in the current study through three-dimensional models (Table S1); and 178 specimens used for species size description and represented by two-dimensional images (Table S2). In the study, samples and separated specimens of 16 species were used. The ingroup consisted of specimens of 14 *Crocidura* species: *C. armenica* (holotype ZIN 45277; paratype ZIN 55321; specimens of *Crocidura* cf. *pergrisea*; five 3D models), *C. arispa* (holotype NHMW 13284; one 3D model), *C. caspica* Thomas, 1907 (two 3D models), *C. lasiura* Dobson, 1890 (recent specimens, single fossil specimen KRD; six 3D models, *n* = 18), *C. leucodon* (Hermann, 1780) (two 3D models, *n* = 12), *C. gueldenstaedtii* (Pallas, 1811) (two 3D models, *n* = 30), *C. phanluongi* Jenkins, Abramov, Rozhnov, and Makarova, 2010 (one 3D model, *n* = 5), *C. sapaensis* Jenkins, Abramov, Bannikova, and Rozhnov, 2013 (one 3D model, *n* = 11), *C. serezkyensis* Laptev, 1929 (one 3D model), *C. shantungensis* Miller, 1901 (five 3D models, *n* = 23), *C. sibirica* Dukelsky, 1930 (four 3D models, *n* = 30), *C. suaveolens* (Pallas, 1811) (five 3D models, *n* = 29), *C. zaitsevi* Jenkins, Abramov, Rozhnov, and Makarova, 2007 (one 3D model, *n* = 8), and *C. zarudnyi* Ognev, 1928 (holotype ZIN 6506; one 3D model). Material came from 30 localities: 29 that are recent and one that is from a paleontological site, KorC (Table S3). Outgroups consisted of specimens of *Suncus murinus* (Linnaeus, 1766) (one 3D model) and *Sorex minutissimus* Zimmermann, 1780 (one 3D model) that were chosen for a correct building of the multidimensional morphospace in accordance with our original approach to estimate cranial shape polymorphisms within crocidurine shrews [20].

### 2.2. The Choice of Species

**Figure 3:**
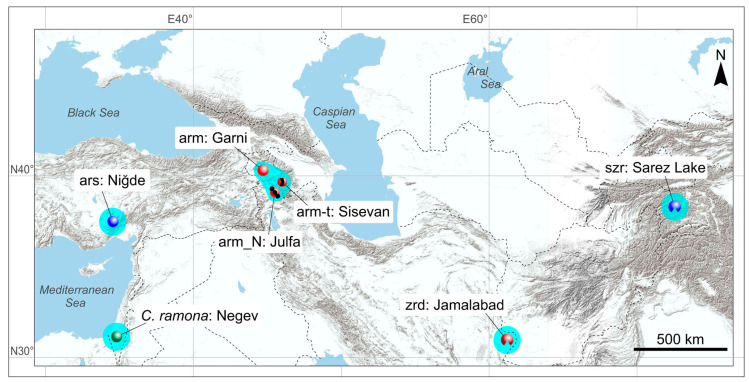
Map of key specimens’ locations. *Abbreviations*: arm, *C. armenica* (holotype, ZIN 45277); arm-t, *C. armenica* (paratype, ZIN 55321); arm_N, a new *C. armenica* hypodigm (ZIN 76972, 973, 976; *n* = 3); ars, *C. arispa* (holotype NHMW 13284); *C. ramona*, type locality of *C. ramona*; szr, *C. serezkyensis* (ZIN 77431; type licality); zrd, *C. zarudnyi* (holotype, ZIN 6506; type locality determined by Nikolay A. Zarudny in his field diary (see Table S3)). Map source: ESRI (https://www.esri.com/; accessed on 1 May 2024) via SASPlanet Application (ver. 160707.9476).

The key sample comprises three species from the “pergrisea” group: *C. armenica*, *C. arispa*, and *C. serezkyensis*, which came from type localities (Figure 3). The main concept of the morphological comparison in the current study is the morphospace estimation approach [33,36]; therefore, there were 13 species added into the analysis for the multidimensional morphospace building. These species were chosen as they had the following characteristics: (i) a geographic position relatively close to the key group within Central and East Asia: *C. suaveolens*, *C. caspica*, *C. gueldenstaedtii*, *C. leucodon*, and *C. zarudnyi* (Table S3); (ii) membership in a certain phylogenetic group [16]: the *C. leucodon* clade (*C. leucodon*) or the *C. suaveolens* clade (*C. gueldenstaedtii*, *C. caspica*, *C. shantungensis*, *C. sibirica*, *C. suaveolens*, and *C. zarudnyi*); (iii) presence in previously studied three-dimensional datasets [20]: the East Palearctic shrew, *C. lasiura*, along with three species from the East Asian clade: *C. phanluongi*, *C. sapaensis*, and *C. zaitsevi*. The chosen species together with two outgroups, *S. murinus* and *S. minutissimus*, allowed us to form a broad morphospace in relation to supposed adaptive morphological trajectories inherent in the analyzed species.

In accordance with a recent molecular study by Bannikova et al. [37], the taxonomic position of *C. zaitsevi* was re-evaluated: the species was recognized as a junior synonym of *Crocidura kegoensis* Lunde, Musser, and Ziegler, 2004. In this case, we needed to use a new species assignment; however, in the current study, we revealed a broad morphometric disparity between type specimens of *C. zaitsevi* (collected in 2004 [5]; Table S2) and single specimen ZIN 96,320 (‘zts’ in Table S1) from the Ngoc Linh type locality (collected in 2006; Table S3). Specimen ZIN 96,320 is a voucher for *cytb* sequence No. HM587003 and belongs to the A lineage of the “kegoensis-zaitsevi” complex [37]; on the other hand, this specimen has distinctly larger cranial dimensions than those of specimens of the type series. In addition, some notable dental features (e.g., a relation between postprotocrista and hypoconal flange elements of M1) are similar between the two morphotypes. This phenomenon requires a special study. Therefore, here we used an open nomenclature for ZIN 96320, *Crocidura* ex gr. *kegoensis-zaitsevi*, and kept a taxonomic name, *Crocidura zaitsevi*, for the type series (Table S2).

### 2.3. Species Determination in 3D Models

In this study, we used 39 3D models of 16 species that cannot exactly cover all states of intraspecies variability, especially for a widespread species (e.g., *C. suaveolens*). On the other hand, it was difficult to include more specimens for two main reasons: the rarity of collection specimens and the time-consuming processes of computation and micro-CT volume segmentation. To avoid both species misidentification of some specimens (3D models) and deviant morphology that can skew the morphospace, we used a set of linear measurements together with relatively representative species samples, which comprised several specimens (including type specimens of *C. phanluongi*, *C. sapaensis*, and *C. zaitsevi*) and 3D model specimens (Table S3).

The nine craniomandibular measurements (Figure S1) were taken according to Lavrenchenko et al. [10], with some changes: “COR” was changed to “MRH” (mandibular ramus height); “PGL” was changed to “EGW” (external entoglenoid width); and in the current paper, “CIL” was measured between the premaxilla and occipital condyle, instead of between the upper incisor and condyle. Therefore, we used the following nine measurements: CIL, condylo-incisive length; EGW, external entoglenoid width; LML, lower molar row length; MBH, dentary height on the m2 level; MRH, mandibular ramus height; P4s/d, the interval between inner margins of the right and left P4; PL, hard palate length; UML, upper molar row length; and ZYG, external width of zygomatic processes of the maxilla. Locally, for the key sample of “pergrisea”, we used two additional measurements: HCD, condylar height; and LLF, lower facet length (Figure S1b).

A univariate comparison between each linear dimension within a sample and a particular value in a 3D model specimen allows for the assessment of the species assignment of the latter against a background of the other external and dental features that were taken by Zaitsev et al. [19] (Tables S4–S11). In such an assessment, in general, all 3D specimens within multispecimen samples (*n* ≥ 7) are located within a particular species’ range of values, except for the sample of *C. zaitsevi* (see above; Table S11). In addition, we used more formal normality tests [38] for more precise assessment of the species attribution on the basis of linear dimensions. Shapiro–Wilk, Anderson–Darling, and Jarque–Bera normality tests were executed using PAST software ver. 2.04 [38]; the results are provided in the Supplementary Materials (Tables S12–S19). According to this approach, we obtain a correct species attribution for each 3D specimen model and conventionally “nondeviant” proportions of their skull and mandible.

### 2.4. Acquisition of Two-Dimensional Images and Measurement Techniques

High-resolution photos of the type specimens and labels were captured with a Canon 60D (Tokyo, Japan) digital camera combined with a Canon EF-S 60 mm f/2.8 Macro USM lens (Tokyo, Japan) and two Godox TT350C (Shenzhen, China) flashes. For acquisition of the linear measurements, 2D images were used that were obtained with the “glass-plate tool” and an Epson Perfection v300 (Suwa, Nagano, Japan) flatbed table scanner (Figures S1 and S2). Each image has a resolution of 2400 dpi and includes a scale bar. Linear measurements were obtained using tpsDig2 software ver. 2.31 [39].

### 2.5. Computed X-ray Micro-Tomography and Segmentation

For scanning of the analyzed skulls and hemimandibles, SkyScan 1172 (CCD) (Kontich, Belgium) and NeoScan N80 (CCD) (Mechelen, Belgium) scanners were used at the Resource Centre for X-ray Diffraction Studies of Saint Petersburg State University (Saint Petersburg, Russia) and a YXLON FF35 CT (XRD 4343CT) scanner at the Natural History Museum of Vienna (Vienna, Austria). Technical characteristics are listed in the Supplementary Materials (Table S1). The specimens were scanned in three-slice mode. Preprocessing was performed with DataViewer software ver. 1.5.4.0 64-bit. The main processing of morphological complexes, such as the muzzle (rostral part of a skull), hemimandibles, and teeth, as well as repair of damaged bones (Figure 1, Figure 2 and Figure S3) were performed using Avizo 2019.1 (FEI SAS). The 3D models together with additional information (see the above CTtax information statements) were deposited in MorphoBank (http://www.morphobank.org; accessed on 1 May 2024; Project 4964). In the current paper, we partly used 3D models of a shrew muzzle that had been published in MorphoBank Project 3885 (http://dx.doi.org/10.7934/P3885; accessed on 1 May 2024).

### 2.6. Morphometric Analysis and Visualization of Results

**Figure 4:**
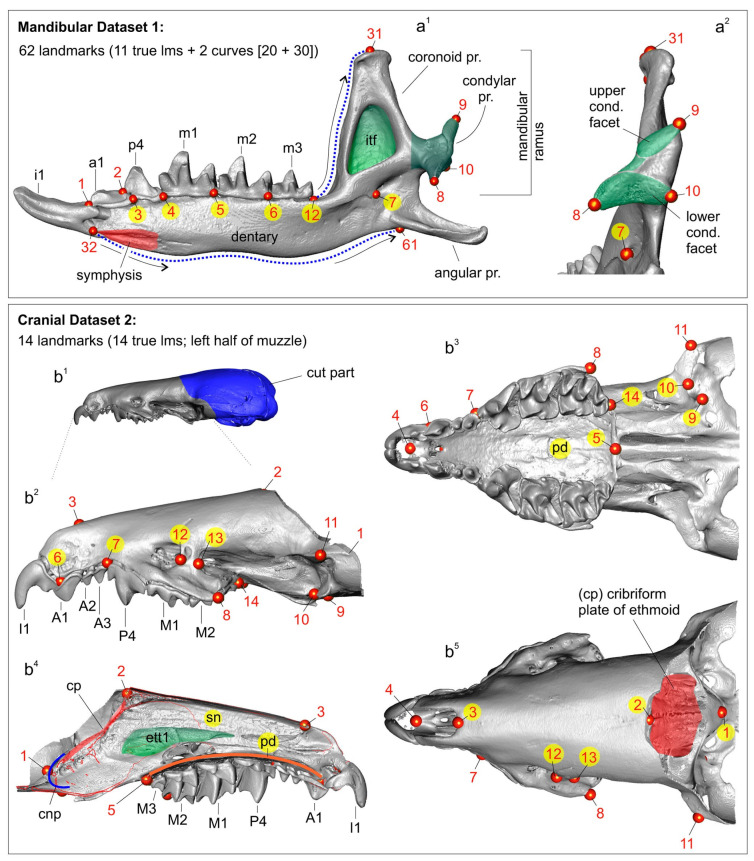
3D landmarks’ and semi-landmarks’ positions on the hemimandible and left half of the muzzle, with labels for the anatomic parts of the bones and teeth. (**a^1^**) Right hemimandible in medial view, with located 11 true landmarks (eleventh lm is hidden) and two curves between 12th and 31st semi-landmarks and 32nd and 61st semi-landmarks; (**a^2^**) Mandibular ramus in posterior view, with locations of 7th–10th and 31st landmarks; (**b^1^**) Diagrammatic image shows a digitally removed part of a skull (dark blue area); (**b^2^**) Left side of the muzzle in lateral view, with locations of 14 true landmarks (4th and 5th are hidden); (**b^3^**) Muzzle in occlusal view, with landmarks; (**b^4^**) Sagittal section of the muzzle shows positions of 1st and 2nd landmarks in relation to the anterior and posterior parts of the cribriform plate (cp); (**b^5^**) Muzzle in dorsal view, with landmarks. Unscaled. Images obtained from the 3D models of *C. suaveolens* (ZIN 73665). Key: An/an, upper/lower antemolars (unicuspid teeth); cond. facet, upper and lower facets of the mandibular condyle; cnp, cupula nasi posterior (by [65]); cp, cribriform plate of the ethmoid; ett1, ethmoturbinal 1; I1/i1, first upper/lower incisor; Mn/mn, upper/lower molars; P4/p4, fourth upper/lower premolar; pd, palatum durum (hard palate); pr., process; sn, septum nasi (nasal septum).

Hemimandible and skull shapes were captured as sets of three-dimensional (3D) coordinates composed of 62 and 14 landmarks, respectively (Figure 4). The main morphometric dataset was the mandibular set owing to the initial assumption of the unsuitability of damaged/distorted skulls of the type specimens of *C. armenica* (Figure 1 and Figure 2) for the 3D analysis. The hemimandible was described by means of 11 true landmarks [40], together with two sets of semi-landmarks, which comprised 20 points (from 12 to 31 lms) along the anterior surface of the coronoid process and 30 points (from 32 to 61 lms) along the lower contour of the dentary (Figure 4; Table S20). The rostral part of the skull—muzzle—was described using 14 true landmarks that almost replicated the landmark frame in a study on crocidurines by Voyta et al. [20], with the following differences: (i) in the current study, we did not use landmarks on the tip of the teeth owing to old age of the type material of the “pergrisea” group, and tooth-based landmarks were replaced by 6th and 7th points on the premaxilla and maxilla bones (Figure S4); and (ii) in this study, we added an 11th landmark for the upper facet of the entoglenoid fossa and a 14th landmark for the M3 alveolus (Figure 4; Table S21). Landmarks and semi-landmarks were processed using 3D Slicer software e ver. 5.4.0 r31938/311cb26 [41]. Stability and repeatability of each landmark’s position and semi-landmark curves in different samples were ensured in the following two ways: (1) making a continuous comparison of the points’ positions with a reference, which was opened in a neighboring screen of given software (two screens of the 3D Slicer); (2) performing three-time landmarking for each specimen, with the final Procrustes analyses using the mean values [32,42]).

The Procrustes superimposition procedure, a principal component analysis (PCA; “relative warp analyses”), and a “live-view” visualization of the shapes’ transformation were performed with the “SlicerMorph” module of the 3D Slicer software [43]. In the final PCA for the description of the results and the preparation of the plots, we used PAST software ver. 4.03 [38]; for this step, Procrustes coordinates were used that were obtained with SlicerMorph.

Pairwise comparisons for the key sample were performed using statistical and graphic environment R. The script was based on the following functions of the “Morpho” [44] and “Rgl” [45] packages, as well as application functions from Claude [46]: *file2mesh*, *read.table*, *centsiz*, *trans1*, *ild2*, *pPsup*, *tps3d*, *mesh3d*: *shade3d*, *plot3d*, and *rgl.snapshot*. This algorithm was tested in crocidurine comparisons in our (L.V., L.K.) earlier papers [20,32].

The mandible and skull shape differences along several loaded PCs were described. The number of loaded (“significant”) components was selected using the “broken-stick model” with 1000 bootstrap replicates by the Jackson algorithm [47] and implemented using the PAST software. The components located above the “broken-stick” line were interpreted in light of the study’s context.

The recent paper of Polly [33] discusses possible misinterpretations of morphospace analysis results. Because our main approach to the interspecies analysis is a morphospace assessment [20,29,30,31,32], we should take into account Polly’s main statement: consequences of ignoring the full dimensionality of morphospace can potentially mislead an investigator [33]. In the present study, to avoid potentially misleading conclusions, we used (a) the Jackson algorithm (see above) for choosing loaded axes that include “biological information” about the shape variation; and (b) cluster analysis (UPGMA) based on Euclidean distances of the Procrustes coordinates. The latter approach allowed us to check our interpretation of morphospace composition (reliability assessment) if the following requirements were met: (b1) cophenetic correlation was not less than 0.7 [38]; and (b2) the cluster analysis supported at least some of the patterns that were revealed in the morphospace.

### 2.7. Statistics

Univariate statistics, plotting of the linear measurement variation, homogeneity tests, and cluster and linear regression analyses were performed using the PAST software [38].

### 2.8. Phylogenetic Analysis

To reconstruct the phylogeny of *Crocidura*, we used 20 *cytb* sequences from the NCBI GenBank, including three fragments of complete mitochondrial genomes that we obtained in a previous study [48] for the specimens from the ZIN Collection (Table 1). *Suncus murinus* served as an outgroup in accordance with Bannikova et al. [17].

Sequences were aligned using the CLUSTALW algorithm [56] implemented in BioEdit [57]. The level of genetic differentiation on the basis of p-distances was estimated in MEGA 7.0.18 [58].

Bayesian inference analysis was carried out in MrBayes 3.2.6 [59]. The dataset was divided into partitions by codon position, and the following parameters were used: nst = mixed; rates = invgamma and partitions as suggested with PartitionFinder results.. Each analysis started with a random tree and had two replicates with four MCMC runs and 1 million generations, with the results recorded every 1000th generation. After evaluation of stationarity and convergence of separate runs using ESS statistics in Tracer v1.7 [60], the consensus tree was constructed based on trees sampled after a 25% burn-in. The final tree was visualized in FigTree v1.6 software (http://tree.bio.ed.ac.uk/software/figtree/, accessed on 26 November 2021).

The nexus alignment file with the nucleotide alignment, mcmc MrBayes run options, and partitioning scheme is available in the GitHub repository via the following link: https://github.com/ZaTaxon/Crocidura-pergrisea-Species-Complex/blob/main/21_FIN_Ban.nex (accessed on 1 May 2024).

The resulting MrBayes phylogenetic reconstruction nexus consensus tree file is also available in the GitHub repository via the following link: https://github.com/ZaTaxon/Crocidura-pergrisea-Species-Complex/blob/main/21_FIN_Ban.nex.con.tre (accessed on 1 May 2024).

### 2.9. Terminology

The dental nomenclature follows that of Reumer [61], Dannelid [62], and Lopatin [63]. Cranial terms follow those of Wible [64] and Maier et al. [65]. The endodontic nomenclature of the upper teeth follows that of Voyta et al. [66]; the endodontic nomenclature of the lower teeth is provided here for the first time. In this study, we use the term “hypodigm” in Simpson’s sense [67] for the listing of all specimens that we assigned to particular species; i.e., in the current paper, we attempt to expand the hypodigm of *C. armenica*.

### 2.10. Institutional Abbreviations

FSC is the Federal Scientific Center of the East Asia Terrestrial Biodiversity, the Far Eastern Branch of the Russian Academy of Sciences, Vladivostok, Russia; IZEA is Institut de Zoologie et d’Ecologie Animale, Lausanne, Switzerland; NHMW is Natural History Museum Vienna, Vienna, Austria; TAU is Tel-Aviv University; ZIN is Zoological Institute of the Russian Academy of Sciences, St. Petersburg, Russia; and ZMMU is Zoological Museum of the Moscow State University, Moscow, Russia.

## 3. Results

### 3.1. Description of Cybertypes

**Figure 5:**
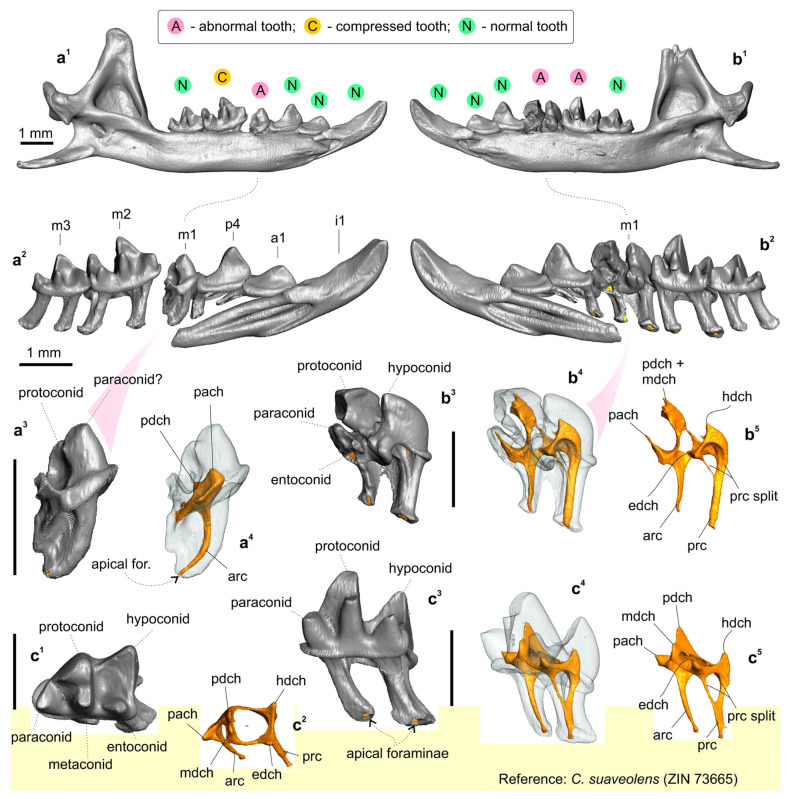
The mandible and lower teeth of the *Crocidura armenica* holotype, ZIN 45277; and first lower molar of the reference specimen of *C. suaveolens*, ZIN 73665. (**a^1^**) Left hemimandible in medial view; (**a^2^**,**b^2^**) Separated 3D models of lower row of teeth in medial view; (**a^3^**) Left aberrant m1 in medial view; (**a^4^**) ibid., in transparent medial view; (**b^1^**) Right hemimandible in medial view; (**b^3^**) Right m1 in posteromedial view (coronoid process is damaged); (**b^4^**,**c^4^**) *ibid*., in posteromedial transparent view; (**b^5^**,**c^5^**) Pulpal endocast of m1 in posteromedial view; (**c^1^**) Right m1 of *C. suaveolens* in occlusal view; (**c^2^**) Pulpal endocast of m1 in occlusal view; (**c^3^**) Right m1. in medial view. All images are screenshots of 3D models. Scale bars are 1 mm. Key: arc, anterior radicular canal; edch, entoconid pulpal chamber; hdch, hypoconid pulpal chamber; mdch, metaconid pulpal chamber; pach, paraconid pulpal chamber; pdch, protoconid pulpal chamber; prc, posterior radicular canal; prs split, split the base of the posterior radicular canal, which is a stable feature of the lower molars (see also Figure 4).

A holotype of *C. armenica* (ZIN 45277) is housed in the Theriological Collection of [68] and comprises stuffed dry skin, a skull, and two separated hemimandibles. The skull and mandible are heavily damaged. The skull is broken: a rostral part is separated from the braincase, and large fragments of the frontal, parietal, and squamosal bones are lost, as are both ectotympanic rings. Auditory ossicles are partly present, including fragments of the malleus, incus, and right stapes (Figure S5). Both halves of the skull were put together with glue in a basisphenoid–basioccipital junction area, with visible shifting (Figure S6). The left hemimandible is whole, and the right hemimandible has a broken tip on the coronoid process (Figure 5).

The dentition contains relatively normal and abnormal teeth. The upper row of teeth shows a normally developed first incisor, three antemolars that follow this, and a third molar on both dentition quadrants (Figure S8). The fourth premolars have a deep groove in the anterior base of the main cusp and abnormal wear facets due to deviant occlusion with the lower teeth; the left P4 even has an opened pulpal chamber of the main cusp. The left first upper molar has developed as two separate parts, the buccal one with an abnormally shaped paracone and metacone and the lingual part with an abnormally shaped protocone and a relatively normal hypoconal flange (Figure S8). The right M1 is a whole tooth with a more or less normal development of the hypoconal flange, an abnormal cone-like protocone, and the buccal tooth portion with a heavily changed shape. The posterior emargination is undeveloped; the edge is undulated and close to M2 (Figure S8). The left M2 has a normal shape at least to the naked eye, except for seemingly deeper posterior emargination, as compared to that of a normal reference tooth (e.g., a *C. armenica* paratype; see below). The right M2 is abnormal, with an altered development and corresponding shapes of all main cones, cristae, and styles. The hypoconal flange has a seemingly normal shape, but the hypoconal shape on the right slightly differs from that of the hypocone on the left (Figure S8). Both third upper molars have a normal shape and development of crown elements; the teeth are three-rooted.

Similarly to the upper dentition, the lower row of teeth consists of normal and abnormal teeth, which, however, seem atypically compressed in the mesiodistal direction; the first lower incisor and m1 of the left hemimandible are visibly tilted/crowded in comparison to the reference row of *C. suaveolens* (Figure S9(a^2^ cf. d)). The left hemimandible is composed of a relatively normally developed first incisor, first antemolar, fourth premolar, and third molar. The first molar has an atypical antemolar-like unicuspid morphology. The second molar is compressed and tilted in the alveoli with a broad gap off the m1 talonid (Figure 5(a^1^–a^3^)). A pulpal endocast of the left m1 showed a well-developed anterior radicular canal that corresponds to the condition and topology of a normal m1 (Figure 5(a^4^ cf. c^5^)). The single pulpal chamber has an irregular shape with several extensions. The more massive anterior extension was attributed by us to the paraconid pulpal chamber (pulpal “horn”: *pach*). The second small extension was determined by us to be a weakly developed protoconid pulpal chamber (*pdch*). The enamel cover of the crown is unequally thick and punctured by several holes of various sizes (0.03–0.05 mm). The right hemimandible bears relatively normal anterior, i1–p4, and last molar teeth. The main chewing teeth, m1 and m2, have deviant features. Unlike the left m1, the right m1 is oversized, with a massive proto- and hypoconid. The paraconid is separated from the protoconid base by a deep and narrow incisura; at first glance, the paraconid seems to be a separate part (Figure 5(b^2^)). The metaconid is hidden in the inflated mass of the protoconid, the entoconid area is represented by a double extension, and the conid can be identified only conventionally. A pulpal endocast of the right m1 helped to determine the pulpal relation between the paraconid chamber and combined chambers of the proto- and metaconid (Figure 5(b^4^,b^5^)). In comparison to the reference pulpal endocast of *C. suaveolens*, we can see a notable atypical overhanging of the buccal conid pulpal chambers; the chambers of the proto- and hypoconid (*pdch* and *hdch*) are above the lingual part. On the other hand, the radicular part of the tooth is seemingly regular, including a typical feature of the posterior radicular canal: a split in the canal (*prc split*; Figure 5(b^5^ cf. c^5^)). The second molar has a heavily compressed trigonid with an atypically narrow basin and a defect of the metaconid base: a transversal groove/ledge (Figure S10). The main comparisons between the right p4/m1 row and the reference teeth of *C. suaveolens* are presented in the Supplementary Materials (Figure S11). The enamel layer of m1 is also irregular in terms of its thickness, with small holes.

A paratype of *C. armenica* (ZIN 55321) is housed in the Theriological Collection of ZIN [68] and comprises stuffed dry skin, a skull, and two separated hemimandibles. The cranium was extracted from a shrew body preserved in liquid. The skull bones’ condition (friable bones), coloration (pale to deep brown), and dense associated soft tissues (Figure 2(a^1^,b^1^,c^1^)) are suggestive of formaldehyde in the preservation liquid. Probably this chemical prevented successful DNA extraction despite the preserved mass of a soft tissue on the skull (see below). In addition to the bad liquid fixative, the skull was found to be damaged mechanically: the nasal bones are broken in the anterior part, the left part of the braincase has a broad indentation with a lost squama of the parietal and temporal segments, and the supraoccipital segment is present as two unassociated parts. The auditory ossicles and ectotympanic rings are partly retained. The left ring is almost fully retained, while the right ring is broken. Fragments of mallei are present, and other ossicles were lost (Figure S12). The defects of different parts of the skull are shown in the Supplementary Materials (Figures S13–S15). It should be noted that the formaldehyde fixative substantially decreased the tissue thickness before the transfer of the body to a dry collection; therefore, we can see many micro-CT artefacts (Figures S13–S15). The left hemimandible has a broken tip on the coronoid process and a wide hole in the wall of the internal temporal fossa (Figure 2 and Figure S16). The right hemimandible has a broken part on the angular process base; this problem restricted our acquisition of dimensions (Figure 2). For the purpose of geometric morphometric analyses of the mandibular dataset, we repaired the hemimandible shape by combining the left hemimandible with a mandibular ramus of the right hemimandible (Figure 2 and Figure S3).

The dentition consists of teeth with a normal morphology. In addition, the teeth have mild wear due to the immaturity of the animal and can be correctly compared with other specimens. I1 with a clear-cut ectocingulum; the talon is small in relation to that of A1. The root part of I1 is only half of its crown part’s length (according to radiographic data; Figure S15(a^2^)). A1 is the largest upper antemolar tooth. A2 is notably larger than A3. The antemolars have obvious cingula (ecto- and ento-) and a diagonally directed posterocrista (see Ch. 12, State B in [20]). The posterocrista reaches a posterobuccal corner of the antemolar crown. A3 bears a posterior margin notch (see Ch. 18, State B in [20]; Figure S15(a^3^)). P4 shows deep posterior emargination and a well-developed hypoconal flange. The parastyle as a distinct tip moderately protrudes forward. The paracone is high, and the centrocrista is straight with a weak bend at the point of contact with the paracone tip. The protocone is small; the preparaconal crest (preprotocrista) is fused with a sharp anterocingulum. The hypocone as a cone-like cusp is absent; the hypoconal flange margin forms a low narrow endocingulum portion (Figure S15(a^3^)). The first and second upper molars have well-developed main cones. The mesostyle is represented by a sharp clear oblique crest without a split. The protocone is well developed with a relatively long straight postparaconal crest (postprotocrista). The hypocons of M1 and M2 are very weak and are present in the anterior part of the entocingular crest, which rounds the hypoconal flange (Figure S15). M3 is clearly four-rooted.

The lower dentition for the first time is described here through the crown elements’ morphology in combination with the endodontic elements. All of the teeth (i1–m3) have a developed, narrow, and clearly distinguished ectocingulid. The first lower incisor has a slightly undulated cutting edge, a well-developed entocingulid lobe, and deep lateral and medial root grooves. A pulpal endocast revealed a long incisor pulpal horn (*iph*) that actually determined the crown part of the mesial portion of the tooth. The exaenodonty [69,70] of the buccal (distal) portion of the first lower incisor is related to a posterior expansion of the crown that is marked (in the pulp) by an ectocingulid commissura (*ecc*; Figure S16). Two separate superior and inferior radicular canals (*src* and *irc*) are fused in the last third of the root into a common canal. The first lower antemolar has a long and low crown and a short root. A pulpal endocast consists of a single chamber (*a1ch*) and three canals within the crown part: two supporting the ecto- and entocingulum; and a central, slightly arched canal supporting a postcristid. The fourth lower premolar has a high crown with a sharp main cusp. We can see two well-distinguished arms of the postcristid: the buccal arm disappears before reaching the posterobuccal corner of a crown; the lingual arm is more notably pronounced from the tip to the posterolingual corner. Furthermore, in the pulpal endocast, we can see a diagonal canal that supports the lingual arm along its entire length, whereas for the buccal arm, we see small remains of the similar buccal canal. The main cusp of p4 is supported by a pulpal chamber (p4ch). The root part of p4 is composed of two canals: a small anterior radicular canal (*arc*) and a posterior, main, radicular canal (*mrc*). Both correspond to anterior and posterior roots. The first and second molars gradually decrease in size but have similar crown and pulpal features (Figure S16e). The trigonid is high, with well-developed notches of the para- and protolophid, namely a carnassial and “protocristid” notch [63], respectively. The hypoconid is high, the postcristid is gradually concave along the entire length, and the hypoconulid is undeveloped. The oblique cristid reaches the protoconid base on a relatively high level; hence, the hypoflexid has a small area and is located far from the ectocingulid (Figure S16(c^3^)). The entoconid is large, and the entocristid is undeveloped. The entocingulids of m1–m2 are developed just along the trigonid base. The pulpal endocasts of m1–m2 show a continual anterior part with chambers of the protoconid, metaconid, and paraconid. The talonid part consists of hypoconid and entoconid chambers that are linked by a well-developed canal supporting the postcristid. Two canals of the talonid support ecto- and entocingulids. The root part consists of two roots. The anterior root bears a wide radicular canal (arc). In the talonid part, two separated radicular canals, which seemingly correspond to ancestral hypo- and entoconid independent roots, are fused into a common posterior canal. The feature of unfused canals is herein called a “split” of the posterior canal (*prc split*). The split is undeveloped in the m3 root part. The third molar has a developed trigonid and a short talonid with a low hypoconid. In the pulpal endocast, we can see a gap in the buccal canal; hence, the hypoconid chamber seems to be an entoconid chamber (Figure S16(d^3^)). Nonetheless, the single talonid chamber should be attributed to the hypoconid.

### 3.2. Phylogenetic Analysis

The complicated issues of determining the taxonomic status of *C. armenica* using morphological data alone are illustrated by the detailed morphological description given above. For instance, Zaitsev [17] used a multivariate analysis of the “pergrisea” group, including the *C. armenica* holotype (with abnormal dentition and compressed rows of teeth), *C. serezkyensis*, and *C. pergrisea* (specimens from Julfa, Azerbaijan). Currently, many positive examples of taxonomic and phylogenetic problems solved using museum DNA from museum samples are known, e.g., [16,71,72,73]. In a similar way, we obtained DNA from three out of the four specimens, i.e., from *C. armenica* (ZIN 45277, 55321), *C.* cf. *pergrisea* (ZIN 77972), and *C. serezkyensis* (ZIN 77431), except for the paratype of *C. armenica* (ZIN 55321), which had been kept in the formaldehyde fixative for a long time.

**Figure 6:**
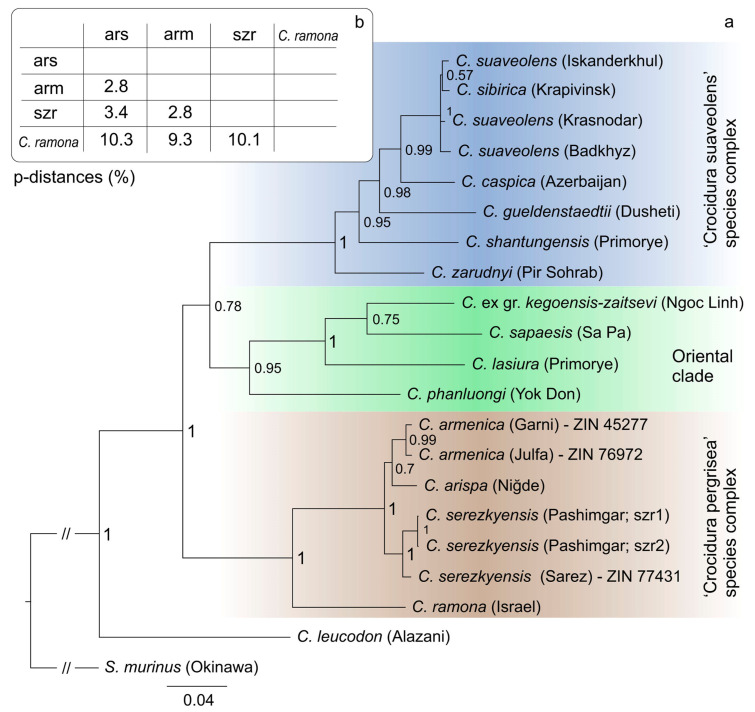
Bayesian phylogeny of *Crocidura* as deduced from the *cytb* gene (**a**) and genetic distances between species of rocky shrews (**b**). Values near the nodes correspond to Bayesian posterior probabilities (bpp). Sequences added in the current study marked by the collection numbers, ZIN (Table 1). *Suncus murinus* serve as outgroup. ‘Oriental clade’ consists species from the eponymous clade by Bannikova et al. [37]; “species complexes” are according to Bannikova et al. [16]. Key: arm, *C. armenica*; ars, *C. arispa*; szr, *C. serezkyensis*.

The Bayesian *cytb* tree revealed three phylogenetic clades such as the following: (1) the “oriental” clade [16], which in the current study is represented by the Northeast Palearctic shrew, *C. lasiura*, and three Asian tropical shrews, *C.* ex gr. *kegoensis-zaitsevi*, *C. sapaensis*, and *C. phanluongi*; (2) the “*C. suaveolens* group” clade sister to the first one, combining six species including *C. gueldenstaedtii* and *C. zarudnyi*; and (3) the “*C. pergrisea* group” clade, which combines four known species of rocky shrews: *C. armenica*, *C. arispa*, *C. serezkyensis*, and *C. ramona* (Figure 6a) and is a basal clade. Intraclade relationships and composition are similar to those revealed by Bannikova et al. [16], except for the position of new original sequences of *C. armenica* and *C. serezkyensis* from type localities (Garni, Armenia; and Sarez Lake, Tajikistan) and *C.* cf. *pergrisea* from Julfa (Table S3). The main topology of the “pergrisea” clade is stable even with the addition of new datasets. The sequence of *C. serezkyensis* from the type locality is close to sequences from the Pashimgar locality, and the sequence of *C. ramona* is the most distant from the other species within the group. On the basis of a comparison of external features (fur and tail coloration), it was expected that sequences of the *C. armenica* holotype and of the shrew from Julfa (*C*. cf. *pergrisea*) are combined into a single clade. Nonetheless, the analysis revealed an intriguing relationship between the sequences of *C. armenica* and *C. arispa*. The genetic distance between *C. serezkyensis* and *C. arispa* (p-distance of 3.4%) and between *C. serezkyensis* + *C. arispa* and *C. ramona* (ca. 10%) have stayed at approximately the same levels that were revealed by Bannikova et al. [16], whereas the addition of the *C. armenica* sequences showed a minimal genetic distance of 2.8% between *C. armenica* and *C. arispa* in the “pergrisea” group (Figure 6b).

From the results of the phylogenetic analysis, we can conclude that the *C. armenica* holotype (*cytb* sequence OR449074) and the specimen of *C*. cf. *pergrisea* from Julfa are conspecific. Their conspecificity enables us to expand the species hypodigm of the Armenian shrew by three specimens from Julfa: ZIN 77,972 (*cytb* sequence OR449075) + ZIN 77,973 and ZIN 77976. In turn, the mutual conspecificity of the Julfa specimens is supported by a karyological study by Grafodatsky et al. [74]. We can also accept the conspecificity of the new hypodigm of *C. armenica* (the holotype and three new specimens) with the paratype of *C. armenica* (ZIN 55321), which could be not included in the molecular analysis (see above). This attribution is mostly conventional. It is based on the similarity of external features between the holotype and paratype and is the weakest point of our argumentation. Therefore, in the following analyses, we will test the conspecificity by means of phenotypic data.

### 3.3. Species Comparisons

The next step of the investigation into rocky shrews was interspecies comparisons for (a) determining each species-specific set of traits and (b) finding phenotypic relations between type specimens of *C. armenica* and the newly found Armenian shrews from Julfa.

If one wants to use external features, such as fur coloration and standard external dimensions, then it should be noted that not all of these are available for study; e.g., there is no information about dimensions in the collector datasheets (labels) of the holotype and paratype of *C. armenica*. We suppose that the external measurements in the first description of the *C. armenica* holotype were made by Gureev on dry stuffed skin (Table 2; [75]). Nevertheless, the key sample possesses several notable features that distinguish species. The overall coloration of the back of rocky shrews is lighter than that of other sympatric shrews, such as *C. gueldenstaedtii* and especially *C. leucodon* (see Plates XIII/1–2 in Kryštufek and Vohralík, ref. [18]). By contrast, *C. arispa* has more brown shades in its dorsal fur and a darker shade of its belly than *C. armenica* (Figure S17). Armenian shrews as type specimens, similar to the new hypodigm specimens, have a tail size similar to that of *C. gueldenstaedtii* (the values of the Armenian shrew lie within the *C. gueldenstaedtii*’s range of characteristics), *C. arispa*, and *C. serezkyensis* and slightly longer than that of *C. leucodon* (Table 2). Nevertheless, *C. armenica* differs from the other compared species in its tail coloration. It has a visibly dark tip only, whereas *C. arispa* has a darker tail (Figure S17). *C. serezkyensis* has a fully pale tail (Figure S18). *C. arispa* differs from the other species in terms of ear size: 10.1 mm in *C. arispa* [76], 9.2–9.4 mm in *C. armenica* (*n* = 2), and 7.0 mm in *C. serezkyensis* (*n* = 1).

A comparison of the analyzed species in terms of craniomandibular and dental linear measurements revealed that sympatric species, such as *C. leucodon*, *C. gueldenstaedtii*, *C. suaveolens*, and *C. zarudnyi*, overlap in most characteristics with species of the “pergrisea” group (Table 2; Figure S19). The damaged skull with an abnormal dentition of the *C. armenica* holotype (ZIN 45277) has significantly smaller values of molar row length (LML and UML) in comparison to those of other specimens of the Armenian shrew. Thus, in contrast to Zaitsev’s conclusion [17], we believe in the unsuitability of the holotype’s linear characteristics for the interspecies comparisons that follow. The measurements of the paratype of *C. armenica* (ZIN 55321) moderately differ from the values of the Julfa specimens of *C. armenica*, e.g., in terms of ZYG or external entoglenoid width (EGW) values, which showed homogeneity in most of the analyzed samples (Table 3). These differences can be explained by the geographic distance between the samples and confirm the absence of an effect of the cranium damage on the linear values. Of note, the combined hemimandible (Figure 2(c^3^)) has the correct mandibular ramus height (MRH) value within the range values of the Julfa sample (MRH = 4.33 mm and 4.29–4.33 mm, respectively). Several dimensions showed specific differences between species: (i) the mandibular body height (MBH) of *C. zarudnyi* is notably different from that of the other analyzed species (MBH = 1.49 mm vs. range of 1.12–1.34 mm by mean values); *C. serezkyensis* has a minimal MBH; (ii) the MRH of *C. arispa* showed a notable difference from that of the other analyzed species (MRH = 3.88 mm vs. 3.95–4.72 mm by mean); *C. leucodon* has a maximal MRH (4.72 mm by mean), which, even at the limits of this range, does not overlap with that of the rocky shrew; (iii) *C. zarudnyi* showed a maximal LML (4.70 mm by mean) among the compared species; other species, except *C. leucodon* (4.06 mm by mean), have approximately similar ranges of LML (3.78–3.93 mm, by mean); and (iv) in contrast to LML, the UML of *C. zarudnyi* lies within the range of the compared species (UML = 3.34 mm vs. 3.04–3.42 mm by mean). Accordingly, in Table 2, our analysis reveals the similarity of the compared species in terms of their linear characteristics, except for *C. leucodon* and *C. zarudnyi*, which showed some clear differences, e.g., the former had the largest MRH (former) and the latter had the largest LML (latter). We also revealed distinctive values in *C. arispa* (the minimal MRH) and *C. serezkyensis* (the minimal MBH), which, however, require representative datasets for confirmation.

Because the mandibular condylar process is considered one of the most important morphological complexes that supposedly bears a morphofunctional signal and has been traditionally used in the species diagnostics of shrews, we determined two additional linear characteristics of the condyle (Table 4): the overall condylar height (HCD) and lower condylar facet length (LLF). These results uncovered obvious differences between the small-condyle group and large-condyle group. The former group is composed of all rocky shrews together with *C. suaveolens* and shows an HCD range of 1.07–1.38 mm and an LLF range of 1.00–1.24 mm. The latter group of *C. gueldenstaedtii*, *C. leucodon*, and *C. zarudnyi* clearly differs from the former by an HCD range of 1.42–1.64 mm and more weakly by an LLF range of 1.18–1.48 mm (Table 4). Among rocky shrews, the smallest size of the condyle belongs to *C. serezkyensis* (HCD = 1.07, LLF = 1.00 mm). The traits of *C. armenica* are different from those of *C. serezkyensis* in terms of its greater HCD (1.20–1.38 mm) and greater LLF (1.13–1.19 mm). Interestingly, the condyle size of *C. arispa* is similar to that of the paratype of *C. armenica* (ZIN 55321) in terms of HCD and to that of the specimen from Julfa (ZIN 77976) in terms of LLF.

**Figure 7:**
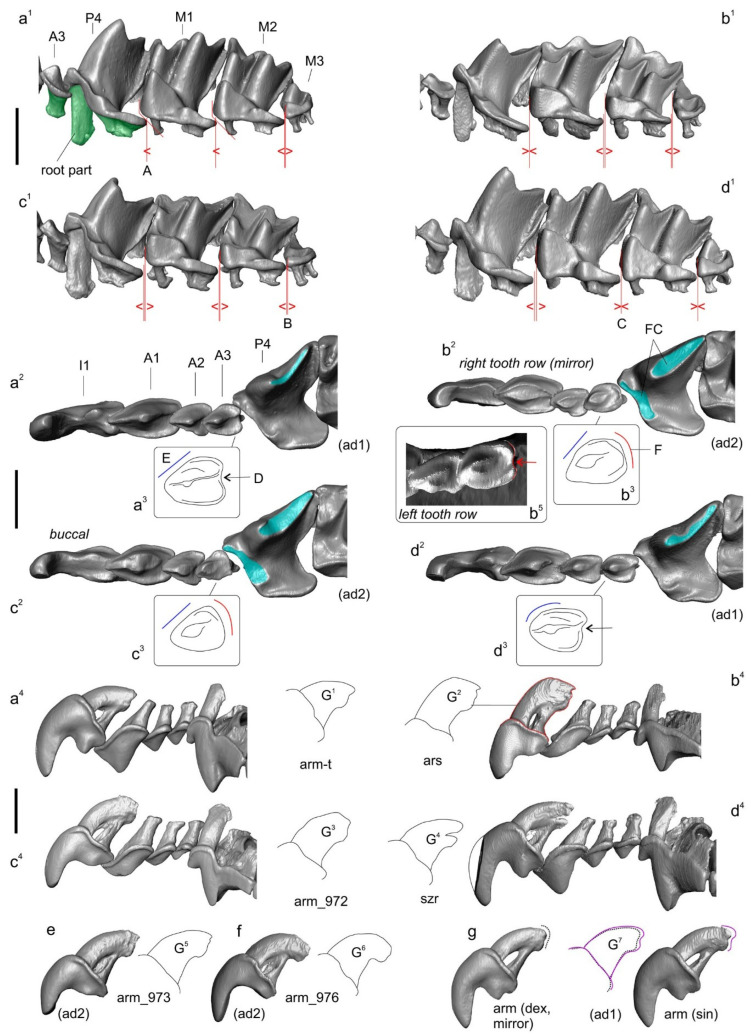
Dental characteristics of *C. armenica* (paratype, ZIN 55321—(**a^1^**–**a^4^**); ZIN 76972—(**c^1^**–**c^4^**); ZIN 76973—(**e**); ZIN 76976—(**f**); holotype, ZIN 45277—(**g**)), *C. arispa* (holotype, NHMW 13284—(**b^1^**–**b^5^**)), *C. serezkyensis* (ZIN 77431—(**d^1^**–**d^4^**)). (**a^1^**–**d^1^**) Upper teeth A3–M3 in lingual view; (**a^2^**–**d^2^**) Upper teeth I1–P4 in occlusal view; (**a^3^**–**d^3^**) Explanatory drawings of A3 crown outline; (**a^4^**–**d^4^**) Upper teeth I1–P4 in labial view; (**b^5^**) Fragment of the right upper row of teeth is shown an alternative characteristic state of the A3 posterior margin; (**e**,**f**) Isolated I1 in labial view; (**g**) *ibid*., right (mirror. view) and left teeth of holotype. Key: A, dental crowding in P4–M1 position (for alternative states, see (**b^1^**) (‘><’—in line) and (**c^1^**) [‘<>’—gap]); ad, adult animal (different stages—ad1 and ad2, by the wearing facet conditions); B, gap (sparse teeth) in M2–M3 position; C, crowding in M1–M2 position; D, a posterior margin notch of A3 crown (expressed on the right A3 of *C. arispa*—(**b^5^**) and *C. serezkyensis*—(**d^3^**)); E, shape of the buccal side of A3 (two states (‘straight margin’—(**a^3^**–**c^3^**)) and (‘rounded margin’—(**d^3^**))); F, a posterior margin notch is absent (**b^3^**,**c^3^**); FC, wearing facets on the P4 crown surface corresponding to the relative age stages: sad–ad2; (**G^1^**–**G^6^**), the I1 root contours; (**G^7^**), fitted root contours of the *C. armenica* holotype; sad, subadult animal; the symbols <> and >< are used to indicate the increasing and decreasing of an intertooth gap; see also Table S1. Scale bars are 1 mm.

Our examination of the qualitative characteristics of the rocky shrews revealed their overall similarity primarily in odontological characteristics because their other phenotypic traits (e.g., craniomandibular) are either indistinguishable to the naked eye or highly variable. The latter aspect is very difficult to assess in rare samples such as ours. Moreover, key specimens, such as the *C. arispa* holotype and the new hypodigm of *C. armenica*, have heavily worn teeth. Nevertheless, we detected a similarity in the upper molars’ outline, in the shape of P4 parastyles, in the position and shape of protocons and hypocons of P4, and in the M1–M2 upper molars. On the other hand, we found different degrees of sparsity of their upper molariform teeth (Figure 7). This feature, at first glance, clearly differentiates the crowded teeth of the *C. armenica* paratype from the sparse teeth of the *C. arispa* holotype. Nonetheless, dental crowding seemingly varied among individuals, as one can see in the sample of *C. armenica* specimens and in the other species’ samples to various degrees.

The next feature, the shape of the posterior margin of A3, was examined here mostly according to Zaitsev [17] and Kryštufek and Vohralĺk [18]. We found an overlap of A3 crown features between the rocky shrews, namely: (i) Zaitsev [17] described a notch of the posterior margin of A3 as an inherent feature of *C. serezkyensis*, and this feature was used by Kryštufek and Vohralĺk [18] for the differentiation of *C. arispa* (notch absent) and *C. serezkyensis* (notch present). In contrast, the notch was present in *C. armenica* (it varied) and, contrary to the colleagues’ statement [18], we found a well-developed notch on the left A3 of *C. arispa*, whereas on the right A3, it was absent (Figure 7(b^3^,b^5^)); and (ii) The buccal contour shape of A3 seems to distinguish *C. serezkyensis* from *C. arispa* and *C. armenica*.

Initially, during the description of cybertypes, we revealed a new specific feature: the relative root size of the first upper incisor (Figure S15). The comparison between the roots of *C. armenica* (paratype) and *C. serezkyensis* revealed a notable difference between these species. A precise comparison of incisors only within the rocky shrew specimens uncovered a variation in root size. For example, within our sample of *C. armenica* individuals, the root size can vary from a small-sized morphotype (paratype state; Figure 7: state G^1^) to a large-sized morphotype (state of ZIN 77,976 specimen; Figure 7: state G^6^). The root contours of the holotype and paratype are similar, with a small difference in size. Nonetheless, the root of *C. arispa* is the largest anyway and has another inclination between the crown base (by the ectocingulum line) and the root longitudinal axis.

Our visual comparisons of the rocky shrews’ skulls revealed weak differentiation in terms of their dorsal contours: *C. arispa* has a small downward bend of the contour, and *C. armenica* and *C. serezkyensis* have relatively straight contours (Figure S20).

### 3.4. Geometric Morphometric Analysis

The main approach to interspecies comparisons in the current study—vis-à-vis the high level of introgression of linear characteristics—is morphospace estimations on the basis of 3D datasets and a principal component analysis (PCA) as a dimension reduction technique.

**Figure 8:**
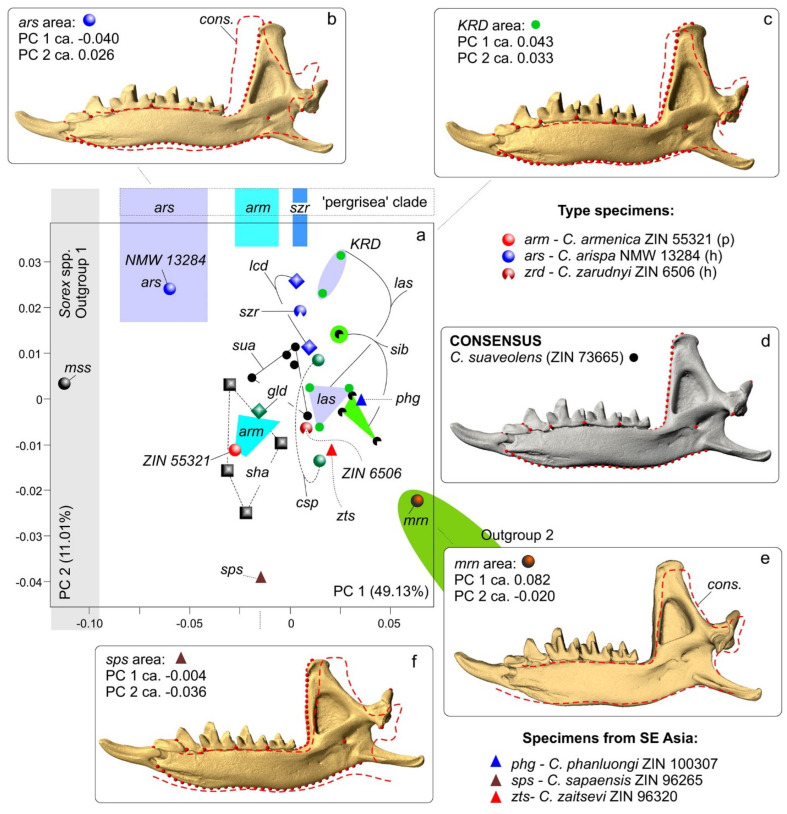
Results of the mandibular shape analysis of the 14 *Crocidura* species (*n* = 34) and two outgroup specimens (mrn, *S. murinus*, mss, *S. minutissimus*) based on 3D landmark dataset. (**a**) Morphospace along of PC1–PC2 axes (axes describe ca. 60% of the total variance). (**b**) A screenshot of the transformed 3D model at the negative end of the first axis/positive end of the second axis corresponding to *C. arispa* (red dotted line marks a consensus shape outline). (**c**) A screenshot of the transformed 3D model at the positive end of the first axis/positive end of the second axis corresponding to the fossil specimen of *C. lasiura* (FSC RJARV-KorC-93). (**d**) A screenshot of the consensus configuration of the mandibular shape (cons., reference shape); in the current analysis, the consensus corresponds to one of *C. suaveolens* samples (ZIN 73665). (**e**) A screenshot of the transformed 3D model at the positive end of the first axis/negative end of the second axis corresponding to *Suncus murinus*. (**f**) A screenshot of the transformed 3D model at the negative end of the first axis/negative end of the second axis corresponding to *C. sapaensis*. The relative position of the rocky shrews along PC1 is colored. Key: arm, *C. armenica* sample (ZIN 55321, 76972, 76973, 76976; *n* = 4); ars, *C. arispa* (holotype, NHMW 13284); csp, *C. capsica* sample (ZIN 72763, 72764; *n* = 2); h, holotype; gld, *C. gueldenstaedtii* sample (ZIN 72843, 72872; *n* = 2); KRD, the Late Pleistocene specimen of *C. lasiura* (FSC RJARV-KorC-93); las, *C. lasiura* sample (ZIN 76015, 76017, 76020, 76021; *n* = 4); lcd, *C. leucodon* sample (ZIN 72918, 72921; *n* = 2); mrn, *Suncus murinus* (ZIN 15885); mss, *Sorex minutissimus* (ZIN 98582); p, paratype; phg, *C. phanluongi* (ZIN 100307); sha, *C. shantungensis* sample (ZIN 89427, 89433, 89435, 89445; *n* = 4); sib, *C. sibirica* sample (ZIN 79423, 79431, 79437, 79439; *n* = 4); sps, *C. sapaensis* (ZIN 96265); sua, *C. suaveolens* sample (ZIN 73665, 73665, 77220, 98863, 98864; *n* = 5); szr, *C. serezkyensis* (ZIN 77431); *ZIN 6506*, *C. zarudnyi*, holotype (zrd); *ZIN 55321*, *C. armenica*, paratype (arm-t); zts, *C.* ex gr. *kegoensis-zaitsevi* (ZIN 96320). The transformed 3D models were obtained using 3D Slicer ver. 5.4.0 r31938/311cb26.

The first step of the morphospace analyses was based on the mandibular dataset (Figure 4(a^1^,a^2^)). The results of the PCA indicated a clear-cut separation of the *C. arispa* specimen from the other specimens, with a moderate trend toward the mandibular shape of the outgroup specimen of *S. minutissimus* (Figure 8). Along the first principal component (which accounted for 49.13% of the total variance; Figure S21a), we can see an obvious gap between *C. arispa* and the other specimens. The shape changes are mostly associated with the variation in hemimandible proportion, hemimandible elongation toward the *S. minutissimus*/*S. arispa* area (the negative end of the axis), and shortening toward the *S. murinus* area (the positive end). The elongation of the hemimandible is associated with (i) a thinning of the dentary (a decrease in MBH; Table 2) together with (ii) a *Sorex*-like broad inward deflection of the middle part of the dentary (Figure S22) and (iii) a general change in mandibular ramus proportion (Figure 8b,e)). The anterior margin of the coronoid process of the *Sorex*-like morphotype became short and obliquely bent backward. PC2 (11.01%) describes the shape differences between *C. arispa*, *C. leucodon*, and *C. lasiura* (and partly *C. serezkyensis*) at the positive end of this component and the shape of *C. sapaensis* at the negative end of the axis. We tried to explain the shape changes for the second principal component by the alteration of the position of the internal fossa for the temporal muscle: at the positive end, the fossa is shifted posteriorly, and at the opposite end, the fossa is shifted forward (Figure 8b,f)). Moreover, mandibular ramus changes are also related to the variation in the relative position of the condylar process: the condyle shifted downward in relation to the molar level (Figure 8b,c) or upward (Figure 8f). The third principal component (7.33%) described differences in the width of the base of the coronoid process and the variation in general size of the condyle. Along PC3, *C. arispa* and *C. armenica* both occupy a similar area as small-condyle species. The fourth principal component (6.44%) described the shape of the tip of the coronoid process. Figure 8 illustrates an overlap of convex hulls of many species, e.g., *C. armenica* and *C. shantungensis*. Nevertheless, this seems to be only a partial overlap because the 3D plot shows a clear difference of *C. armenica* from the other species, and *C. arispa* is the farthest from *C. armenica* (Figure S23).

To avoid potential misinterpretations of the morphospace results as recently discussed by Polly [33], we used a cluster analysis to check the main patterns of morphospace disparity, namely, the separation of *C. arispa* and *C. armenica* from the other analyzed species. Our analysis by the UPGMA algorithm on the basis of Procrustes coordinates (with a cophenetic correlation = 0.87) supports a distinctive position of *C. arispa* and the partial aggregation of the sample of *C. armenica* individuals. On the other hand, most of the samples, except for the *C. sibirica* and *C. gueldenstaedtii* samples, were found to be mixed among several clusters (Figure S24).

The second step of the morphospace analysis involved the cranial dataset (Figure 4(b^1^–b^5^)). Initially, we had some concerns about the applicability of the damaged skull of the *C. armenica* paratype (ZIN 55321) to the shape analysis. Nonetheless, a general examination of the skull model and linear characteristic values (Table 2) allowed us to include the paratype into our analysis with the following 3D model revisions: we flipped the skull model along the Y plane (MorphoDig tool) to select a more suitable skull side for the landmarking process, and we (roughly) restored an anterior margin of nasal bones for the successful positioning of the third landmark (Figure 4(b^1^–b^5^)).

**Figure 9:**
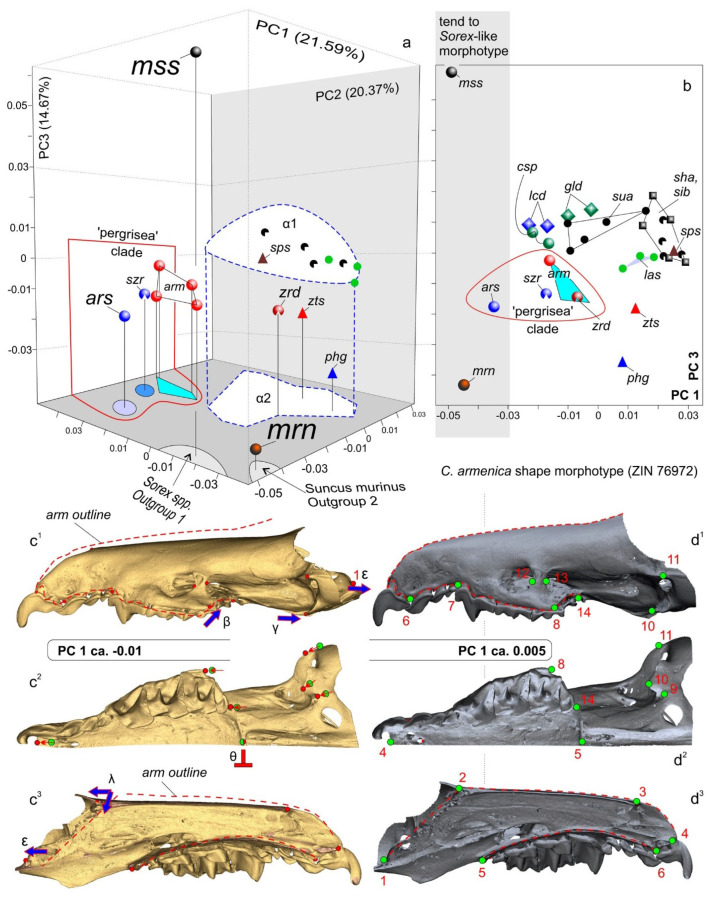
Results of the cranial shape analysis (‘muzzle’ shape) of the 14 *Crocidura* species (*n* = 33) and two outgroup specimens (mrn, *S. murinus*, mss, *S. minutissimus*) on the basis of the 3D landmark dataset. (**a**) Morphospace along of PC1–PC2–PC3 axes (axes describe ca. 57% of the total variance). (**b**) Morphospace along of PC1–PC3 axes (ca. ca. 36%). (**c^1^**–**c^3^**) Screenshots of the transformed 3D model at the negative end of the first component corresponding to *C. arispa* (red dotted line marks the shape outline of *C. armenica*); (**c^1^**), in lateral view; (**c^2^**), in occlusal view; (**c^3^**), in medial view. (**d^1^**–**d^3^**) Screenshots of the transformed 3D model at the positive end of the first component corresponding to *C. armenica*; (**d^1^**), in lateral view; (**d^2^**), in occlusal view; (**d^3^**), in medial view. Key: α^1^, 3D convex hull of ‘non-pergrisea’ species; α^2^, 2D projection of the convex hull on the PC1–PC2 space; β, shape transformation of the zygomatic process of maxilla (8th lm) from *C. armenica* morphotype to *C. arispa* morphotype; γ, shape transformation of the entoglenoid process (10th lm) among *C. armenica*/*C. arispa* morphotypes; ε, shape transformation of the posteroventral margin of the cribriform plate (1st lm) among *C. armenica*/*C. arispa* morphotypes; θ, relatively stable structures, e.g., posterior margin of the hard palate (5th lm); λ, important feature—shape transformation of the dorsal profile of the anterior part of the skull (2nd lm) from more of a high/short profile of *C. armenica* morphotype to more of a low/long profile of *C. arispa* morphotype. For species abbreviations, see Figure 8 and Table 2. The transformed 3D models were obtained using 3D Slicer.

The results of our PCA revealed an obvious separation of all of the specimens of the rocky shrews of *C. arispa*, *C. armenica*, and *C. serezkyensis* from the other analyzed samples. Moreover, *C. arispa* maintained a trajectory of shape changes toward the muzzle shape of *Sorex* along the first principal component (with 21.59% of the total variance; Figure S21b). We also noted that this trajectory is not supported by the other principal components (PC2, 20.37%; PC3, 14.67%). The 3D plot indicates the distinctive position of the sample of rocky shrews (Figure 9a). Additionally, we can infer a more structured morphospace than was revealed for the mandibular dataset, as evidenced by the distinct position of convex hulls, at least those of *C. suaveolens* and *C. lasiura* (Figure 9b).

**Figure 10:**
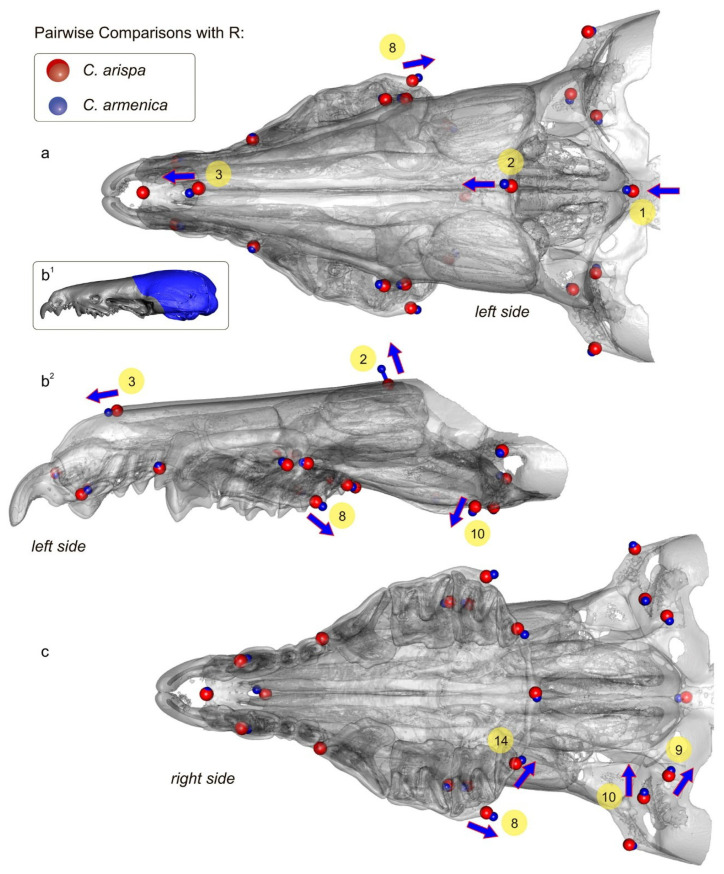
Results of the pairwise comparison of 3D models of the muzzle of *C. arispa* (red landmark spheres) and *C. armenica* (blue spheres) using ‘Morpho’ and ‘rgl’ packages of the R environment. (**a**) A screenshot of the transformed 3D model in dorsal view corresponding to *C. arispa*; the transformations toward the *C. armenica* shape are shown by blue spheres; notable changes are marked by blue arrows. (**b^1^**) A screenshot of the 3D model of the whole *Crocidura* skull in lateral view, with a cut part of the braincase labelled; (**b^2^**) A screenshot of the transformed 3D model in lateral view corresponding to *C. arispa*; (**c**) *ibid*., in ventral view. For the correct transformation of 3D models during particular comparisons using R, the left-side landmark dataset was copied to the right side. Key: 1, first landmark position and associated transformation marks by blue arrow; 2–3, 8–10, 14, landmark positions with transformations (see main text). Unscaled.

When comparing *C. arispa* and *C. armenica*, the shape changes in the muzzle along PC1 are mostly associated with overall muzzle flattening and stretching in the cribriform plate area (transformations toward *C. arispa* in terms of “ε” and “λ” in Figure 9(c^3^)). More precise differences were revealed by the results of our pairwise comparisons using specialized packages of R (Figure 10, Figures S25 and S26). Figure 10 shows similar shape differences in the first and second landmarks’ position: the lowest posteroventral point of the cribriform plate (sphenoethmoidal junction) and uppermost anterodorsal point of the cribriform plate (ethmofrontal junction), respectively. Moreover, we can see differences in terms of the zygomatic processes’ position (Figure 9, “β”; Figure 10, lm 8), composition of the entoglenoid fossa (Figure 9, “γ”; Figure 10, lm 10), and variation in the foramen ovale position (Figure 10, lm 9) and in the size of the nasal aperture (Figure 10, lm 3). The posterior margin of the hard palate is more or less stable (Figure 9, “θ”; Figure 10, lm 5).

Differences between *C. arispa* and *C. serezkyensis* are presented in the Supplementary Materials (Figure S25) and are mostly associated with shape variations in the dorsal profile of the muzzle: *C. serezkyensis* is somewhere between the maximally flat muzzle of *C. arispa* and the relatively “higher” muzzle of *C. armenica*. Nonetheless, our pairwise comparisons between *C. armenica* and *C. serezkyensis* (Figure S26) detected differences in the cribriform plate’s position among the three species (the first and second landmarks).

Just as in the mandibular dataset analysis, we performed a cluster analysis of the skull shape. The analysis by the UPGMA algorithm on the basis of Procrustes coordinates (with a cophenetic correlation = 0.84) surprisingly supports almost all of the samples as distinct clusters or subclusters (Figure S27). The most distinctive muzzle shape belongs to *Crocidura* from Vietnam, *C. phanluongi*, *C. sapaensis*, and *C.* ex gr. *kegoensis-zaitsevi*. The next separate cluster is composed of all specimens of rocky shrews, namely, *C. arispa* and *C. serezkyensis*, and four specimens of the Armenian shrew. Separate subclusters within a common cluster are occupied by *C. lasiura* and *C. sibirica*. The holotype of *C. zarudnyi* is clustered with *C. leucodon* and one specimen of *C. gueldenstaedtii* (ZIN 72843).

## 4. Discussion

### 4.1. General Remarks

Our results somewhat contradict each other, and in the sense of Kuhn’s “puzzle-solving” [78], we have a good “exemplar” [79] of a typical study on fossil material with rare and damaged/fragmented specimens, albeit with a substantive addition of molecular phylogenetic data. Therefore, we are forced to look at our results from two points of view in an attempt to find a middle ground.

The morphospace analysis both for mandibular and cranial datasets revealed that (i) the positions of the *C. armenica* specimens (paratype + new hypodigm) are close in terms of their morphospaces (Figure 8 and Figure 9); (ii) the *C. serezkyensis* specimen in the mandibular morphospace is almost equidistant from *C. armenica* and *C. arispa*, and in the cranial morphospace, it is located between the *C. armenica* convex hull and *C. arispa*; (iii) *C. arispa* occupies a clearly separate position in both morphospaces but trends toward *C. armenica* and *C. serezkyensis* at least in terms of muzzle shape variation.

The reliability of the morphospace results was assessed by a cluster analysis of Procrustes distances for each dataset. The analysis indicated the relatively low reliability of the mandibular shape owing to a mixed combination of clusters of the “*C. suaveolens*” species group (Figure S24); anyway, *C. arispa* maintained a separate position, and *C. armenica*, except for a single specimen, ended up in the same cluster. Conversely, the cluster analysis showed the high reliability of the cranial shape because the main samples were united in specific clusters. Similarly, for the morphospace analyses, the rocky shrews were combined into the same cluster, which was outside that of the other Palearctic species (Figure S27). Similar effects of morphological incongruence were noted recently in *Sorex* species regarding cranial, mandibular, and molar shapes [32].

**Figure 11:**
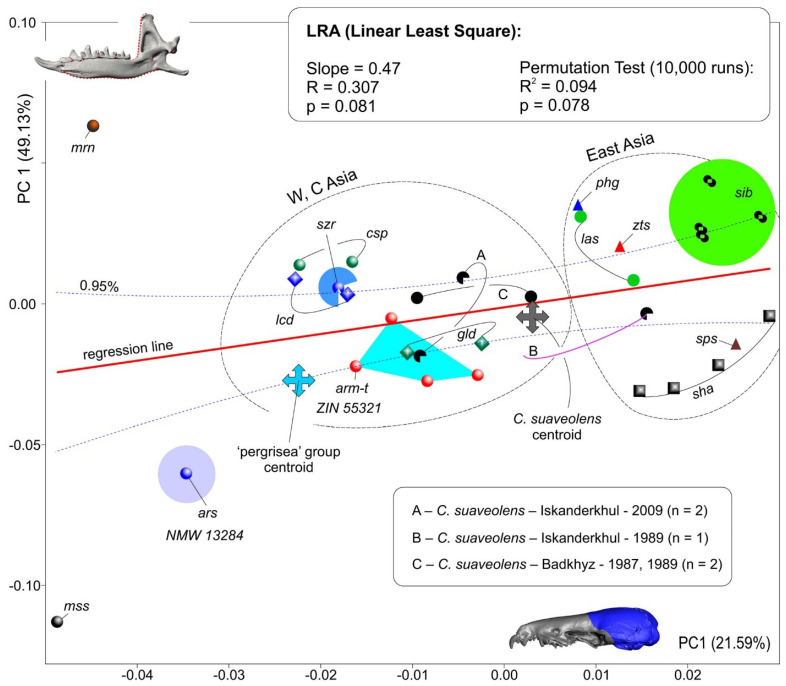
Results of the linear regression analysis (LRA) of two first principal components of the mandibular (ordinate) and cranial (abscissa) datasets. Key: ‘0.95%’, confidence intervals (dotted line); W, C Asia, West and Central Asia. For species abbreviations, see Figure 8 and Table 2.

Despite a different level of morphospace reliability, we see a stable position of the *C. arispa* holotype. For the estimation of the relationships between mandibular and cranial shape variations, we performed a linear regression analysis (LRA). The regression analysis space clearly visualized a shape disparity between the analyzed species/specimens in the combined mode between the two first principal components of the two datasets (Figure 11). We can see a very weak relationship between the two datasets (insignificant r = 0.3; after a permutation test, r was negligible; Figure 11); i.e., the mandible shape varies independently of the muzzle shape. Nevertheless, both independent variables helped us to detect an distinctive position of the *C. arispa* specimen and the separation of all of the specimens into two groups by geography: the first group includes samples from Central and West Asia, and the second includes samples from North and East Asia. In relation to PC scores, the regression space can be interpreted as a notable effect of geographic factors on cranial shape variety, whereas mandibular shape variety—independently from the cranial shape—displays the effects of local factors. The low reliability of the mandibular morphospace that was expressed mostly in the “*C. suaveolens*” group is determined by “phenotypic convergence” [80], when, e.g., the mandibular shape of *C. gueldenstaedtii* along PC1 is similar to the shape of *C. armenica*. The phenotypic convergence is one possible “facet” of the explanation of the phenotypic similarity without revealing deep reasons. The other side of the coin may be represented by Cheverud’s “developmental homoplasy” [81], which can explain the shape convergence via “tinkering” with morphogenetic pathways [82,83] in terms of intrinsic (e.g., phylogenetic relationships) or extrinsic (e.g., same trophic niches) factors. For example, the study on carnivores’ craniomandibular shape convergence by Tamagnini et al. [84] describes “two cases of ecologically equivalent species (i.e., red fox–Malayan civet; raccoon dog–raccoon) converge only in mandibular shape and one case of ecologically similar species of different body sizes converges in both cranial and mandibular shape (i.e., giant and red pandas)”, both in the context of the influence of extrinsic factors.

Our LRA also highlighted a potential problem with cranial-morphospace interpretation. The problem is related to the position of the *C. suaveolens* specimen (ZIN 77220, Iskanderkhul, 1989) in the regression space because its cranial shape trends toward that of the Eastern species samples (Figure 11). We try to explain this in two ways in relation to the taxonomic interpretation: as random (a) or specific (b) effects.

In the case of a small sample size, objects can take distant positions due to a “measurement error” [32] or a random choice of objects from a highly deviated region of the Gaussian distribution (e.g., more than µ + 2δ). The measurement error can be decreased through the repetition of the measurement (landmarking) procedure (see Section 2.6 above). The deviant/unusual position of an object among rare specimens can become a particular/more usual position in a representative sample. Therefore, to avoid the random effects, we should analyze representative samples. If this is impossible, especially for fossil material, we need to estimate centroid positions when sample sizes are small. In the regression space, the centroids of rocky shrews and of the *C. suaveolens* sample are separated by a considerable distance. This approach helps with the overall estimation of mean shapes and differences among them but cannot help determine the taxonomic position of an outlier.

On the other hand, outliers can be determined by the aforementioned developmental tinkering effects. The influence of abundance and of fluctuation of habitat conditions on phenotypic variety is underestimated because it requires a precise selection of samples, which is often impossible. Nevertheless, we know a few papers, including a recent study by Vasiliev et al. [85], in which authors have described a possible relation between a habitat condition change and mandibular shape variance of *C. leucodon* in the temporal aspect (approximately 30+ years, similarly to *C. suaveolens* in our work, between catches). For this type of interpretation, at least one substantial assumption has to be made: the multispecies morphospace has boundaries, which represent the existing limits of a given shape variety in a given analysis. An outlier of a shape beyond the boundaries most likely means a shift to another region of shape variety in the sense of Foote [86] (p. 482). In the context of shrew biology, there is likely an expansion of the trophic niche because most craniomandibular shape transformations of soricids are linked to a foraging advance and trophic specialization [87,88,89].

Perhaps if we work with Quaternary species, which often are present in modern communities or went extinct recently, we may detect the continuous expansion of morphospace boundaries, instead of the separation of a new space area (a new adaptive zone, according to Foote [86], p. 482). Therefore, such boundary expansion may be supported by a taxonomic solution. All other outliers within a relatively continuous morphospace require an additional review but do not merit a taxonomic examination. If we look at the first type of outliers within the modern “morphogenetically based” paradigm [83,90,91,92,93], the extreme shape changes must be translated into a “neomorphic model” of shrew craniomandibular transformations [20,94]. For example, neomorphic changes in cranial shape have been revealed for the Ethiopian endemic shrew, *Crocidura yaldeni*. In this species, neomorphosis and consequently morphospace expansion are linked with muzzle elongation and the relative transformation of skull proportion [20]. A similar “soricinization” [20] trajectory was revealed by us for *C. arispa*, albeit not so prominent as that for *C. yaldeni*, i.e., without a visible change in the antemolar row. Nevertheless, *C. arispa* shows a trajectory in terms of both cranial and mandibular shapes (Figure 11). Moreover, we detected a deep transformation of its dentary to a *Sorex*-like “slender” morphotype (Figure S22). Therefore, the morphometric analysis of shape variety suggests that the morphospace boundary expansion is due to the unique morphology of *C. arispa*. In our paper, this expansion was independently detected in the mandibular and cranial datasets through three separate approaches: a PCA, cluster analysis of reliability, and LRA. The close positions of *C. arispa*, *C. armenica*, and *C. serezkyensis*, especially in the more reliable cranial dataset, allow us to also highlight a similarity in morphogenetic pathways that is based on close phylogenetic relationships (Figure 6).

On the other hand, from our practical work (L.V.), we know of a contrasting example of stable morphospace boundaries in modern soricine shrews and the extinct tribe of Beremendiini (Soricinae, Soricidae). The absence of mandibular morphospace expansion in beremendiin shrews has led to a hypothesis of redeposition of older late Pliocene or Early Pleistocene remains into younger Upper Pleistocene layers, instead of a description of a “new latest beremendiin form” [29].

What seems to follow from the discussion of our morphospace results is that the shape analysis supports, first, the existence of a distinct rocky shrew species group as a whole, and second, that each species of the group deserves a separate taxonomic position at the species level. Both statements are moderately supported by our linear morphometry and analysis of qualitative characteristics.

We found at least two linear measurements that distinguish rocky shrews from sympatric species, namely HCD and LLF. Sympatric species—*C. gueldenstaedtii*, *C. leucodon*, and *C. zarudnyi*—form a large-condyle group with an HCD range of 1.42–1.64 mm and an LLF range of 1.18–1.48 mm. Rocky shrews together with *C. suaveolens* constitute the small-condyle group with an HCD range of 1.07–1.38 mm and an LLF range of 1.00–1.24 mm (Table 4). *C. suaveolens* differ from rocky shrews as they have lower EGW values: *C. suaveolens* have a range of 4.43–6.02 mm, whereas rocky shrews have a range of 6.14–6.54 mm (Table 4). In the *C. suaveolens* samples (localities: Prokhladny, 1985, *n* = 9; Anapa, 1988, *n* = 15; Badkhyz, 1987, 1989, *n* = 2; Iskanderkhul, 1989, 2009, *n* = 3), this characteristic shows homogeneity, which points to its stable variation ranges (Table 3) and hence to the reliability of the difference, at least for *C. suaveolens*.

Species of rocky shrews also have distinctive linear features, which, however, were not tested by the statistical approach here due to the rarity of the specimens used. Nevertheless, we found four linear features that distinguish rocky shrew species amongst themselves, namely MBH, MRH, and the two aforementioned features: HCD and LLF. The first two features were tested for sample homogeneity. In most of the samples that included two to three subsamples (e.g., *C. suaveolens*, *n* = 29; *C. leucodon n* = 12; *C. sibirica*, *n* = 30), and both features had a homogeneous distribution of values (Table 3).

In summary, the lowest MBH value belongs to *C. serezkyensis* (MBH = 1.05 mm); *C. armenica* has a range of 1.12–1.23 mm; and *C. arispa* has the highest value of 1.25 mm (Table 2). The shape analysis revealed the thinnest dentary in *C. arispa*, and the MBH values seem inconsistent. Nonetheless, one should remember that shape transformation on the basis of 3D datasets is more complex than what one can see in 2D images during the interpretation stage. The degree of dentary “slendering” is more or less similar among rocky shrews (Table 2), but that of bending (Figure S22) differs. Moreover, we should simultaneously examine a set of other shape transformations that determine the position of a specimen in the morphospace. In this sense, linear measurements provide a very rough estimate. The lowest MRH belongs to *C. arispa* (MRH = 3.88 mm); *C. serezkyensis* has a slightly larger value of 3.92 mm; and *C. armenica* has the greatest MRH in the group with a range of 4.26–4.33 mm (Table 2). Condylar features relatively clearly distinguish *C. serezkyensis*, which has the lowest HCD (1.07 mm) and the shortest LLF (1 mm). *C. armenica* has a relatively broad range of HCD and LLF values that includes the values of *C. arispa*.

Qualitative characteristics that help to distinguish rocky shrew species were difficult to find. We tried to describe several odontological characteristics; however, a satisfactory result was obtained with only two of them: (i) the size of the first upper incisor root, which distinguishes large-rooted *C. arispa* from relatively small-rooted *C. armenica*. Nonetheless, the small sample size of Armenian shrews provides variation in the root size from the smallest size to a medium size, similarly to that of the root of *C. serezkyensis* (Figure 7); and (ii) the dorsal contour of the *C. arispa* skull has a small downward bend, whereas the contours of *C. armenica* and *C. serezkyensis* are relatively straight (Figure S20). Both characteristics require further investigations with a bigger sample of the material.

Summarising the morphological part of the analysis, we report close positions of rocky shrews and their difference from other *Crocidura* species, including sympatric species. The intragroup relationships that were reconstructed by the morphospace approach detected differences between three analyzed species, in which *C. arispa* is the most distinct species.

If this material had belonged to fossil species and there were no phylogenetic data, then we would have made a firm conclusion about the taxonomic independence of each species of the group.

It is likely that the availability of phylogenetic data will correct taxonomic conclusions based on morphological data. A shining example of this thesis in relation to Late Pleistocene shrew species was the correction of the taxonomic composition of some European faunal complexes through a demonstration of conspecificity between the large *Sorex macrognathus* Janossy, 1965 and medium-sized *Sorex araneus* Linnaeus, 1758 on the basis of an analysis of ancient DNA [95]. Modern fauna has an incomparably larger number of examples of corrections of morphology-based taxonomy by molecular data [37,96]. We have to discuss this because our phylogeny based on the *cytb* gene only partially validates our morphology-based conclusions. The phylogenetic analysis revealed a separate position of the “*Crocidura pergrisea*” species complex with the stable support of this clade (Figure 6). This result was confirmed by the morphospace and cluster analyses, at least for the cranial dataset and for three of the four species in the phylogenetic dataset (*C. ramona* is absent in the morphological comparisons). Nevertheless, the species independence of *C. arispa*/*C. armenica* on the basis of short genetic distances (2.8%) can be questioned. In addition, the p-distance between *C. arispa*/*C. armenica* and *C. serezkyensis* is also short and amounts to only 3.4% (Figure 6b), which is less than that revealed, e.g., between *C. suaveolens* and *C. caspica* (5.3%) [16].

### 4.2. Taxonomic Remarks

To sum up the current analysis, which combines several approaches of morphological investigation against the background of the *cytb*-based phylogeny, we have to make a difficult taxonomic decision about the status of species of the “pergrisea” group, e.g., *C. arispa*, *C. armenica*, and *C. serezkyensis*, owing to the incongruence between short genetic distances at the subspecies level and well-defined morphological differences at the species level. How should one find a balance and decide on a taxonomy? To answer this question, we need to identify two essential aspects to determine our choice.

The first aspect deals with historical mitochondrial introgression and possible interspecies hybridization, which have been revealed within several shrew genera. Konečný et al. [14] have supposed hybridization between several species of Ethiopian *Crocidura*. Meegaskumbura and Schneider [22] have revealed cases of intergroup hybridization among Asian species of *Suncus* Ehrenberg, 1832. These examples possibly indicate there are still hidden hybridization effects—in other groups of crocidurines—that can be revealed by a more precise phylogenetic analysis with the addition of nuclear datasets. Unfortunately, due to hybridization, a single-gene phylogeny based on *cytb* can hide a real relationship between taxa, and therefore, we have a reason not to trust the short genetic distances within the “pergrisea” group. We are hoping for a subsequent multi-gene analysis involving nuclear genes because relatively long branches between *C. arispa* and *C. serezkyensis* in terms of nuclear genes (*ApoB* and *BRCA1* exons) have been noted in a recent work by Bannikova et al. [16].

The second aspect is the incomplete representativeness of the samples of species under study. Our colleagues [16] have analyzed three species—*C. ramona*, *C. serezkyensis*, and *C. arispa*—without *C. armenica*. Here, we also analyzed three species—*C. serezkyensis*, *C. arispa*, and *C. armenica*—but without *C. ramona*. Moreover, we should not forget about the fifth species of the group, *C. pergrisea*, which is highly “enigmatic” and requires morphological re-description and inclusion in molecular analyses.

Judging by our current results and taking the two aforementioned aspects into account, we propose keeping a separate species-level status for *C. serezkyensis* and *C. arispa*. Furthermore, we wish to restore the species-level status of *C. armenica*. This taxonomic decision is based on the precise analysis of the morphological data, which revealed within rocky shrews unique transformations in their craniomandibular shape that supposedly helped them with the transition to the new area of morphospace/trophic niches and consequently separated them from other analyzed *Crocidura* groups. This decision is tentative because a final decision will require (i) a new phylogenetic multi-gene analysis of all available specimens of key species and (ii) the expansion of the morphospace because of the additional key species, including at least *C. ramona* and preferably “true” *C. pergrisea*.

We think that our decision to maintain the status of separate species for the analyzed rocky shrews is similar to the decision of Bannikova et al. [16] in that they did not propose to synonymize *C. arispa* and *C. serezkyensis* either because of the incompleteness of the species list.

Furthermore, we would like to say that our colleagues [16] have suggested regarding *C. arispa* and *C. serezkyensis* as a “single superspecies consisting of a number of recently diverged and geographically isolated allospecies. “If we look at the “superspecies” and “allospecies” in the paleontological context, then we will not find any basis for introducing such taxonomic categories. In case of doubt in the taxonomic status of a form, we may use the open nomenclature. With respect to rocky shrews, the word “superspecies” can be replaced by “*Crocidura* ex gr. *serezkyensis*” owing to species name priority: Laptev in 1929 described *C. serezkyensis*; Gureev in 1963 described *C. armenica*; and Spitzenberger in 1971 described *C. arispa*. However, in this case, we will be forced to use the open nomenclature for some other *Crocidura* groups, for example, *C. suaveolens*/*C. sibirica*.

### 4.3. Cybertaxonomy of Shrews: Pipeline Development

Our precise description of the craniomandibular and dental characteristics of the *C. armenica* paratype, including its teratological aspects and hidden features of the lower-teeth endodont (which were first described herein), show the “normal state” of its dental morphology, which previous researchers have ignored due to the poor condition of the skull [17,19]. Moreover, the dental developmental abnormalities of the *C. armenica* holotype (Figure 5) deserve a separate study as an extraordinary example of the stability of tribosphenic tooth morphogenesis, in which developmental deviations at early stages of tooth morphogenesis toward the later stages have been gradually covered. As a result, we have an abnormal crown part of the left and right m1 and a more-or-less normal root part, at least for the right m1 (Figure 4(b^1^)).

These findings are no less significant than the taxonomic conclusions made above. CTtax offers new possibilities for comparisons and meticulous analyses of materials. Within this framework, the standardization of obtaining raw data has become very relevant and important. The current paper offers the first documented standardization protocol for the description of mammalian cybertypes.

**Figure 12:**
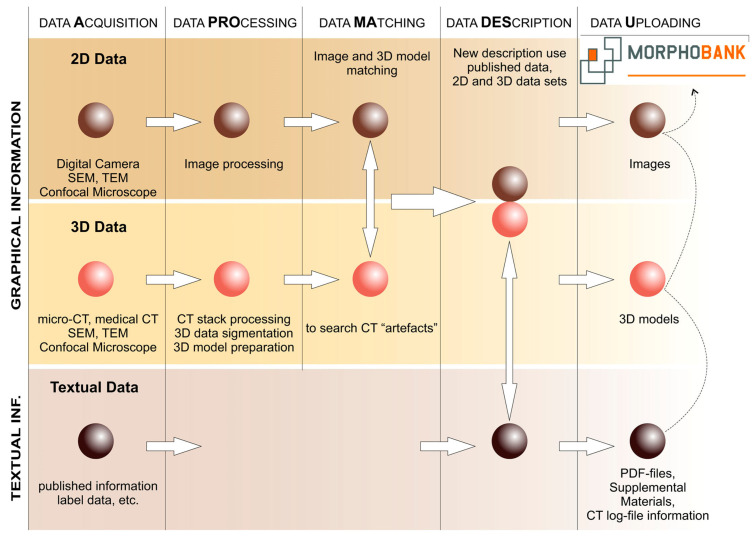
A schematic depiction of the five steps and three levels of the AProMaDesU pipeline in the basic CTtax approach: A—data acquisition; Pro—data processing; Ma—data matching; Des—data description; U—data uploading into specialized digital repository. For details, see Section 1.2 and Section 4.3.

In the introduction section, we were guided by Faulwetter et al. [23] and expanded the basic requirements for micro-CT-based cyber-datasets in relation to mammalian collections. Accordingly, we developed a routine pipeline for the application of the cybertaxonomic approach to type specimens of small mammals. The pipeline was named “AProMaDesU“ in accordance with the five steps in data processing: (A) data acquisition stage using a digital camera for 2D images and a micro-CT scanner for 3D models; (Pro) data processing stage with the help of particular APPs; (Ma) data matching stage to assign 2D and 3D datasets; (Des) data description stage for the precise description of 2D and 3D datasets; and (U) data uploading stage to a specialized digital repository, e.g., MorphoBank (https://morphobank.org/; accessed on 1 May 2024), MorphoSource (https://www.morphosource.org/; accessed on 1 May 2024), and MorphoMuseuM (https://morphomuseum.com/; accessed on 1 May 2024). Each step includes three information levels, such as 2D and 3D data (visual sources) and textual data (Figure 12). The AProMaDesU protocol fully satisfies the five basic requirements for micro-CT-based cyber-datasets in relation to mammalian collections.

In this paper, for the first time, we implemented all five requirements. (i) Both cybertypes of *C. armenica*, the holotype (ZIN 45277) and paratype (ZIN 55321), are “maximally fitted” to physical type materials because of the high resolution of the detailed micro-CT scans, followed by precise reconstruction of 3D models. Nonetheless, the fulfillment of this condition depends not only on the spatial resolution of the CT scans but also on the settings of the scanning procedure (the choice of filter and many other parameters) and the conditions of the postprocessing of the scan volumes. For example, the initially unassessed imbalance of denser tooth matter (i.e., greater X-ray absorption) and lighter parts (squama) of the sphenoid, parietal, and ethmoidal of the *C. arispa* holotype produced many artifacts in the 3D model. In this case, to decrease the density-related effects, we carried out the reconstruction of anterior and posterior parts of the skull with different settings of the “Surface Generation” tool (Avizo). As a result, the 3D model of the *C. arispa* holotype has a distinctive artefact, such as a ledge around the entire circumference of the orbital part (Figure S28). (ii) The cybertypes of *C. armenica* were created by a nondamaging technique which—together with our additional requirement (iv) about accompanying images of a physical type—enables the use of the model and images for a detailed description of various phenotypic traits (Figure 4, Figures S15 and S16) similarly to the materials published by Voyta et al. [30], Zazhigin and Voyta [70]), Arnaudo et al. [97], Skandalos and van den Hoek Ostende [98], and many others. The models can be employed without physical access to the type material in theriological collections. (iii) All the models analyzed here (Table S1) are available in MorphoBank (http://www.morphobank.org (accessed on 1 May 2024); project No. 4964). The cybertypes, such as tables with linear dimensions, photos, and explanatory images in the Supplementary Materials (e.g., Figure S5), are also available in our MorphoBank project. Requirement “iv” has already been mentioned above. (v) The Supplementary Materials of the current study (Table S1) contain full additional information about the scanning procedure, the scanner model, and settings for each object. This information is available in MorphoBank, project 4964.

## 5. Conclusions

The extraction of museum DNA from unique crocidurine material from the theriological collections at ZMMU [16] and ZIN (this study) is a significant step forward in our knowledge of the enigmatic “*Crocidura pergrisea*” species group from Central and West Asia. In the present study, a combination of a molecular analysis of the museum material with a micro-CT-based cybertaxonomic revision of the type material and a comprehensive morphospace analysis shed new light on the question of the validity of the Armenian shrew species and expanded its geographic range from type locality to a new locality near Julfa (Azerbaijan). Moreover, our findings uncovered significant differences—in cranial and mandibular shapes among three species of rocky shrews (*C. armenica*, *C. arispa*, and *C. serezkyensis*)—despite the relatively small genetic distances deduced from the *cytb* gene. Because the genetic divergence of the group requires verification using a dataset of nuclear genes, our taxonomic conclusion is mostly based on the morphological comparisons and features of the multivariate morphospace. Finally, we believe that the “*C. pergrisea*” species group is composed of five valid Central and West Asian species: *C. armenica*, *C. arispa*, *C. pergrisea*, *C. ramona*, and *C. serezkyensis*, whose taxonomic levels still need further clarification by means of additional samples of *C. pergrisea* and *C. ramona* (morphological comparisons) and molecular datasets of *C. armenica* and *C. pergrisea*. The correctness of such analyses will be based on an assessment of the magnitude of differences between and within the species groups of “*C. pergrisea*” and “*C. suaveolens*”.

## Figures and Tables

**Table 1:** The list of *cytb* sequence data of *Crocidura* used in the assessment of the phylogenetic position of *C. armenica*. ^S3^, locality details (see in the Supplementary Materials; Table S3); IZEA, Institut de Zoologie et d’Ecologie Animale, Lausanne, Switzerland; NHMW, Natural History Museum Vienna, Vienna, Austria; TAU, Tel-Aviv University; ZIN, Zoological Institute of the Russian Academy of Sciences, St. Petersburg, Russia; ZMMU, Zoological Museum of the Moscow State University, Moscow, Russia. Sequence data marked with a question mark came from GenBank without source information. Bolded IDs are our data.

Species/Collection Number of Voucher/spp. Abbr.	Locality	GenBank ID
*C. armenica*, holotype, ZIN 45277 (ZIN-TER-M-5876); arm	Garni ^S3^, Armenia;	**OR449074**
*C. armenica*, ZIN 77972 (ZIN-TER-M-5877); arm_972	Julfa ^S3^, Nakhchivan Autonomous Republic of Azerbaijan;	**OR449075**
*C. arispa*, holotype, NHMW 13284; ars	Niğde ^S3^, Turkey;	OP599553 [17]
*C. caspica*, IZEA 7793; csp	Azerbaijan;	AY843487 [49]
*C. gueldenstaedtii*, IZEA 2687; gld	Dusheti, Georgia;	AY843497 [49]
*C.* ex gr. *kogoensis-zaitsevi*, ZIN 96320; zts	Ngoc Linh ^S3^, Vietnam;	HM587003 [37]
*C. lasiura*, ?; las	Kraskino, Primorye, Russia;	AB077072 [50]
*C. leucodon*, IZEA 23629; lcd	Alazani, Georgia;	DQ994756 [51]
*C. phanluongi*, holotype, ZIN 97092; php	Yok Don ^S3^, Vietnam;	HM587020 [52]
*C. ramona*, TAUM12771	Israel;	LR536374 [?]
*C. sapaensis*, ZIN 97792; sps	Sa Pa ^S3^, Vietnam;	MW075591 [?]
*C. serezkyensis*, ZIN 77431 (ZIN-TER-M-5875); szr	Sarez Lake ^S3^, Tajikistan;	**OR449076**
*C. serezkyensis*, ZMMU S-111841; szr1	Tajikistan, Pamir, Peter I Range, Pashimgar;	OP599554 [17]
*C. serezkyensis*, ZMMU S-111842; szr2	*ibid*.;	OP599555 [17]
*C. shantungensis*, ?; sha	Popov Island, Primorye, Russia;	AB077278 [49]
*C. sibirica*, ZMMU S- 177776; sib	Krapivinsk, Kemerovo Oblast’, Russia;	AY994389 [53]
*C. suaveolens*, ZIN 73479	Krasnodar Krai, Russia;	AY843476 [49]
*C. suaveolens*, ZIN 73756; sua_756	Badhyz ^S3^, Turkmenistan;	AY843479 [49]
*C. suaveolens*, ZIN 77220; sua_220	Iskanderkhul ^S3^, Tajikistan;	AY843482 [49]
*C. zarudnyi*, I-89; zrd	Pir Sohrab, Iran;	AY925211 [54]
*Suncus murinus*, ?; mrn	Okinawa, Japan.	AB175074 [55]
**Total: 15 spp.; *n* = 21**		

**Table 2:** Linear dimensions (mm) of analyzed specimens of the target “pergrisea” species group and same-size species: *C. gueldenstaedtii*, *C. leucodon*, *C. suaveolens*, and *C. zarudnyi*. Information is in the following order: m, mean/SE, standard error of mean/lim, limits range/SD, standard deviation/n, sample size. Key: arm, *C. armenica* (holotype, ZIN 45277); arm-t, *C. armenica* (paratype, ZIN 55321); arm_972, *C. armenica* (ZIN 77972); arm_973, *C. armenica* (ZIN 77973); arm_976, *C. armenica* (ZIN 77976); ars, *C. arispa* (holotype, NHMW 13284); C, tail length; CIL, condylo-incisive length; EGW, external entoglenoid width; gld, *C. gueldenstaedtii*; HB, head and body length; lcd, *C. leucodon*; LML, lower molar length; MBH, mandibular body height; MRH, mandibular ramus height; P4s/d, interval between inner margins of the right and left P4 (Figure S1); Pl., hindfoot length; PL, hard palate length; sua, *C. suaveolens*; szr, *C. serezkyensis* (ZIN 77431); UML, upper molar row length; zrd, *C. zarudnyi* (holotype, ZIN 6506); ZYG, external width of zygomatic processes of maxilla. ^1^ In the first description of *C. arispa* by Spitzenberger [76], the hindfoot length was “42.5”. Exactly, it was typo; ^2^ Data by Gureev [75]; ^3^ By collector label; ^4^ Data by Zaitsev et al. [19]; ^5^ Data by Ognev [77].

Species, Specimens	MBH	MRH	LML	CIL
1. arm	1.11	4.26	3.48	n.a.
2. arm-t	1.22	4.33	3.80	n.a.
3. arm_972	1.23	4.30	3.88	17.96
4. arm_973	1.23	4.33	3.81	19.06
5. arm_976	1.12	4.29	3.79	18.88
6. ars	1.25	3.88	3.78	17.92
7. szr	1.05	3.92	3.86	17.91
8. gld	1.19 ± 0.02/0.11/ 0.97–1.39/30	4.4 ± 0.05/0.30/ 3.68–4.89/30	3.93 ± 0.03/0.21/ 3.34–4.25/30	17.95 ± 0.21/1.08/ 15.39–19.52/25
9. lcd	1.34 ± 0.02/0.09/ 1.22–1.50/12	4.72 ± 0.05/0.17/ 4.42–4.97/12	4.06 ± 0.04/0.16/ 3.80–4.38/12	18.4 ± 0.18/0.65/ 17.42–19.24/12
10. sua	1.13 ± 0.01/0.07/ 0.98–1.25/29	4.21 ± 0.03/0.18/ 3.73–4.58/29	3.78 ± 0.02/0.09/ 3.63–3.98/29	16.99 ± 0.08/0.40/ 16.22–17.78/28
11. zrd	1.49	3.95	4.70	18.8
	PL	UML	ZYG	EGW
1. arm	6.92	2.93	5.22	n.a.
2. arm-t	6.95	3.04	5.41	6.21
3. arm_972	7.14	3.18	5.56	6.24
4. arm_973	7.61	3.41	6.14	6.54
5. arm_976	7.63	3.42	5.89	6.30
6. ars	7.31	3.10	5.37	6.14
7. szr	7.43	3.27	5.59	6.17
8. gld	7.66 ± 010/0.52/ 6.27–8.39/27	3.27 ± 0.04/0.21/ 2.74–3.54/27	5.64 ± 0.06/0.36/ 4.79–6.30/27	5.95 ± 0.07/0.40/ 5.07–6.57/27
9. lcd	7.88 ± 0.08/0.29/ 7.33–8.32/12	3.31 ± 0.03/0.13/ 3.08–3.49/12	6.17 ± 0.06/0.22/ 5.87–6.53/12	6.35 ± 0.05/0.20/ 6.07–6.74/12
10. sua	7.18 ± 0.05/0.25/ 6.74–7.72/29	3.15 ± 0.02/0.09/ 2.98–3.28/29	5.46 ± 0.03/0.14/ 5.19–5.70/29	5.71 ± 0.03/0.18/ 5.43–6.02/29
11. zrd	7.99	3.34	6.06	6.32
	P4s/d	HB	C	Pl.
1. arm	0.91	60.0 ^2^	45.0	12.0
2. arm-t	0.97	—	—	—
3. arm_972	0.98	57.0 ^3^	41.0	11.6
4. arm_973	1.13	65.0 ^3^	50.5	11.4
5. arm_976	1.12	-3	-	-
6. ars	1.04	70.0	48.0	12.5 ^1^
7. szr	1.14	62.0	48.0	10.0
8. gld	1.17 ± 0.02/0.11/ 0.86–1.42/27	Lim. 57–80 ^4^	Lim. 41–53	Lim. 11–14
9. lcd	1.13 ± 0.01/0.06/ 1.04–1.22/12	Lim. 59–82 ^4^	Lim. 31–39	Lim. 12–15
10. sua	1.17 ± 0.02/0.08/ 0.99–1.31/28	Lim. 47–74 ^4^	Lim. 25–40 ^4^	Lim. 9–13 ^4^
11. zrd	1.32	60.8 ^5^	47.5 ^5^	13.0 ^5^

**Table 3:** Homogeneity of the analyzed samples (*n* ≥ 7) as estimated by the parametric Shapiro–Wilk normality test (W). Negative test results (=sample is nonhomogeneous) are boldfaced. Expanded results, composed of the full set of the normality results (Anderson-Darling, Jarque-Bera, and some others), are provided in the Supplementary Materials (Tables S2–S9). * Values are in the following order: W-value/*p*-value/*n*.

Species	MBH *	MRH	LML	CIL
*C. gueldenstaedtii*	0.97/0.76/30	**0.88/0.004/30**	**0.48/<0.001/30**	**0.86/0.003/25**
*C. lasiura*	**0.84/0.01/14**	**0.85/0.01/17**	**0.84/0.008/17**	**0.83/0.006/17**
*C. leucodon*	0.92/0.33/12	0.93/0.47/12	0.98/0.98/12	0.87/0.08/12
*C. sapaensis*	**0.58/<0.001/11**	0.94/0.60/11	0.97/0.89/11	0.93/0.48/11
*C. shantungensis*	0.93/0.16/23	0.97/0.85/22	0.96/0.49/23	0.96/0.61/23
*C. sibirica*	0.97/0.67/30	0.94/0.16/30	**0.92/0.04/29**	0.93/01./27
*C. suaveolens*	0.98/0.88/29	0.97/0.65/29	0.96/0.54/27	0.97/0.64/28
*C. zaitsevi*	0.96/0.85/7	0.89/0.30/7	0.89/0.30/7	0.98/0.98/7
Species	PL	UML	ZYG	EGW
*C. gueldenstaedtii*	**0.89/0.009/27**	**0.89/0.01/27**	0.94/0.17/27	**0.91/0.02/27**
*C. lasiura*	**0.89/0.04/17**	0.96/0.79/17	0.83/0.008/16	**0.85/0.01/17**
*C. leucodon*	0.94/0.49/12	0.91/0.26/12	0.93/0.40/12	0.96/0.82/12
*C. sapaensis*	0.94/0.55/11	0.93/0.51/11	**0.83/0.02/11**	0.89/0.16/11
*C. shantungensis*	0.96/0.54/23	0.97/0.90/23	0.95/0.44/23	0.93/0.14/23
*C. sibirica*	0.95/0.19/29	0.95/0.19/29	0.96/0.54/29	0.96/0.52/29
*C. suaveolens*	0.96/0.53/29	0.93/0.07/29	0.96/0.33/29	0.94/0.10/29
*C. zaitsevi*	0.94/0.67/7	0.81/0.05/7	0.85/0.13/7	0.92/0.49/7

**Table 4:** Linear dimensions (mm) of the mandibular condylar process of analyzed specimens of the target “pergrisea” species group and same-sized species, *C. gueldenstaedtii*, *C. leucodon*, *C. suaveolens* and *C. zarudnyi*. *Key*: ^B^, Badkhyz locality (Turkmenistan); ^I^, Iskanderkhul Lake locality (Tajikistan; Table S3); HCD, condylar height; LLF, lower facet length (Figure S1); *, similar values between *C. armenica* and *C. arispa*.

Specimens	HCD	LLF
1. *C. armenica*, ZIN 45277	1.29	1.19
2. *C. armenica*, ZIN 55321	1.20 *	1.19
3. *C. armenica*, ZIN 77972	1.34	1.19
4. *C. armenica*, ZIN 77973	1.31	1.18
5. *C. armenica*, ZIN 77976	1.38	1.13 *
6. *C. arispa*, NHMW 13284	1.19 *	1.13 *
7. *C. serezkyensis*, ZIN 77431	1.07	1.00
8. *C. gueldenstaedtii*, ZIN 72843	1.64	1.27
9. *C. gueldenstaedtii*, ZIN 72872	1.42	1.18
10. *C. leucodon*, ZIN 72918	1.60	1.48
11. *C. leucodon*, ZIN 72921	1.58	1.46
12. *C. suaveolens*, ZIN 73665 ^B^	1.33	1.24
13. *C. suaveolens*, ZIN 73756 ^B^	1.29	1.17
14. *C. suaveolens*, ZIN 77220 ^I^	1.26	1.13
15. *C. suaveolens*, ZIN 98863 ^I^	1.31	1.14
16. *C. suaveolens*, ZIN 98864 ^I^	1.27	1.17
mean: *C. suaveolens*, *n* = 5	1.29 ± 0.01/0.02 1.26–1.33	1.17 ± 0.01/0.04/ 1.13–1.24
17. *C. zarudnyi*, ZIN 6506	1.54	1.34

## Data Availability

The morphological data presented in the current study are available within the article and Supplementary Materials, as well as in MorphoBank Projects 3885 (http://dx.doi.org/10.7934/P3885; accessed on 1 May 2024) and 4964 (https://morphobank.org/index.php/Projects/ProjectOverview/project_id/4964; accessed on 1 May 2024).

## Supplementary Materials

The following supporting information can be downloaded at: https://www.mdpi.com/article/10.3390/biology13060448/s1. Supplementary material sets include the following: Table S1, List of three-dimensional models used in the study and micro-CT scanning technical properties. NEOSCAN N80 (CCD) and SkyScan 1172 (CCD) scanners were a part of the facilities of the Resource Centre for X-ray Diffraction Studies of Saint Petersburg State University (Saint Petersburg, Russia); YXLON FF35 CT (XRD 4343CT) is a part of the facilities of the Natural History Museum Vienna (Vienna, Austria); Table S2, List of specimens used in the study; Table S3, List of localities; Table S4, Univariate statistics of nine linear dimensions of the *C. gueldenstaedtii* sample (*n* = 30) and values of 3D model specimens; Table S5, Univariate statistics of nine linear dimensions of the *C. lasiura* sample (*n* = 14) and values of 3D model specimens, including fossil KRD (FSC RJARV-KorC-93); Table S6, Univariate statistics of nine linear dimensions of the *C. leucodon* sample (*n* = 12) and values of 3D model specimens; Table S7, Univariate statistics of nine linear dimensions of the *C. sapaensis* sample (*n* = 11) and values of 3D model specimens; Table S8, Univariate statistics of nine linear dimensions of the *C. shantungensis* sample (*n* = 23) and values of 3D model specimens; Table S9, Univariate statistics of nine linear dimensions of the *C. sibirica* sample (*n* = 30) and values of 3D model specimens; Table S10, Univariate statistics of nine linear dimensions of the *C. suaveolens* sample (*n* = 29) and values of 3D model specimens; Table S11, Univariate statistics of nine linear dimensions of the *C. zaitsevi* sample (*n* = 7) and values of *C*. ex gr. *kegoensis-zaitsevi* (ZIN 96320); Table S12, Homogeneity of the *C. gueldenstaedtii* sample (*n* = 30) estimated by set of parametric (W, JB) and non-parametric (A) normality tests, calculated using PAST (ver. 4.03; Hammer et al. 2001); Table S13, Homogeneity of the *C. lasiura* sample (*n* = 14); Table S14, Homogeneity of the *C. leucodon* sample (*n* = 12); Table S15, Homogeneity of the *C. sapaensis* sample (*n* = 11); Table S16, Homogeneity of the *C. shantungensis* sample (*n* = 23); Table S17, Homogeneity of the *C. sibirica* sample (*n* = 30); Table S18, Homogeneity of the *C. suaveolens* sample (*n* = 29); Table S19, Homogeneity of the *C. zaitsevi* sample (*n* = 7); Figure S1, Linear measurement frame and diagrammatic image of the ‘glass-plate tool’ for use with flatbed table scanner; Figure S2, An example of the use of the glass-plate tool (GPL); Figure S3, Steps for combining two mandibular parts through the functions of the Avizo software; Table S20, Definition of landmarks used in the shape analysis of hemimandible; Table S21, Definition of landmarks used in the shape analysis of skull; Figure S4, Explanatory drawings of the landmarks’ position on the premaxilla and maxilla bones’ surface (a) and inner surface of the cranium (b); Figure S5, *Crocidura armenica* ZIN 45277, skull fragment; Figure S6, *Crocidura armenica* ZIN 45277, skull; Figure S7, *Crocidura armenica* ZIN 45277, skull fragment; Figure S8, *Crocidura armenica* ZIN 45277, upper teeth; Figure S9, *Crocidura armenica* ZIN 45277, left hemimandible and lower teeth; Figure S10, *Crocidura armenica* ZIN 45277, right second lower molar (unscaled); Figure S11, *Crocidura armenica* ZIN 45277, right hemimandible and lower teeth; Figure S12, *Crocidura armenica* ZIN 55321, skull fragment; Figure S13, *Crocidura armenica* ZIN 55321, skull; Figure S14, *Crocidura armenica* ZIN 55321, skull; Figure S15, *Crocidura armenica* ZIN 55321, skull; Figure S16, *Crocidura armenica* ZIN 55621, left hemimandible and lower teeth; Figure S17, *Crocidura armenica* ZIN 45277 (above) and *C. arispa* NMW 13284 (below), dry stuffed skins; Figure S18, *Crocidura serezkyensis* ZIN 77431 dry skin; Figure S19, Bivariate (a) and univariate (b, c) plots of linear characteristic comparisons of 14 *Crocidura* species, including species of ‘pergrisea’ group and two outgroup species, *Suncus* and *Sorex*; Figure S20, Comparison of skulls; Figure S21, General information on the principal component analyses performed for the mandibular shape (a) and cranial shape (b) comparisons; Figure S22, Mandibular shape transformation: explanation of the dorsal profile changes for first component between positive end (above; *Suncus*-like morphotype) and negative end (below; *Sorex*-like morphotype); Figure S23, 3D plot of the mandibular shape morphospace along PC1–PC3 axes in two projections; Figure S24, Result of the cluster analysis of Procrustes distances of the mandibular 3D shape dataset of 36 specimens of shrews; Figure S25, Result of the pairwise comparison of two 3D models of *C. arispa* (red) and *C. serezkyensis* (green) of the muzzle using ‘Morpho’ and ‘rgl’ packages of the R environment; Figure S26, Result of the pairwise comparison of two 3D models of *C. serezkyensis* (green) and *C. armenica* (blue) of the muzzle using ‘Morpho’ and ‘rgl’ packages of the R environment; Figure S27, Result of the cluster analysis of Procrustes distances of the muzzle 3D shape dataset of 35 specimens of shrews; Figure S28, Screenshots of two 3D models of the skull of *Crocidura arispa* (holotype, NHMW 13284) under same-setting (a) and separate-setting (b) segmentation.

## References

[1] Burgin C.J., Colella J.P., Kahn P.L., Upham N.S. (2018). How many species of mammals are there?. J. Mammal..

[2] Upham N.S., Esselstyn J.A., Jetz W. (2019). Inferring the mammal tree: Species-level sets of phylogenies for questions in ecology, evolution, and conservation. PLoS Biol..

[3] Hutterer R., Wilson D.E., Reeder D.A. (2005). Order Soricomorpha. Mammal Species of the World: A Taxonomical Reference.

[4] Burgin C.J., He K., Wilson D.E., Russell A.M. (2018). Family Soricidae. Handbook of the Mammals of the World. Insectivores, Sloths and Colugos.

[5] Jenkins P.D., Abramov A.V., Rozhnov V.V., Makarova O.V. (2007). Description of two new species of white-toothed shrews belonging to the genus *Crocidura* (Soricomorpha: Soricidae) from Ngoc Linh Mountain, Vietnam. Zootaxa.

[6] Jenkins P.D., Lunde D.P., Moncrieff C.B. (2009). Descriptions of new species of *Crocidura* (Soricomorpha: Soricidae) from Mainland Southeast Asia, with synopses of previously described species and remarks on biogeography. Bull. Am. Mus. Nat. Hist..

[7] Jenkins P.D., Abramov A.V., Rozhnov V.V., Ollson A. (2010). A new species of *Crocidura* (Soricomorpha: Soricidae) from southern Vietnam and north-eastern Cambodia. Zootaxa.

[8] Jenkins P.D., Abramov A.V., Bannikova A.A., Rozhnov V.V. (2013). Bones and genes: Resolution problems in three Vietnamese species of *Crocidura* (Mammalia, Soricomorpha, Soricidae) and the discription of an additional new species. Zookeys.

[9] Abramov A.V., Jenkins P.D., Rozhnov V.V., Kalinin A.A. (2008). Description of a new species of *Crocidura* (Soricimorpha: Soricidae) from the island of Phu Quoc, Vietnam. Mammalia.

[10] Lavrenchenko L.A., Voyta L.L., Hutterer R. (2016). Diversity of shrews in Ethiopia, with the description of two new species of *Crocidura* (Mammalia: Lipotyphla: Soricidae). Zootaxa.

[11] Demos T.C., Achmadi A.S., Handika H., Maharadatunkamsi, Rowe K.C., Esselstyn J.A. (2017). A new species of shrew (Soricomorpha: *Crocidura*) from Java, Indonesia: Possible character displacement despite interspecific gene flow. J. Mammal..

[12] Zhang H., Wu G.Y., Wu Y.Q., Yao J.F., You S., Wang C.C., Cheng F., Chen J.P., Tang M.X., Li C.L. (2019). A new species of the genus *Crocidura* from China based on molecular and morphological data (Eulipotyphla: Soricidae). Zool. Syst..

[13] Yang L., Zhang H., Zhang C., Wu J., Wang Z., Li C., Zhang B. (2020). A new species of the genus *Crocidura* (Mammalia: Eulipotyphla: Soricidae) from Mount Huang, China. Zool. Syst..

[14] Konečný A., Hutterer R., Meheretu Y., Bryja J. (2020). Two new species of *Crocidura* (Mammalia: Soricidae) from Ethiopia and updates on the Ethiopian shrew fauna. J. Vertebr. Biol..

[15] Kamalakannan M., Sivaperuman C., Kundu S., Gokulakrishnan G., Vinkatraman C., Chandra K. (2021). Discovery of a new mammal species (Soricidae: Eulipotyphla) from Narcondam volcanic island, India. Sci. Rep..

[16] Bannikova A.A., Lisenkova A.A., Solovyeva E.N., Abramov A.V., Sheftel B.I., Kryštufek B., Lebedev V.S. (2023). The first phylogenetic data on the elusive shrews of the *Crocidura pergrisea* species complex. Hystrix.

[17] Zaitsev M.V. (1991). Species composition and questions of systematics of white-toothed shrews (Mammalia, Insectivora) of the fauna of USSR. Zool. Inst. USSR Acad. Sci..

[18] Kryštufek B., Vohralĺk V. (2001). Mammals of Turkey and Cyprus: Introduction, Checklist, Insectivora.

[19] Zaitsev M.V., Voyta L.L., Sheftel B.I. (2014). The Mammals of Russia and Adjacent Territories. Lipotyphlans.

[20] Voyta L.L., Abramov A.V., Lavrenchenko L.A., Nicolas V., Petrova E.A., Kryuchkova L.Y. (2022). Dental polymorphisms in *Crocidura* (Soricomorpha: Soricidae) and evolutionary diversification of crocidurine shrew dentition. Zool. J. Linn. Soc..

[21] Voet I., Denys C., Colyn M., Lalis A., Konečny A., Dlapré A., Nicolas V., Cornette R. (2022). Incongruences between morphology and molecular phylogeny provide an insight into the diversifcation of the *Crocidura poensis* species complex. Sci. Rep..

[22] Meegaskumbura S., Schneider C.J. (2008). A taxonomic evaluation of the shrew *Suncus montanus* (Soricidae: Crocidurinae) of Sri Lanka and India. Ceylon J. Sci. (Biol. Sci.).

[23] Bannikova A.A., Jenkins P.D., Solovyeva E.N., Pavlova S.V., Demidova T.B., Simanovsky S.A., Sheftel B.I., Lebedev V.S., Fang Y., Dalen L. (2019). Who are you, *Griselda*? A replacement name for a new genus of the Asiatic short-tailed shrews (Mammalia, Eulipotyphla, Soricidae): Molecular and morphological analyses with the discussion of tribal affinities. ZooKeys.

[24] Faulwetter S., Vasileiadou A., Kouratoras M., Dailianis T., Arvanitidis C. (2013). Micro-computed tomography: Introducing new dimensions to taxonomy. ZooKeys.

[25] Faulwetter S., Dailianis T., Vasileiadou K., Kouratoras M., Arvanitidis C. (2014). Can micro-CT become an essential tool for the 21st century taxonomist? An evaluation using marine polychaetes. Microsc. Anal..

[26] Winterton S.L. (2009). Revision of the stiletto fly genus *Neodialineura* Mann (Diptera: Therevidae): An empirical example of cybertaxonomy. Zootaxa.

[27] Rajmohana K., Bijoy C., Kumar A.B., Nayar M.P., Varma R.V., Peethambaran C.K. (2012). Cybertaxonomy: A novel tool in Biodiversity Science. Biodiversity: Utilization, Threats and Cultural Linkages.

[28] Smith V.S., Shorley D., Jubb M. (2013). Cybertaxonomy. The Future of Scholarly Communication.

[29] Voyta L.L., Omelko V.E., Tiunov M.P., Vinokurova M.A. (2021). When beremendiin shrews disappeared in East Asia, or how we can estimate fossil redeposition. Hist. Biol..

[30] Voyta L.L., Omelko V.E., Tiunov M.P., Petrova E.A., Kryuchkova L.Y. (2022). Temporal variation in soricid dentition: Which are first—Qualitative or quantitative features?. Hist. Biol..

[31] Voyta L.L., Omelko V.E., Izvarin E.P., Kropacheva Y.E., Eidinova E.O., Shemyakina Y.A., Nikiforova V.S., Strukova T.V., Smirnov N.G. (2023). Late Quaternary communities of shrews, Soricidae, from Ural and Far East Regions of Russia: A protocol for the multifactorial morphospace building. Proc. Zool. Inst. RAS.

[32] Voyta L.L., Izvarin E.P., Shemyakina Y.A., Nikiforova V.S., Strukova T.V., Smirnov N.G., Melnikov D.A., Bobretsov A.V. (2023). Morphospace dynamics and intraspecies variety of *Sorex araneus* and *S. tundrensis* according to recent and fossil data. Palaeontol. Electron..

[33] Polly P.D. (2023). Extinction and morphospace occupation: A critical review. Camb. Prism. Extinction.

[34] Adams D.C. (2014). A generalized K statistic for estimating phylogenetic signal from shape and other high-dimensional multivariate data. Syst. Biol..

[35] Terray L., Denys C., Goodman S.M., Soarimalala V., Lalis A., Cornette R. (2022). Skull morphological evolution in Malagasy endemic Nesomyinae rodents. PLoS ONE.

[36] Wills M., Briggs D.E.G., Fortey R.A. (1994). Disparity as an evolutionary index: A comparison of Cambrian and Recent arthropods. Paleobiology.

[37] Bannikova A.A., Yuzefovich A.P., Stefen C., Lebedev V.S., Abramov A.V. (2023). Genetic variability in the *Crocidura kegoensis*–*C. zaitsevi* group (Mammalia, Eulipotyphla) and re-evaluation of *C. zaitsevi* fromVietnam. Mamm. Biol..

[38] Hammer Ø., Harper D.A.T., Ryan P.D. (2001). PAST: Paleontological statistics soft-ware package for and data analysis. Palaeontol. Electron..

[39] Rohlf F.J. (2007). *TpsDig2*, Version 2.31; Sbmorphometrics. https://www.sbmorphometrics.org/soft-dataacq.html.

[40] Bookstein F.L. (1991). Morphometric Tools for Landmark Data: Geometry and Biology.

[41] Fedorov A., Beichel R., Kalpathy-Cramer J., Finet J., Fillion-Robin J.-C., Pujol S., Bauer C., Jennings D., Fennessy F.M., Sonka M. (2012). 3D Slicer as an Image Computing Platform for the Quantitative Imaging Network. Magn. Reson. Imaging.

[42] Polly P.D. (2003). Paleophylogeography of *Sorex araneus* (Insectivora, Soricidae): Molar shape as a morphological marker for fossil shrews. Mammalia.

[43] Rolfe S., Pieper S., Porto A., Diamond K., Winchester J., Shan S., Kirveslahti H., Boyer D., Summers A., Maga A.M. (2021). SlicerMorph: An open and extensible platform to retrieve, visualize and analyse 3D morphology. Methods Ecol. Evol..

[44] Schlager S., Zheng G., Li S., Szekely G. (2017). Morpho and Rvcg—Shape Analysis in R: R-packages for geometric morphometrics, shape analysis and surface manipulations. Statistical Shape and Deformation Analysis.

[45] Adler D., Murdoch D. (2023). *Package ‘rgl’*, Version 1.2.8; CRAN. https://cran.r-project.org/web/packages/rgl/rgl.pdf.

[46] Claude J. (2008). Morphometrics with R.

[47] Jackson D.A. (1993). Stopping rules in principal components analysis: A comparison of heuristical and statistical approaches. Ecology.

[48] Voyta L.L., Petrova T.V., Panitsina V.A., Bodrov S.Y., Abramson N.I. (2024). Complete mitochondrial genomes of Asian endemic white-toothed shrews: *Crocidura armenica* and *C. serezkyensis* (Eulipotyphla: Soricidae). R.J.T..

[49] Dubey S., Zaitsev M., Cosson J.-F., Abdukadier A., Vogel P. (2006). Pliocene and Pleistocene diversification and multiple refugia in a Eurasian shrew (Crocidura suaveolens group). Mol. Phylogenet. Evol..

[50] Ohdachi S.D., Iwasa M.A., Nesterenko V.A., Abe H., Masuda R., Haberl W. (2004). Molecular phylogenetics of Crocidura shrews (Insectivora) in east and central Asia. J. Mammal..

[51] Dubey S., Cosson J.-F., Vohralĺk V., Kryštufek B., Deker E., Vogel P. (2007). Molecular evidence of Pleistocene bidirectional faunal exchange between Europe and the Near East: The case of the bicoloured shrew (*Crocidura leucodon*, Soricidae). J. Evol. Biol..

[52] Bannikova A.A., Abramov A.V., Borisenko A.V., Lebedev V.S., Rozhnov V.V. (2011). Mitochondrial diversity of the white-toothed shrews (Mammalia, Eulipotyphla, Crocidura) in Vietnam. Zootaxa.

[53] Bannikova A.A., Lebedev V.S., Kramerov D.A., Zaitsev M.V. (2006). Phylogeny and systematics of Crocidura suaveolens species group: Corroboration and controversy between nuclear and mitochondrial DNA markers. Mammalia.

[54] Dubey S., Nová P., Vogel P., Vohralĺk V. (2007). Cytogenetic and molecular relationships between zarudny’s rock shrew, *Crocidura zarudnyi* (Mammalia: Soricomorpha) and Eurasian taxa. J. Mammal..

[55] Ohdachi S.D., Hasegawa M., Iwasa M.A., Vogel P., Oshida T., Lin L.-K., Abe H. (2006). Molecular phylogenetics of soricid shrews (Mammalia) based on mitochondrial cytochrome b gene sequences: With special reference to the Soricinae. J. Zool..

[56] Thompson J.D., Higgins D.G., Gibson T.J. (1994). CLUSTAL W: Improving the sensitivity of progressive multiple sequence alignment through sequence weighting, position-specific gap penalties and weight matrix choice. Nucleic Acids Res..

[57] Hall T.A. (1999). BioEdit: A User-Friendly Biological Sequence Alignment Editor and Analysis Program for Windows 95/98/NT. Nucleic Acids Symp. Ser..

[58] Kumar S., Stecher G., Tamura K. (2016). MEGA7: Molecular Evolutionary Genetics Analysis Version 7.0 for Bigger Datasets. Mol. Biol. Evol..

[59] Ronquist F., Teslenko M., van der Mark P., Ayres D.L., Darling A., Höhna S., Larget B., Liu L., Suchard M.A., Huelsenbeck J.P. (2012). MrBayes 3.2: Efficient Bayesian phylogenetic inference and model choice across a large model space. Syst. Biol..

[60] Rambaut A., Drummond A.J., XIe D., Baele G., Suchard M.A. (2018). Posterior Summarization in Bayesian Phylogenetics Using Tracer 1.7. Syst. Biol..

[61] Reumer J.W.F. (1984). Ruscinian and Early Pleistocene Soricidae (Insectivora, Mammalia) from Tegelen (The Netherlands) and Hungary. Scr. Geol..

[62] Dannelid E., Wójcik J.M., Wolsan M. (1998). Dental adaptations in shrew. Evolution of Shrews.

[63] Lopatin A.V. (2006). Early Paleogene insectivore mammals of Asia and establishment of the major group of Insectivora. Paleontol. J..

[64] Wible J.R. (2008). On the cranial osteology of the hispaniolan solenodon, *Solenodon paradoxus* Brandt, 1833 (Mammalia, Lipotyphla, Solenodontidae). Ann. Carnegie Mus..

[65] Maier W., Tröscher A., Ruf I. (2022). The orbitotemporal region and the mandibular joint in the skull of shrews (Soricidae, Mammalia). Vertebr. Zool..

[66] Voyta L.L., Zazhigin V.S., Petrova E.A., Krjutchkova L.Y. (2020). Shrew dentition (Lipotyphla: Soricidae)—Endodontic morphology and its phylogenetic resolving power. Mammal Res..

[67] Simpson G.G. (1940). Types in modern taxonomy. Am. J. Sci..

[68] Baranova G.I., Gureev A.A., Strelkov P.P. (1981). Type Specimens Catalogue of Collections of the Zoological Institute of USSR Academy of Sciences. Mammals (Mammalia). Insectivores (Insectivora), Bats (Chiroptera), Lagomorphs (Lagomorpha).

[69] Rich T.R., Flannery T.F., Trusler P., Kool L., van Klaveren N.A., Vickers-Rich P. (2001). A second tribosphenic mammal from the Mesozoic of Australia. Rec. Queen Vic. Mus. Launceston.

[70] Zazhigin V.S., Voyta L.L. (2022). New Neogene anourosoricin shrews from northern Asia. Palaeontol. Electron..

[71] Haring E., Voyta L.L., Däubl B., Tiunov M.P. (2015). Comparison of genetic and morphological characters in fossil teeth of grey voles from the Russian Far East (Rodentia: Cricetidae: *Alexandromys*). Mamm. Biol..

[72] Abramson N.I., Petrova T.V. (2018). Genetic analysis of type material of the Amur lemming resolves nomenclature issues and creates challenges for the taxonomy of true lemmings (*Lemmus*, Rodentia: Cricetidae) in the eastern Palearctic. Zool. J. Linn. Soc..

[73] Abramson N.I., Bodrov S.Y., Bondareva O.V., Genelt-Yanovskiy E.A., Petrova T.V. (2021). A mitochondrial genome phylogeny of voles and lemmings (Rodentia: Arvicolinae): Evolutionary and taxonomic implications. PLoS ONE.

[74] Grafodatsky A.S., Radzhabli S.I., Sharshov A.V., Zaitsev M.V. (1988). Karyotypes of five *Crocidura* species of the USSR fauna. Citology.

[75] Gureev A.A., Sokolov I.I. (1963). Insectivora—Insectivores. Mammals Fauns of USSR. Part 1.

[76] Spitzenberger F. (1971). Eine neue, tiergeographish bemerkenswerte *Crocidura* (Insectivora, Mammalia) aus der Türkei. Ann. Des Naturhistorischen Mus. Wien.

[77] Ognev S.I. (1928). 1928. Zveri Vostochnoi Evropy i Severnoi Azii. Tom I. Nasekomoyadnye i Letuchie Myshi. (The Mammals of the Eastern Europe and the Northern Asia. Vol. I. Insectivora and Chiroptera).

[78] Kuhn T.S. (1970). The Structure of Scientific Revolutions.

[79] Kuhn T.S., Suppe F. (1977). Second thoughts on paradigms. The Structure of Scientific Theories.

[80] Rychlik L., Ramalhinho G., Polly P.D. (2006). Response to environmental factors and competition: Skull, mandible and tooth shapes in Polish water shrews (*Neomys*, *Soricidae*, *Mammalia*). J. Zoolog. Syst..

[81] Cheverud J.M., Bock G., Goode J. (2007). The relationship between development and evolution through heritable variation. Tinkering: The Microevolution of Development: Novartis Foundation Symposium 285.

[82] Sanger T.J. (2006). Tinkering: A metaphor uniting evolutionary and developmental biology. Bioessays.

[83] Hallgrimsson B., Lieberman D.E., Young N.M., Parsons T., Wat S., Bock G., Goode J. (2007). Evolution of covariance in the mammalian skull. Tinkering: The Microevolution of Development: Novartis Foundation Symposium 285.

[84] Tamagnini D., Meloro C., Raia P., Maiorano L. (2021). Testing the occurrence of convergence in the cranio-mandibular shape evolution of living carnivorans. Evolution.

[85] Vasiliev A.G., Vasilieva I.A., Kourova T.P., Chibiryak M.V. (2022). An isolated population of bicolored white-toothed shrew on the northern border of its distribution range in the Orenburg region. Fauna Ural Sib..

[86] Foote M. (1991). Morphologic patterns of diversification: Examples from trilobites. Palaeontology.

[87] Churchfield S., Merritt J.F., Kirkland G.L., Rose R.K. (1994). Foraging strategies of shrews, and the evidence from field studies. Advances in the Biology of Shrews.

[88] Hanski I., Merritt J.F., Kirkland G.L., Rose R.K. (1994). Population biological consequences of body size in *Sorex*. Advances in the Biology of Shrews.

[89] Cornette R., Tresset A., Houssin C., Pascal M., Herrel A. (2015). Does bite force provide a competitive advantage in shrews? The case of the greater white-toothed shrew. Biol. J. Linn. Soc..

[90] Jernvall J., Keränen S.V.E., Thesleff I. (2000). Evolutionary modification of development in mammalian teeth: Quantifying gene expression patterns and topography. Proc. Natl. Acad. Sci. USA.

[91] Cai J., Cho S.-W., Kim J.-Y., Lee M.-J., Cha Y.-G., Jung H.-S. (2007). Patterning the size and number of tooth and its cusps. Dev. Biol..

[92] Kavanagh K.D., Evans A.R., Jernvall J. (2007). Predicting evolutionary patterns of mammalian teeth from development. Nature.

[93] Salazar-Ciudad I. (2008). Tooth morphogenesis *in vivo*, *in vitro*, and *in silico*. Curr. Top Dev. Biol..

[94] Drake A.G. (2011). Dispelling dog dogma: An investigation of heterochrony in dogs using 3D geometric morphometric analysis of skull shape. Evol. Dev..

[95] Prost S., Klietmann J., van Kolfschoten T., Guralnick R.P., Waltari E., Vrieling K., Stiller M., Nagel D., Rabeder G., Hofreiter M. (2013). Effects of Late Quaternary climate change on Palearctic shrews. Glob. Chang. Biol..

[96] Chen S., Qing J., Liu Z., Liu Y., Tang M., Murphy R.W., Pu Y., Wang X., Tang K., Guo K. (2020). Multilocus phylogeny and cryptic diversity of white-toothed shrews (Mammalia, Eulipotyphla, *Crocidura*) in China. BMC Evol. Biol..

[97] Arnaudo M.E., Arnal M., Ekdale E.G. (2020). The auditory region of a caviomorph rodent (Hystricognathi) from the early Miocene of Patagonia (South America) and evolutionary considerations. J. Vertebr. Paleontol..

[98] Skandalos P., van den Hoek Ostende L.W. (2023). Wear-dependent molar morphology in hypsodont rodents: The case of the spalacine *Pliospalax*. Palaeontol. Electron..

