# Supplementary material for: A Cybertaxonomic Revision of the “Crocidura pergrisea” Species Complex with a Special Focus on Endemic Rocky Shrews: Crocidura armenica and Crocidura arispa (Soricidae)
Source: Biology (Basel). 2024 Jun 18;13(6):448. doi: 10.3390/biology13060448 (PMC11200727; doi:10.3390/biology13060448)

Supplementary Materials

Table S1. List of three-dimensional models used in the study and micro-CT scanning technical properties. NEOSCAN N80 (CCD) and SkyScan 1172 (CCD) scanners were a part of the facilities of the Resource Centre for X-ray Diffraction Studies of Saint Petersburg State University (Saint Petersburg, Russia); YXLON FF35 CT (XRD 4343CT) is a part of the facilities of the Natural History Museum Vienna (Vienna, Austria).
Key: **(m)**, used only hemimandible model; **(s)**, used only skull model. Models are available in MorphoBank, Projects: **3885** (http://dx.doi.org/10.7934/P3885) and **4964** (https://morphobank.org/index.php/Projects/ProjectOverview/project_id/4964).

nn	Species	Abbr.	Coll. Number	Resolution, µm (original/working)	micro-CT Scanner
1	*C. armenica*	arm	ZIN 45277	4.5/9.00	NEOSCAN N80 (CCD)
2	*C. armenica*	arm-t	ZIN 55321	4.0/8.0	NEOSCAN N80 (CCD)
3	*C. armenica*	arm_972	ZIN 77972	3.0/6.0	NEOSCAN N80 (CCD)
4	*C. armenica*	arm_973	ZIN 77973	4.2/8.4	NEOSCAN N80 (CCD)
5	*C. armenica*	arm_976	ZIN 77976	4.2/8.4	NEOSCAN N80 (CCD)
6	*C. arispa*	ars	NHMW 13284	5.4/10.8^{mandible} 4.30/8.60^{skull}	YXLON FF35 CT (XRD 4343CT)
7	*C. serezkyensis*	szr	ZIN 77431	4.14/8.28	SkyScan 1172 (CCD)
8	*C. caspica*	csp_763	ZIN 72763	4.5/9.0	NEOSCAN N80 (CCD)
9	*C. caspica*	csp_764	ZIN 72764	4.6/9.2	NEOSCAN N80 (CCD)
10	*C. lasiura*	las_015	ZIN 76015	4.41/8.82	SkyScan 1172 (CCD)
11	*C. lasiura*	las_017	ZIN 76017	4.41/8.82	SkyScan 1172 (CCD)
12	*C. lasiura* (s)	las_019	ZIN 76019	6.89/6.89	SkyScan 1172 (CCD)
13	*C. lasiura* (m)	las_020	ZIN 76020	4.07/8.14	SkyScan 1172 (CCD)
14	*C. lasiura* (m)	las_021	ZIN 76021	4.07/8.14	SkyScan 1172 (CCD)
15	*C. lasiura* (m)	KRD	FSC RJARV-KorC-93	3.98/7.96	SkyScan 1172 (CCD)
16	*C. leucodon*	lcd_918	ZIN 72918	6.0/6.0	SkyScan 1172 (CCD)
17	*C. leucodon*	lcd_921	ZIN 72921	4.0/8.0	NEOSCAN N80 (CCD)
18	*C. gueldenstaedtii*	gld_843	ZIN 72843	4.5/9.0	NEOSCAN N80 (CCD)
19	*C. gueldenstaedtii*	gld_872	ZIN 72872	4.0/8.0	NEOSCAN N80 (CCD)
20	*C. phanluongi*	phg	ZIN 100307	4.48/8.96	SkyScan 1172 (CCD)
21	*C. sapaensis*	sps	ZIN 96265	4.48/8.96	SkyScan 1172 (CCD)
22	*C. shantungensis*	sha-427	ZIN 89427	4.58/9.16	SkyScan 1172 (CCD)
23	*C. shantungensis*	sha-433	ZIN 89433	4.58/9.16	SkyScan 1172 (CCD)
24	*C. shantungensis*	sha-435	ZIN 89435	4.58/9.16	SkyScan 1172 (CCD)
25	*C. shantungensis*	sha-445	ZIN 89445	4.58/9.16	SkyScan 1172 (CCD)
26	*C. shantungensis* (s)	sha-469	ZIN 89469	4.58/9.16	SkyScan 1172 (CCD)
27	*C. sibirica*	sib_423	ZIN 79423	4.58/9.16	SkyScan 1172 (CCD)
28	*C. sibirica*	sib_431	ZIN 79431	4.58/9.16	SkyScan 1172 (CCD)
29	*C. sibirica*	sib_437	ZIN 79437	4.58/9.16	SkyScan 1172 (CCD)
30	*C. sibirica*	sib_439	ZIN 79439	4.58/9.16	SkyScan 1172 (CCD)
31	*C. suaveolens*	sua_665	ZIN 73665	4.58/9.16	SkyScan 1172 (CCD)
32	*C. suaveolens*	sua_756	ZIN 73756	4.58/9.16	SkyScan 1172 (CCD)
33	*C. suaveolens*	sua_220	ZIN 77220	4.58/9.16	SkyScan 1172 (CCD)
34	*C. suaveolens*	sua_863	ZIN 98863	4.58/9.16	SkyScan 1172 (CCD)
35	*C. suaveolens*	sua_864	ZIN 98864	4.58/9.16	SkyScan 1172 (CCD)
36	*C.* ex gr. *kegoensis-zaitsevi*	zts	ZIN 96320	6.89	SkyScan 1172 (CCD)
37	*C. zarudnyi*	zrd	ZIN 6506	4.14	SkyScan 1172 (CCD)
38	*Suncus murinus*	mrn	ZIN 15885	4.48/8.96, 13.44^{muz}	SkyScan 1172 (CCD)
39	*Sorex minutissimus*	mss	ZIN 98582	6.48	SkyScan 1172 (CCD)

Table S2. List of specimens, used in the study. 3D models specimens are bolded; *, localities see in Table S3.

1. *Crocidura armenica* Gureev, 1963: **ZIN 45277** (holotype), **ZIN 55321** (paratype), **ZIN 77972, 77973, 77976** (n = 5);
2. *Crocidura arispa* Spitzenberger, 1971: **NHMW 13284** (holotype) (n = 1);
3. *Crocidura caspica* Thomas, 1907: **ZIN 72763, 72764** (n = 2);
4. *Crocidura lasiura* Dobson, 1890: Gaivoron*: ZIN 76007, **76015, 76017, 76019-76021**, 76023, 76025, 76808, 76809, 76810 (n = 11); Khasan: ZIN 89320, 89335, 89336, 89339 89351, 89356 (n = 6); KorC: **FSC RJARV-KorC-93** (n = 1);
5. *Crocidura leucodon* (Hermann, 1780): Haskovo: ZIN 43861 (n = 1); Yambol: ZIN 43865 (n = 1); Sashaburo: ZIN 72910, 72914, 72916, 72917, **72918**, 72919, **72921**-72923, 72981 (n = 10);
6. *Crocidura gueldenstaedtii* (Pallas, 1811): Garni: ZIN 69541, 69543, 69544, 69546, 69547, 69549, 69553-69555, 69557, 69559, 69561, 69563 (n = 13); Sarysu: ZIN 72834, 72838, 72839, 72841, 72842, **72843**, 72844, 72845, 72847, 72848, **72852**, 72855-72859 (n = 16); Julfa: ZIN 72872 (n = 1);
7. *Crocidura phanluongi* Jenkins, Abramov, Rozhnov & Makarova, 2010: Yok Don: ZIN 97092 (n = 1); Ba Hao: ZIN 99336, 99338, 100306, **100307** (n = 1);
8. *Crocidura sapaensis* Jenkins, Abramov, Bannikova & Rozhnov, 2013: Sa Pa: ZIN 96262, 96264, **96265**, 96271, 96274, 96275, 96276, 96432, 96434, 96438, 96442 (n = 11);
9. *Crocidura serezkyensis* Laptev, 1929: **ZIN 77431** (n = 1);
10. *Crocidura shantungensis* Miller, 1901: Khasan: ZIN **89427, 89433, 89435, 89445**-89454, 89465-89468, **89469**, 89470-89474 (n = 23);
11. *Crocidura sibirica* Dukelsky, 1930: Kozhevnikovo: ZIN 43476, 43478 (n = 2); Artybash: ZIN 73194, 73240 (n = 2); Azhendarovo: ZIN 79406-79408 (n = 3); Saltymakovo: ZIN 79412, 79414, 79415, 79417-79419, 79421- **79423**, **79431**, 79430-79436, **79437**, 79438, **79439**, 79440-79444 (n = 23);
12. *Crocidura suaveolens* (Pallas, 1811): Prokhladny: ZIN 72776, 72777, 72780, 72787, 72789, 72793, 72795, 72796, 72804 (n = 9); Anapa: ZIN 73453, 73454, 73457, 73458, 73460, 73463-73468, 73470, 73472, 73474, 73475 (n = 15); Badkhyz: **ZIN 73665, 73756** (n = 2); Iskanderkhul: **ZIN 77220, 98863, 98864** (n = 3);
13. *Crocidura zaitsevi* Jenkins, Abramov, Rozhnov and Makarova, 2007: Ngoc Linh: ZIN 91215-91219, 91223, 91224 (n = 7); *C.* ex gr. *kegoensis-zaitsevi*: Ngoc Linh**: ZIN 96320** (n = 1);
14. *Crocidura zarudnyi* Ognev, 1928: Jamalabad: **ZIN 6506** (n = 1);
15. *Suncus murinus* (Linnaeus, 1766): Ryukyu: **ZIN 15885** (n = 1);
16. *Sorex minutissimus* Zimmermann, 1780: Novikovo: **ZIN 98582** (n = 1).

Table S3. List of localities. **Abbr**., acronyms of 3D models.

Species	Abbr.	Locality
Crocidura armenica, type locality	arm (holotype ZIN 45277)	1. Garni — Garni Village vicinity, Kotayk Province, Armenia; N 40.119444, E 44.723056; ca. 1400 m a.s.l. (rocks, stone debris).
C. armenica, paratype	arm-t (paratype ZIN 55321)	2. Sisevan — Sisian [Sisevan] Town vicinity, Syunik Province, Armenia; N 39.516667, E. 46.016667; 1600-2000 m a.s.l.
C. armenica, new hypodigm	arm_972, arm_973, arm_976	3. Julfa — Julfa City vicinity, Nakhchivan Autonomous Republic of Azerbaijan; N 38.987778, E 45.576667; 700-1200 m a.s.l. (rocks, stone debris).
C. arispa, type locality	ars (holotype NHMW 13284)	4. Niğde — South Madenköy Mts (Taurus Mts), Ulukışla District, Niğde Province, Turkey; N 37.457222, E 34.618889; ca. 2000 a.s.l.
C. caspica, n = 2	csp_763, csp_764	5. Lerik — Lerik District, Azerbaijan; N 38.783333, E 48.416667; ? m.
C. lasiura, n = 11, Recent	las_015, las_017, las_019-021	6. Gaivoron — Gaivoron Village [Gaivoronskii], Spasskii Disrtrict, Primorsky Krai, Russia; N 44.747778, E 132.773056; 70-100 m a.s.l.
C. lasiura, n = 6, Recent		7. Khasan — Khasan Village vicinity, Khasansky District, Primorsky Krai, Russia; N 42.428333, E 130.645556; before 100 m a.s.l.
C. lasiura, n = 1, Late Pleistocene	KRD	8. Koridornaya Cave locality — Pompeevskii Ridge (Lesser Khingan Ridge spurs) and the right bank of the Stolbukha River; Jewish Autonomous Oblast, Far East, Russia; ca. N 48.00, E 130.98333.
C. leucodon, n = 1		9. Haskovo — Haskovo City vicinity, Haskovo Province, Northern Thrace Region, Bulgaria; N 41.933333, E. 25.566667; ca. 200 m a.s.l.
C. leucodon, n = 1		10. Yambol — Yambol City vicinity, Yambol Province, Bulgaria; N 42.483333, E 26.5; ca. 100 m a.s.l.
C. leucodon, n = 10	lcd_918, lcd_921	11. Sashaburo — Sashaburo, Dusheti Municipality, Mtskheta-Mtianeti Region, Georgia; N 42.03367, E 44.70101; ca. 1000 m a.s.l.
C. gueldenstaedtii, n = 13		12. Garni — Garni Village vicinity, Kotayk Province, Armenia; N 40.119444, E 44.723056; ca. 1400 m a.s.l. (reed, wet habitats).
C. gueldenstaedtii, n = 16	gld_843	13. Sarysu — Sarysu Lake, Kur-Araz Lowland, Azerbaijan; N 40.047222, E 48.172778; ca. 10 m a.s.l.
C. gueldenstaedtii, n = 1	gld_872	14. Julfa — Julfa City vicinity, Nakhchivan Autonomous Republic of Azerbaijan; N 38.987778, E 45.576667; 700-1200 m a.s.l. (reed, wet habitats).
C. phanluongi, type locality, holotype		15. Yok Don — Yok Don Nat. Park (northern part), Dak Lak Province, Vietnam; N 12.966667, E 107.816667; ca. 250 m a.s.l.
C. phanluongi, n = 4	phg (paratype ZIN 100307)	16. Ba Hao — Ba Hao vicinity, Vinh Cuu Nat. Reserve (= Ma Da Forest), Vinh Cuu District, Dong Nai Province, Vietnam; N 11.309444, E 107.078611; ca. 80 m a.s.l.

C. sapaensis, n = 11	sps	17. Sa Pa — Sa Pa Village Vicinity, North slope of Phansipan Mt., Tram Ton station of Hoang Lien National Park, Lao Cai Province, Vietnam; N 22.3500000, E 103.7666667; ? m.
C. serezkyensis, n = 1	szr	18. Sarez — Irkht Bay of Sarez Lake, Rushon District, Gorno-Badakhshan Province, Tajikistan; N 38.183333, E 72.633333; ca. 3200 m a.s.l.
C. shantungensis, n = 23	sha_427, sha_433, sha_435, sha_445, sha_469	Khasan — Khasan Village vicinity, Khasansky District, Primorsky Krai, Russia; N 42.428333, E 130.645556; before 100 m a.s.l.
C. sibirica, n = 2		19. Kozhevnikovo — Kozhevnikovo Village vicinity, Kozhevnikovo District, Tomsk Oblast, Russia; N 56.25, E 83.966667; ca. 90 m a.s.l.
C. sibirica, n = 2		20. Artybash — Artybash Village vicinity, Turochaksky District, Altai Republic, Russia; N 51.791111, E 87.255833; ca. 400 m a.s.l.
C. sibirica, n = 3		21. Azhendarovo — Azhendarovo Village vicinity (at present not existed), Krapivinsky District, Kemerovo Oblast, Russia; N 54.756111, E 87.022778; ca. 200 m a.s.l.
C. sibirica, n = 23	sib_423, sib_431, sib_437, sib_439	22. Saltymakovo — Saltymakovo Village vicinity, Krapivinsky District, Kemerovo Oblast, Russia; N 54.823889, E 87.070278; ca. 150 m a.s.l.
C. suaveolens, n = 9		23. Prokhladny — Prokhladny Town vicinity, Prokhladnensky District, Kabardino-Balkar Republic, Russia; N 43.75, E 44.033333; ca. 200 m a.s.l.
C. suaveolens, n = 15		24. Anapa — Anapa Town vicinity, Krasnodar Krai, Russia; N 44.893889, E 37.3175; ca. 15 m a.s.l.
C. suaveolens, n = 2	sua_665, sua_756	25. Badkhyz — Badhyz State Nature Reserve, south-western Turkmenistan; ?
C. suaveolens, n = 3	sua_220, sua_863, sua_864	26. Iskanderkhul — Sarytag River mouth, Iskanderkul Lake, Sughd Province, Tajikistan; N 39.063119, E 68.354994; ca. 2200 m a.s.l.
C. zaitsevi, type series, n = 7		27. Ngoc Linh — Ngoc Linh Mountain (west slope), Central Highlands, Kon Tum Province, Vietnam; N 15.083333, E 107.95; ca. 2300 m a.s.l.
C. kegoensis-zaitsevi, n = 1	zts	Ngoc Linh — Ngoc Linh Mountain (west slope), Central Highlands, Kon Tum Province, Vietnam; N 15.083333, E 107.95; ca. 2300 m a.s.l.
C. zarudnyi, type locality	zrd (ZIN 6506)	28. Jamalabad — Jamalabad Village vicinity, 2 km along a stream (azimuth 121.09), Central District of Zabol County, Sistan and Baluchestan province, Iran; N 30.910119, E 61.377353; ca. 500 m a.s.l. [*Zarudny NA, 1916. Third excursion through East Persia (Khorosan, Seistan and Persian Baluchestan). 1900-1901. Notes of the Imperial Russian Geographical Society on General Geography* ***50****:1–448.*]
Suncus murinus, n = 1	mrn	29. Ryukyu — Ryukyu Islands, Okinawa Prefecture, Japan; ca. N 26.5, E 128.0; ? m.
Sorex minutissimus, n = 1	mss	30. Novikovo — Novikovo Village vicinity, Korsakovsky District, Sakhalin Oblast, Russia; N 46.3636, E 143.3594; ca. 10 m a.s.l.

Table S4. Univariate statistics of nine linear dimensions of the *C. gueldenstaedtii* sample (n = 30) and values of 3D model specimens. **Std. error**, standard error of mean; **Stand. dev**, standard deviation. Unfilled part represents univariate description of the sample; filled part shows a particular values of specimens that used in the 3D analysis (see Table S1).

	MBH	MRH	LML	CIL	PL	UML	ZYG	P4s/d	EGW
N	30	30	30	25	27	27	27	27	27
Min	0.97	3.68	3.34	15.39	6.27	2.74	4.79	0.86	5.07
Max	1.39	4.89	4.25	19.52	8.39	3.54	6.3	1.42	6.57
Mean	1.19	4.40	3.93	17.95	7.66	3.27	5.64	1.17	5.95
Std. error	0.02	0.05	0.03	0.21	0.10	0.04	0.06	0.02	0.07
Stand. dev	0.11	0.30	0.21	1.08	0.52	0.21	0.36	0.11	0.40
ZIN 72843	1.29	4.65	4.1	19.52	8.27	3.5	6.3	1.23	6.57
ZIN 72872	1.39	4.56	3.98	18.05	7.44	3.29	5.54	1.21	5.82

Table S5. Univariate statistics of nine linear dimensions of the *C. lasiura* sample (*n* = 14) and values of 3D model specimens, including fossil KRD (FSC RJARV-KorC-93). Description of a coloration see in Table S4.

	MBH	MRH	LML	CIL	PL	UML	ZYG	P4s/d	EGW
N	14	17	17	17	17	17	16	17	17
Min	1.61	5	4.45	19.89	8.24	3.52	6.66	1.25	6.22
Max	2.16	7.01	4.77	24.48	10.48	4.09	7.77	1.61	7.51
Mean	1.80	5.68	4.61	21.38	9.10	3.84	7.02	1.41	6.63
Std. error	0.05	0.12	0.03	0.36	0.15	0.03	0.08	0.02	0.08
Stand. dev	0.19	0.53	0.12	1.49	0.63	0.15	0.33	0.11	0.34
ZIN 76015	1,75	5,6	4,45	19,98	8,37	3,76	6,95	1,35	6,52
ZIN 76017	1,73	5,6	4,73	20,85	8,84	3,8	6,85	1,25	6,9
ZIN 76019	1,87	5,62	4,55	20,12	8,53	3,52	6,84	1,49	6,5
ZIN 76020	1,64	5,35	4,53	20,4	8,76	3,82	n.a.	1,32	6,41
ZIN 76021	1,75	5,6	4,45	19,98	8,37	3,76	6,95	1,35	6,52
KRD	1,79	5,31	4,4	n.a.	n.a.	n.a.	n.a.	n.a.	n.a.

Table S6. Univariate statistics of nine linear dimensions of the *C. leucodon* sample (n = 12) and values of 3D model specimens. Description of a coloration see in Table S4.

	MBH	MRH	LML	CIL	PL	UML	ZYG	P4s/d	EGW
N	12	12	12	12	12	12	12	12	12
Min	1.22	4.42	3.8	17.42	7.33	3.08	5.87	1.04	6.07
Max	1.5	4.97	4.38	19.24	8.32	3.49	6.53	1.22	6.74
Mean	1.37	4.72	4.06	18.40	7.88	3.31	6.17	1.13	6.35
Std. error	0.02	0.05	0.04	0.18	0.08	0.03	0.06	0.01	0.05
Stand. dev	0.09	0.17	0.16	0.65	0.29	0.13	0.22	0.06	0.20
ZIN 72918	1.35	4.94	4.38	18.8	8.11	3.49	6.29	1.21	6.42
ZIN 72921	1.32	4.61	4.06	19.1	8.03	3.42	6.13	1.08	6.34

Table S7. Univariate statistics of nine linear dimensions of the *C. sapaensis* sample (n = 11) and values of 3D model specimens. Description of a coloration see in Table S4.

	MBH	MRH	LML	CIL	PL	UML	ZYG	P4s/d	EGW
N	11	11	11	11	11	11	11	11	11
Min	0.09	3.48	3.16	14.94	6.08	2.7	4.85	0.98	4.96
Max	1.2	3.96	3.49	16.52	6.83	2.91	5.34	1.18	5.6
Mean	0.94	3.75	3.33	15.90	6.51	2.80	5.07	1.09	5.25
Std. error	0.08	0.04	0.02	0.14	0.07	0.02	0.06	0.01	0.05
Stand. dev	0.29	0.14	0.08	0.49	0.24	0.07	0.20	0.05	0.19
ZIN 96265	1.2	3.74	3.41	16.27	6.56	2.86	5.34	1.11	5.33

Table S8. Univariate statistics of nine linear dimensions of the *C. shantungensis* sample (n = 23) and values of 3D model specimens. Description of a coloration see in Table S4.

	MBH	MRH	LML	CIL	PL	UML	ZYG	P4s/d	EGW
N	23	22	23	23	23	23	23	23	23
Min	0.87	3.31	3.2	14.7	6.03	2.63	4.68	0.98	4.77
Max	1.17	4.04	3.6	16.43	6.77	3.02	5.42	1.2	5.49
Mean	0.97	3.69	3.40	15.75	6.43	2.84	5.05	1.12	5.24
Std. error	0.01	0.03	0.02	0.08	0.04	0.01	0.03	0.01	0.03
Stand. dev	0.07	0.16	0.10	0.42	0.19	0.09	0.15	0.05	0.18
ZIN 89427	1.17	3.86	3.52	16.08	6.62	2.94	5.42	1.19	5.16
ZIN 89433	1.06	3.90	3.28	15.6	6.22	2.74	5.1	1.19	5.07
ZIN 89435	0.90	4.04	3.53	15.96	6.39	2.96	5.1	1.11	5.27
ZIN 89445	1.00	3.54	3.38	15.69	6.34	2.83	4.8	1.1	5.05
ZIN 89469	0.9	3.63	3.42	15.55	6.36	2.81	5.16	1.16	5.17

Table S9. Univariate statistics of nine linear dimensions of the *C. sibirica* sample (n = 30) and values of 3D model specimens. Description of a coloration see in Table S4.

	MBH	MRH	LML	CIL	PL	UML	ZYG	P4s/d	EGW
N	30	30	29	27	29	29	29	29	29
Min	1.17	4.1	3.71	16.92	7.23	3.27	5.58	1.07	5.74
Max	1.57	5.18	4.45	18.7	7.96	3.59	6.34	1.33	6.77
Mean	1.40	4.75	4.16	17.97	7.51	3.45	6.04	1.21	6.25
Std. error	0.01	0.03	0.02	0.09	0.03	0.01	0.03	0.01	0.03
Stand. dev	0.09	0.21	0.14	0.49	0.19	0.08	0.16	0.06	0.21
ZIN 79423	1.56	5	4.25	18.48	7.81	3.56	6.04	1.26	6.06
ZIN 79431	1.46	4.9	4.25	18.31	7.66	3.55	6.07	1.2	6.47
ZIN 79437	1.5	5.03	4.04	18.29	7.6	3.46	6.01	1.24	6.48
ZIN 79439	1.43	4.88	4.13	18.22	7.45	3.48	6.07	1.29	6.07

Table S10. Univariate statistics of nine linear dimensions of the *C. suaveolens* sample (n = 29) and values of 3D model specimens. Description of a coloration see in Table S4.

	MBH	MRH	LML	CIL	PL	UML	ZYG	P4s/d	EGW
N	29	29	27	28	29	29	29	28	29
Min	0.98	3.73	3.63	16.22	6.74	2.98	5.19	0.99	5.43
Max	1.25	4.58	3.98	17.78	7.72	3.28	5.7	1.31	6.02
Mean	1.13	4.21	3.78	16.99	7.18	3.15	5.46	1.17	5.71
Std. error	0.01	0.03	0.02	0.08	0.05	0.02	0.03	0.02	0.03
Stand. dev	0.07	0.18	0.09	0.40	0.25	0.09	0.14	0.08	0.18
ZIN 73665	1.15	4.58	3.67	16.6	6.91	3.05	5.45	1.07	5.68
ZIN 73756	1.24	4.34	3.74	16.78	6.98	3.11	5.44	1.16	5.58
ZIN 77220	1.18	4.23	3.83	n.a.	6.87	3.21	5.44	1.03	5.55
ZIN 98863	1.21	4.25	3.87	17.06	7.34	3.23	5.45	1.05	5.57
ZIN 98864	1.25	4.45	3.81	17.25	7.33	3.19	5.7	1.17	5.56

Table S11. Univariate statistics of nine linear dimensions of the *C. zaitsevi* sample (n = 7) and values of *C.* ex gr. *kegoensis-zaitsevi* (ZIN 96320). Description of a coloration see in Table S4.

	MBH	MRH	LML	CIL	PL	UML	ZYG	P4s/d	EGW
N	7	7	7	7	7	7	7	7	7
Min	0.83	3.33	2.99	14.45	5.98	2.47	4.45	0.81	4.41
Max	0.98	3.56	3.23	15.02	6.25	2.73	4.74	1.03	4.87
Mean	0.91	3.44	3.15	14.74	6.14	2.65	4.58	0.90	4.68
Std. error	0.02	0.03	0.03	0.07	0.03	0.03	0.05	0.03	0.05
Stand. dev	0.06	0.09	0.08	0.20	0.09	0.09	0.12	0.07	0.14
ZIN 96320	1.49	5.08	4.37	n.a.	8.18	3.57	7.02	1.35	6.56

Table S12. Homogeneity of the *C. gueldenstaedtii* sample (n = 30) estimated by set of parametric (W, JB) and non-parametric (A) normality tests, calculated using PAST (ver. 4.03; Hammer et al. 2001). A positive test result (= sample is homogenic) is light green.

	MBH	MRH	LML	CIL	PL	UML	ZYG	P4s/d	EGW
N	30	30	30	25	27	27	27	27	27
Shapiro-Wilk W	0.9779	0.8871	0.4886	0.8623	0.8936	0.8972	0.9464	0.9721	0.9148
p(normal)	0.7687	0.004122	4.07E-09	0.003047	0.009581	0.01153	0.1751	0.6587	0.02961
Anderson-Darling A	0.192	1.353	4.712	1.406	1.105	1.002	0.633	0.314	0.7756
p(normal)	0.8881	0.00135	5.60E-12	0.0009464	0.005595	0.01024	0.08857	0.5255	0.03844
p(Monte Carlo)	0.8906	0.0014	0.0001	0.0009	0.0063	0.0109	0.0835	0.5484	0.039
Jarque-Bera JB	0.8902	4.943	485.2	5.768	5.446	4.052	1.655	1.953	2.956
p(normal)	0.6408	0.08447	4.30E-106	0.05592	0.06566	0.1319	0.4371	0.3766	0.2281
p(Monte Carlo)	0.5328	0.0406	0.0001	0.0306	0.0339	0.0573	0.2314	0.1576	0.0856

Hammer, Ø., Harper, D.A.T. and Ryan, P.D. 2001. PAST: paleontological statistics soft-ware package for and data analysis. *Palaeontol. Electron.* **2001**, *4, 1–9*. Available from: http://palaeoelectronica.org/2001_1/past/issue1_01.htm (accessed 03 April 2023).

Table S13. Homogeneity of the *C. lasiura* sample (n = 14) estimated by set of parametric (W, JB) and non-parametric (A) normality tests, calculated in the PAST (ver. 4.03). See Table S12. A positive test result (= sample is homogenic) is light green.

	MBH	MRH	LML	CIL	PL	UML	ZYG	P4s/d	EGW
N	30	30	29	27	29	29	29	29	29
Shapiro-Wilk W	0.8458	0.853	0.8429	0.8356	0.8914	0.9686	0.8347	0.9253	0.8592
p(normal)	0.01949	0.01193	0.008361	0.006527	0.04885	0.7928	0.008172	0.1815	0.01487
Anderson-Darling A	0.817	1.103	1.093	1.077	0.7996	0.2731	1.103	0.5234	1.019
p(normal)	0.02573	0.00496	0.005265	0.005787	0.03034	0.621	0.004838	0.1562	0.008177
p(Monte Carlo)	0.0232	0.0058	0.0048	0.0049	0.0312	0.6528	0.0056	0.1599	0.0074
Jarque-Bera JB	1.912	4.635	2.148	3.096	2.375	0.2345	3.69	1.177	4.86
p(normal)	0.3845	0.09851	0.3417	0.2127	0.3049	0.8893	0.158	0.5552	0.08805
p(Monte Carlo)	0.1015	0.0364	0.0961	0.0589	0.0906	0.883	0.0461	0.2998	0.0323

Table S14. Homogeneity of the *C. leucodon* sample (n = 12) estimated by set of parametric (W, JB) and non-parametric (A) normality tests, calculated in the PAST (ver. 4.03). See Table S12. A positive test result (= sample is homogenic) is light green.

	MBH	MRH	LML	CIL	PL	UML	ZYG	P4s/d	EGW
N	12	12	12	12	12	12	12	12	12
Shapiro-Wilk W	0.9255	0.9382	0.9808	0.8797	0.9401	0.9174	0.9319	0.9011	0.9629
p(normal)	0.3347	0.4754	0.9868	0.08681	0.4997	0.2651	0.4009	0.1638	0.8241
Anderson-Darling A	0.4256	0.332	0.1719	0.6087	0.3458	0.4613	0.2928	0.4891	0.1996
p(normal)	0.2628	0.4535	0.907	0.08673	0.4191	0.2119	0.5421	0.1786	0.8481
p(Monte Carlo)	0.2735	0.4746	0.932	0.0916	0.4365	0.2208	0.5772	0.1848	0.874
Jarque-Bera JB	0.1865	0.1702	0.1273	0.2057	0.1651	0.2026	0.1366	0.19	0.1336
p(normal)	0.2902	0.4297	0.8518	0.1683	0.4793	0.1846	0.7699	0.2646	0.7979
p(Monte Carlo)	0.291	0.4315	0.8414	0.1696	0.4846	0.1885	0.7713	0.2745	0.8014

Table S15. Homogeneity of the *C. sapaensis* sample (n = 11) estimated by set of parametric (W, JB) and non-parametric (A) normality tests, calculated in the PAST (ver. 4.03). See Table S12. A positive test result (= sample is homogenic) is light green.

	MBH	MRH	LML	CIL	PL	UML	ZYG	P4s/d	EGW
N	11	11	11	11	11	11	11	11	11
Shapiro-Wilk W	0.5853	0.9472	0.9704	0.9367	0.9427	0.9391	0.838	0.9361	0.8952
p(normal)	1.67E-05	0.6087	0.891	0.4828	0.5525	0.5101	0.02976	0.4757	0.1614
Anderson-Darling A	1.99	0.2841	0.2583	0.3215	0.2656	0.3067	0.731	0.3525	0.5569
p(normal)	1.73E-05	0.5589	0.6421	0.4743	0.6167	0.5096	0.03955	0.397	0.116
p(Monte Carlo)	0.0001	0.6019	0.6834	0.4925	0.6672	0.5342	0.0372	0.4233	0.1239
Jarque-Bera JB	23.29	0.1696	0.07944	0.9245	0.6702	0.8892	1.413	0.4113	0.9236
p(normal)	8.77E-06	0.9187	0.9611	0.6299	0.7153	0.6411	0.4933	0.8141	0.6302
p(Monte Carlo)	0.0001	0.9257	0.9668	0.3365	0.5441	0.3632	0.1341	0.768	0.3299

Table S16. Homogeneity of the *C. shantungensis* sample (n = 23) estimated by set of parametric (W, JB) and non-parametric (A) normality tests, calculated in the PAST (ver. 4.03). See Table S12. A positive test result (= sample is homogenic) is light green.

	MBH	MRH	LML	CIL	PL	UML	ZYG	P4s/d	EGW
N	23	22	23	23	23	23	23	23	23
Shapiro-Wilk W	0.9384	0.9768	0.9615	0.9668	0.9637	0.9798	0.9588	0.9359	0.9355
p(normal)	0.166	0.8591	0.4938	0.613	0.5426	0.903	0.4402	0.1467	0.144
Anderson-Darling A	0.4531	0.3068	0.461	0.2698	0.3578	0.2067	0.4423	0.4779	0.4416
p(normal)	0.2473	0.5356	0.2362	0.6455	0.4233	0.8503	0.2632	0.2141	0.2643
p(Monte Carlo)	0.2485	0.5581	0.2442	0.6714	0.4325	0.8626	0.2753	0.2198	0.2596
Jarque-Bera JB	1.952	0.1495	0.2902	0.8116	0.4612	0.0733	1.04	2.143	1.151
p(normal)	0.3768	0.928	0.8649	0.6665	0.794	0.964	0.5946	0.3425	0.5626
p(Monte Carlo)	0.1483	0.9305	0.851	0.5412	0.7525	0.9671	0.4174	0.1323	0.3688

Table S17. Homogeneity of the *C. sibirica* sample (n = 30) estimated by set of parametric and non-parametric normality tests, calculated in the PAST (ver. 4.03). See Table S12. A positive test result (= sample is homogenic) is light green.

	MBH	MRH	LML	CIL	PL	UML	ZYG	P4s/d	EGW
N	30	30	29	27	29	29	29	29	29
Shapiro-Wilk W	0.9748	0.9499	0.9281	0.9367	0.9514	0.9511	0.9695	0.9646	0.9687
p(normal)	0.677	0.168	0.0491	0.1011	0.1991	0.1951	0.546	0.4251	0.5245
Anderson-Darling A	0.2559	0.4263	0.6669	0.6821	0.4898	0.5184	0.2585	0.3644	0.4539
p(normal)	0.7014	0.2948	0.07328	0.06643	0.2043	0.1727	0.6916	0.4149	0.2514
p(Monte Carlo)	0.7153	0.3053	0.0747	0.0693	0.2065	0.1707	0.7107	0.4214	0.2624
Jarque-Bera JB	0.2508	5.319	10.42	1.425	1.663	1.66	1.406	1.075	0.1311
p(normal)	0.8821	0.06998	0.005461	0.4905	0.4354	0.436	0.4951	0.5843	0.9366
p(Monte Carlo)	0.8728	0.0385	0.0124	0.2913	0.2238	0.2251	0.3001	0.4425	0.9343

Table S18. Homogeneity of the *C. suaveolens* sample (n = 29) estimated by set of parametric (W, JB) and non-parametric (A) normality tests, calculated in the PAST (ver. 4.03). See Table S12. A positive test result (= sample is homogenic) is light green.

	MBH	MRH	LML	CIL	PL	UML	ZYG	P4s/d	EGW
N	29	29	27	28	29	29	29	28	29
Shapiro-Wilk W	0.9818	0.9733	0.9678	0.9723	0.969	0.9347	0.9601	0.9733	0.9413
p(normal)	0.8814	0.6528	0.5453	0.6432	0.5329	0.07305	0.3305	0.6723	0.1087
Anderson-Darling A	0.2156	0.3546	0.2746	0.2413	0.3555	0.545	0.3899	0.2792	0.5483
p(normal)	0.8306	0.4376	0.6345	0.7496	0.4354	0.1474	0.3604	0.6205	0.1446
p(Monte Carlo)	0.8331	0.4491	0.656	0.7666	0.4287	0.1481	0.3663	0.6501	0.146
Jarque-Bera JB	0.4322	1.517	1.192	0.1652	0.7887	1.794	1.202	0.9306	2.03
p(normal)	0.8056	0.4684	0.551	0.9207	0.6741	0.4079	0.5482	0.628	0.3625
p(Monte Carlo)	0.7703	0.2726	0.3715	0.914	0.5725	0.2076	0.3764	0.4954	0.1615

Table S19. Homogeneity of the *C. zaitsevi* sample (n = 7) estimated by set of parametric (W, JB) and non-parametric (A) normality tests, calculated in the PAST (ver. 4.03). See Table S2. A positive test result (= sample is homogenic) is light green.

	MBH	MRH	LML	CIL	PL	UML	ZYG	P4s/d	EGW
N	7	7	7	7	7	7	7	7	7
Shapiro-Wilk W	0.9648	0.8956	0.8956	0.9865	0.944	0.8167	0.8562	0.9317	0.9234
p(normal)	0.8584	0.3054	0.3052	0.9849	0.6748	0.05968	0.1399	0.5655	0.4966
Anderson-Darling A	0.1596	0.3897	0.3744	0.1262	0.2639	0.6499	0.4994	0.2979	0.3587
p(normal)	0.9093	0.2769	0.3052	0.9685	0.5705	0.05111	0.1348	0.4891	0.3369
p(Monte Carlo)	0.9538	0.2954	0.3364	0.9949	0.6302	0.049	0.146	0.5254	0.3682
Jarque-Bera JB	0.5158	0.7184	1.051	0.3756	0.457	2.295	0.7982	0.6437	0.7334
p(normal)	0.7727	0.6982	0.5913	0.8288	0.7957	0.3175	0.6709	0.7248	0.693
p(Monte Carlo)	0.61	0.3782	0.138	0.7734	0.6881	0.0319	0.2909	0.4583	0.3488

Figure S1. Linear measurement frame and diagrammatic image of the 'glass-plate tool' for use with flatbed table scanner. (**a**) *Crocidura* skull onto the glass-plate tool (GPL), in the plane (**a1**) and profile (**a2**) projections: arrows mark touch-points: tip of P4s and petrosal bones/ectotympanic rings; **Mit**, metal items that lift a plate above the scanner surface (paper-clip parts was used); **MS**, microscopic slide; (**b**) Two dimensions of the condylar process of a mandible: **HCD**, condyle height; **LLF**, lower facet length (obtained from 3D-models use Avizo Software; just for 'pergrisea' clade spp.); (**c**) Three dimensions of the hemimandible: **LML**, lower molar row length; **MBH**, dentary height on the m2 level; **MRH**, mandibular ramus height (obtained from 2D images via GPL and tpsDIG Software); (**d**) Six dimensions of the skull: **CIL**, condylo-incisive length (between premaxilla and occipital condyle); **EGW**, external entoglenoid width; **P4s/d**, interval between inner margins of the right and left P4; **PL**, hard palate length; **UML**, upper molar row length; **ZYG**, external width of zygomatic processes of maxilla. All dimensions are in mm.

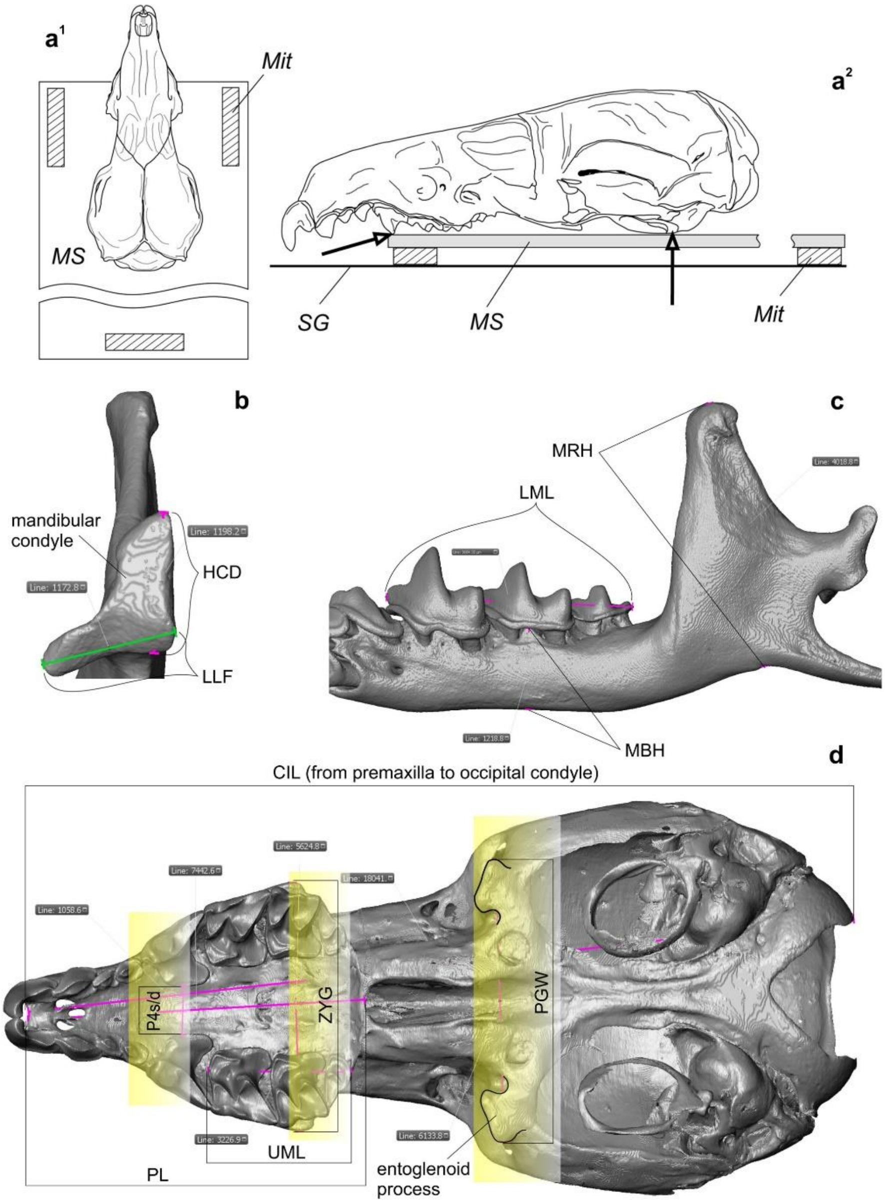

Figure S2. An example of the use of the glass-plate tool (GPL). (**a**) Skull in ventral view and right hemimandible in medial view onto GPL; (**b**) Magnified part of the hemimandible shows a satisfactory quality of the image for precise measure of the elements, e.g., LML measure, or length of each molar. An original image has resolution of 2400 dpi; used flatbed table scanner Epson Perfection v300. Abbreviations see in Figure S1.

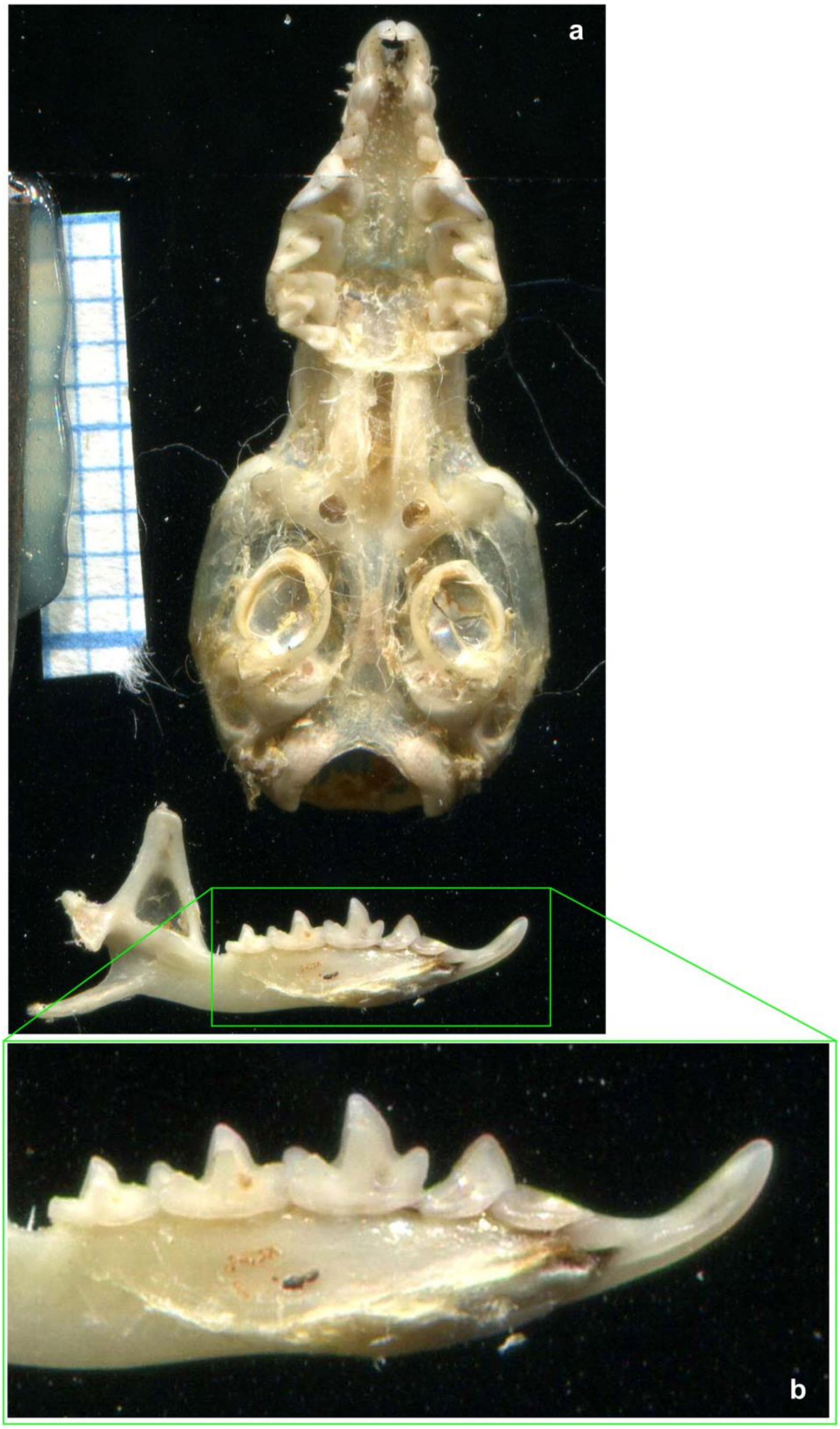

Figure S3. Steps for combining two mandibular parts through the functions of the Avizo software. The procedure was used for aligment and combining the parts of the skulls and mandible in type material of *C. armenica* (Figures 1 and 2, main text), and combining left-right hemimandibles in paratype of *C. armenica* (Figure 2, main text). An important action of the procedure is gradual aligment of two parts with 'Global Axes' and 'Transform Editor'. The action clearly shown by Dr. Jeremy Shaw on the YouTube Chanel "Microscopy Australia," in video-tutorial "Re-orientation of 3D volumes in Avizo" (https://www.youtube.com/watch?v=-S8nqc5pjNk&t=415s).

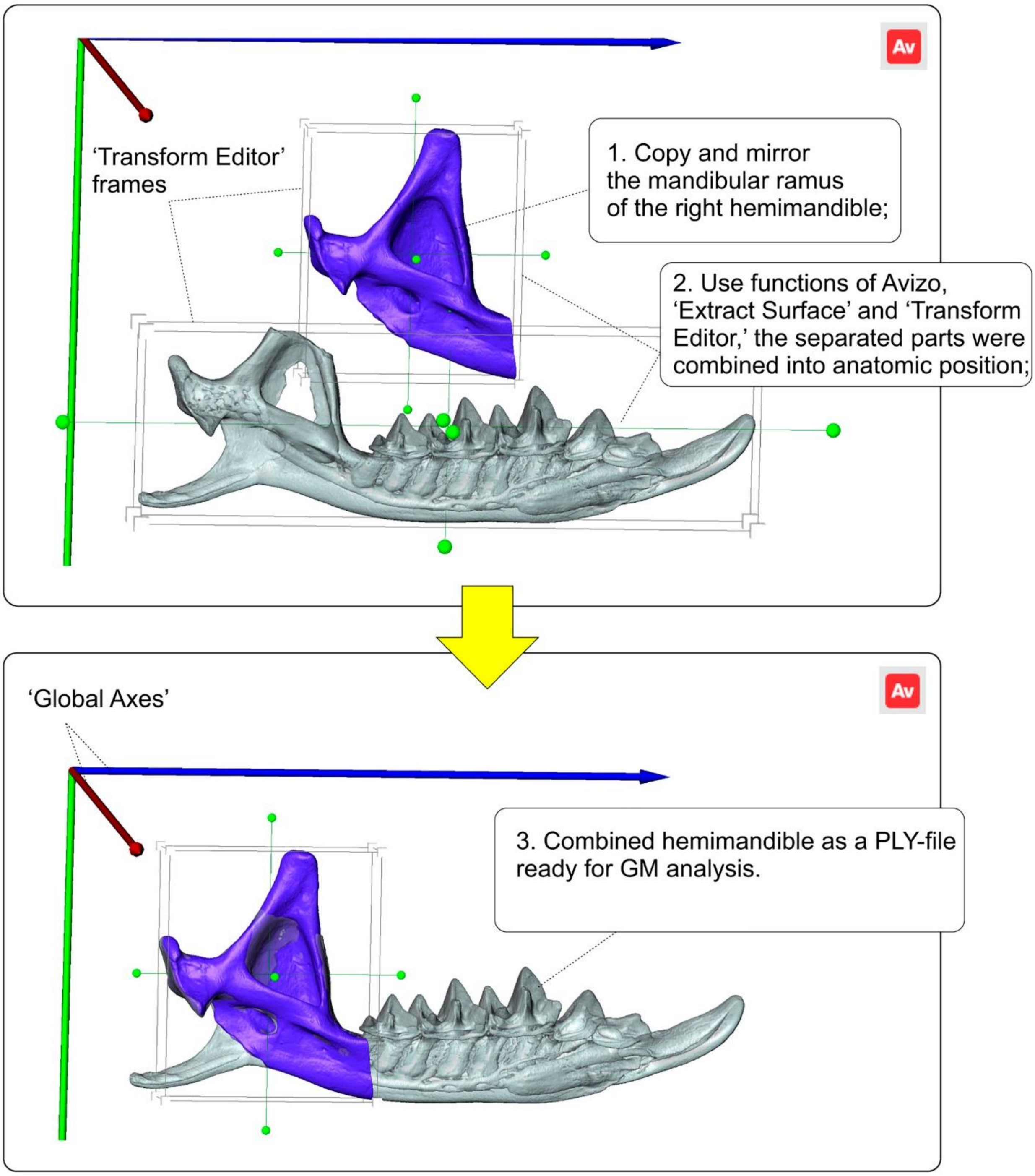

Table S20. Definition of landmarks used in the shape analysis of hemimandible (Figure 4: 'Dataset 1', main text).

No.	Landmarks (right hemimandible)
1	Contact of the most anterior edge of the a1 base with i1.
2	Contact of the most anterior edge of the p4 base with a1.
3	Most posterior point of the lingual part of the a1 base (on curvature), near to contact with p4 base.
4	Most posterior point of the lingual part of the p4 base (on curvature), near to contact with m1 base.
5	Most posterolingual point of the m1 base.
6	Most posterolingual point of the m2 base.
7	Most anterior point of the mandibular foramen edge (on curvature).
8	Most medial point of the lower facet of the mandibular condyle (at central part of the facet outer curvature; see Figure 4a[2]).
9	Most upperward prominent point of the upper facet of the mandibular condyle.
10	Most medial point of the lower condylar facet (opposite 7th lm).
11	Most posterior point of the mental foramen edge (on curvature).
slm 12–31	Semilandmarks line starts at the posterior edge of the m3 posterior alveolus to the uppermost point of the coronoid process (see Figure 4a[1]).
slm 32–61	Semilandmarks line starts at anterior tip of the mandibular symphysis to the middle part of the notch of the lower dentary-angular process profile.
**Total: 61 lms.**	

Table S21. Definition of landmarks used in the shape analysis of skull (Figure 4: 'Dataset 2', main text).

No.	Landmarks (right hemimandible)
1	Internal surface of the sphenoid, along a sagittal line on the level of internal entrance of the optic canal (Figure S4).
2	Internal surface of the ethmoid, most anterodorsal point of the cribriform plate along a sagittal line (Figure S4).
3	Most anterior point of the nasal bones, along a sagittal line (within nasal aperture).
4	Most anterior point of the premaxilla, along a sagittal line.
5	Middle position on the ventral surface of the postpalatine torus, along a sagittal line.

6	Most anterior (protruding) point of the A1 alveolus edge (anterobuccal root; Figure S4).
7	Most anterior point of the P4 alveolus edge (Figure S4).
8	Tip of the zygomatic process of the maxilla.
9	Most posterior point of the foramen ovale edge (on curvature).
10	Most anterior point of the entoglenoid process.
11	Most lateral point of the upper articular facet of the entoglenoid fossa.
12	Middle position on the anterior edge of the lateral wall of the infraorbital canal (on curvature).
13	Middle position on the posterior edge of the lateral wall of the infraorbital canal (on curvature).
14	Most posterior point of the M3 alveolus edge (posterior root).
**Total: 14 lms.**	

Figure S4. Explanatory drawings of the landmarks position on the premaxilla and maxilla bones surface (a) and inner surface of the cranium (b). (**a^1**) Rostral part of the skull in occlusal view, with the transparent teeth: 6th landmark is located on the anterior edge of A1 alveolus; 7th landmark is located on the anterior edge of P4 alveolus; (**a^2**) Landmarks position, with the transparent teeth; (**a^3**) Landmarks position without the teeth; (**b**) Rostral part of the skull in the posterolateral view for show inner surfaces of ethmoid and sphenoid with key structures and 1st and 2nd landmarks position. Unscaled. Images obtained from the 3D models of *C. suaveolens* (a, ZIN 73665) and *C. zarudnyi* (b, ZIN 6506).

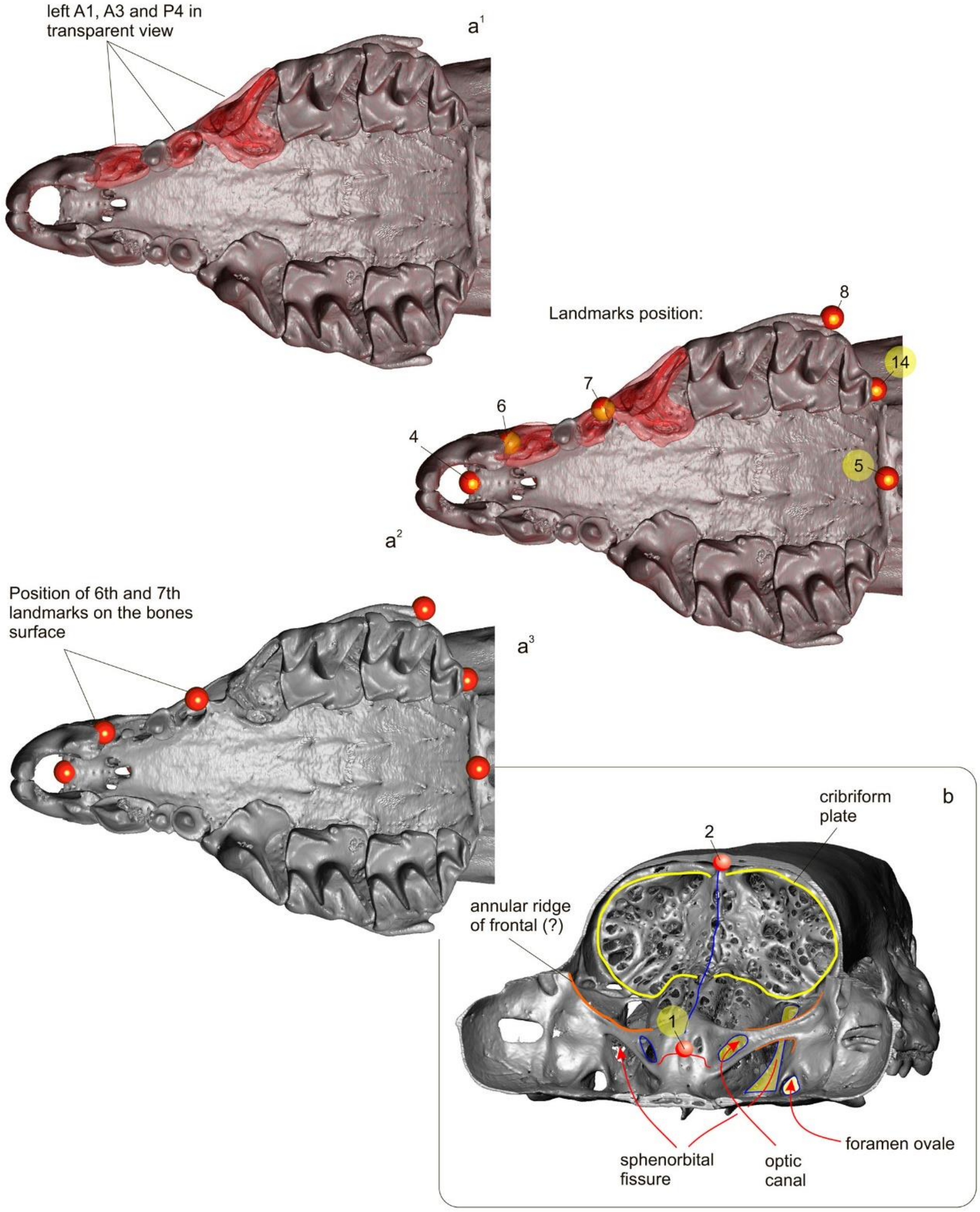

Figure S5. *Crocidura armenica* ZIN 45277, skull fragment.

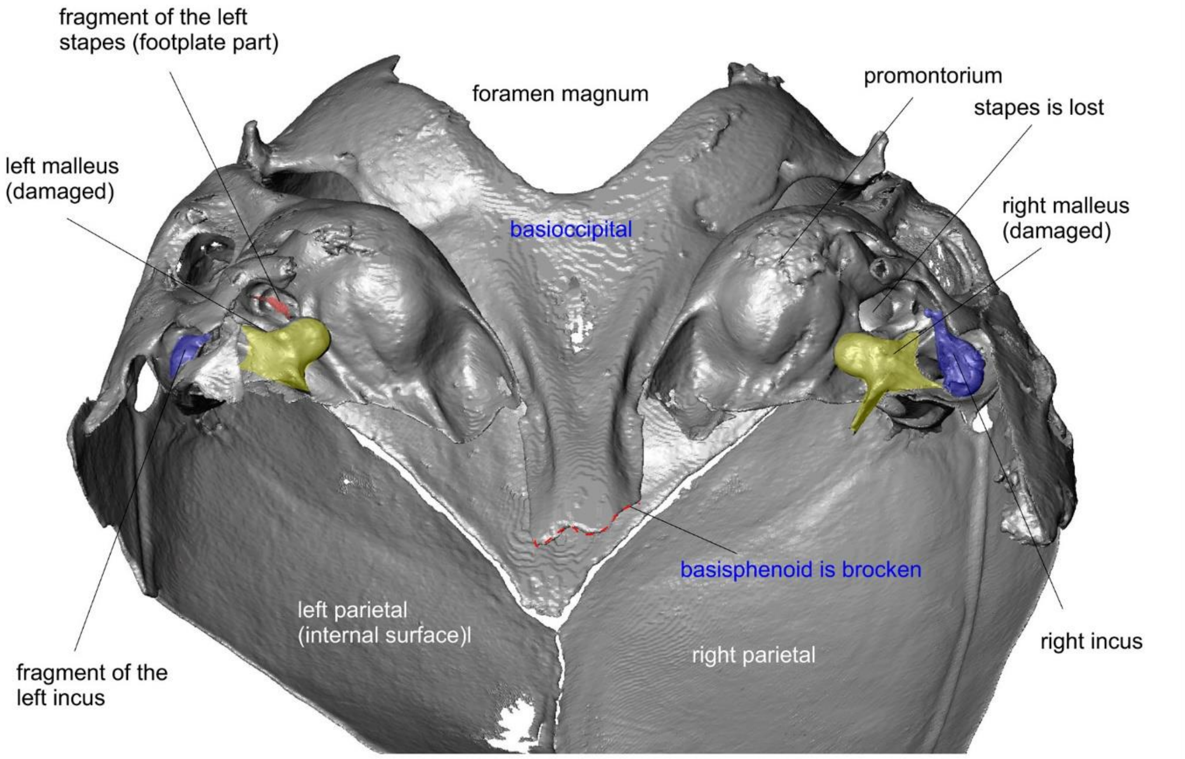

Figure S5. Fragment of the braincase of holotype of *Crocidura armenica* ZIN 45277 in ventral view. Auditory ossicles are shown by transparent colors. Unscaled.

Figure S6. *Crocidura armenica* ZIN 45277, skull.

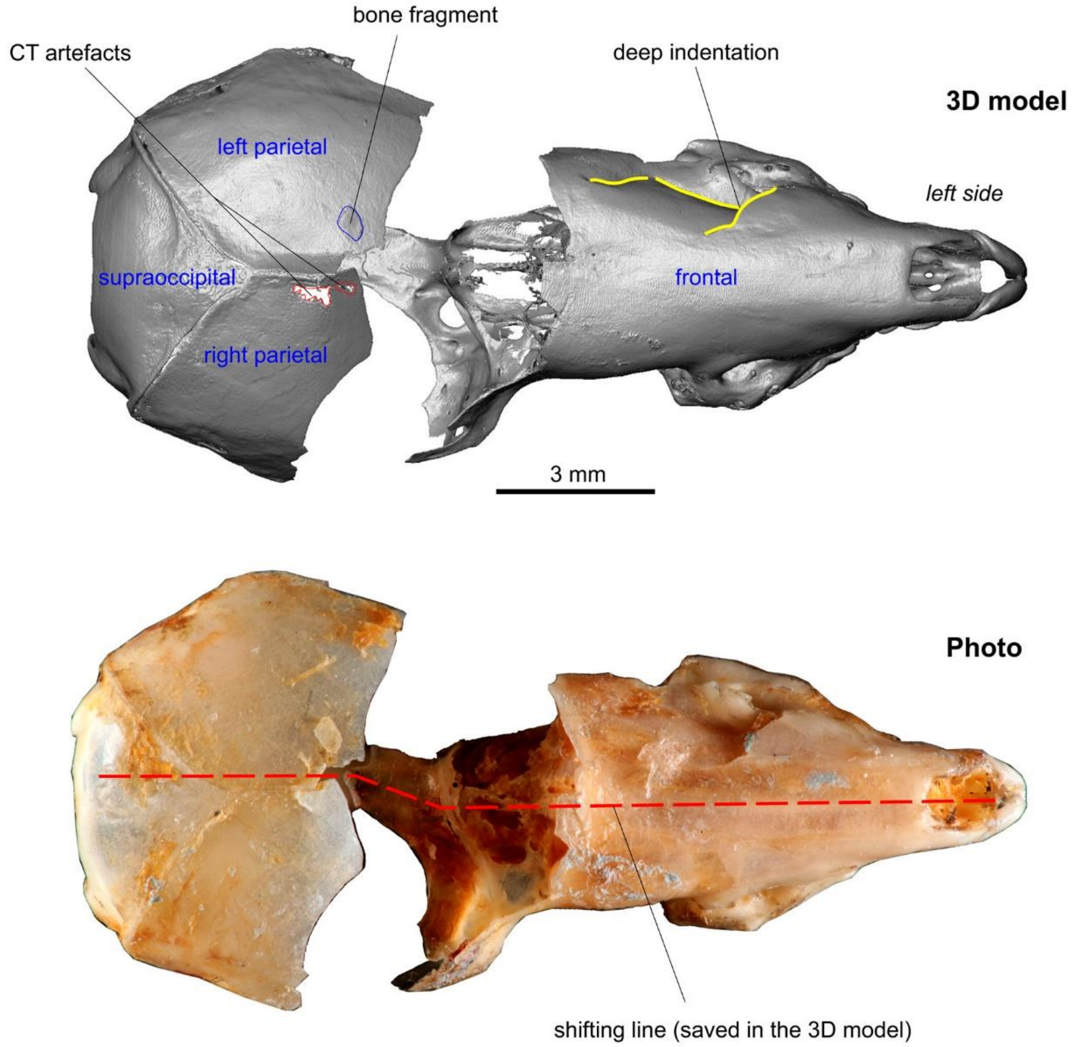

Figure S6. Skull of holotype of *Crocidura armenica* ZIN 45277 in dorsal view: Image of the 3D model (above) and original photo of the type (below). Scale bar is 3 mm.

Figure S7. *Crocidura armenica* ZIN 45277, skull fragment.

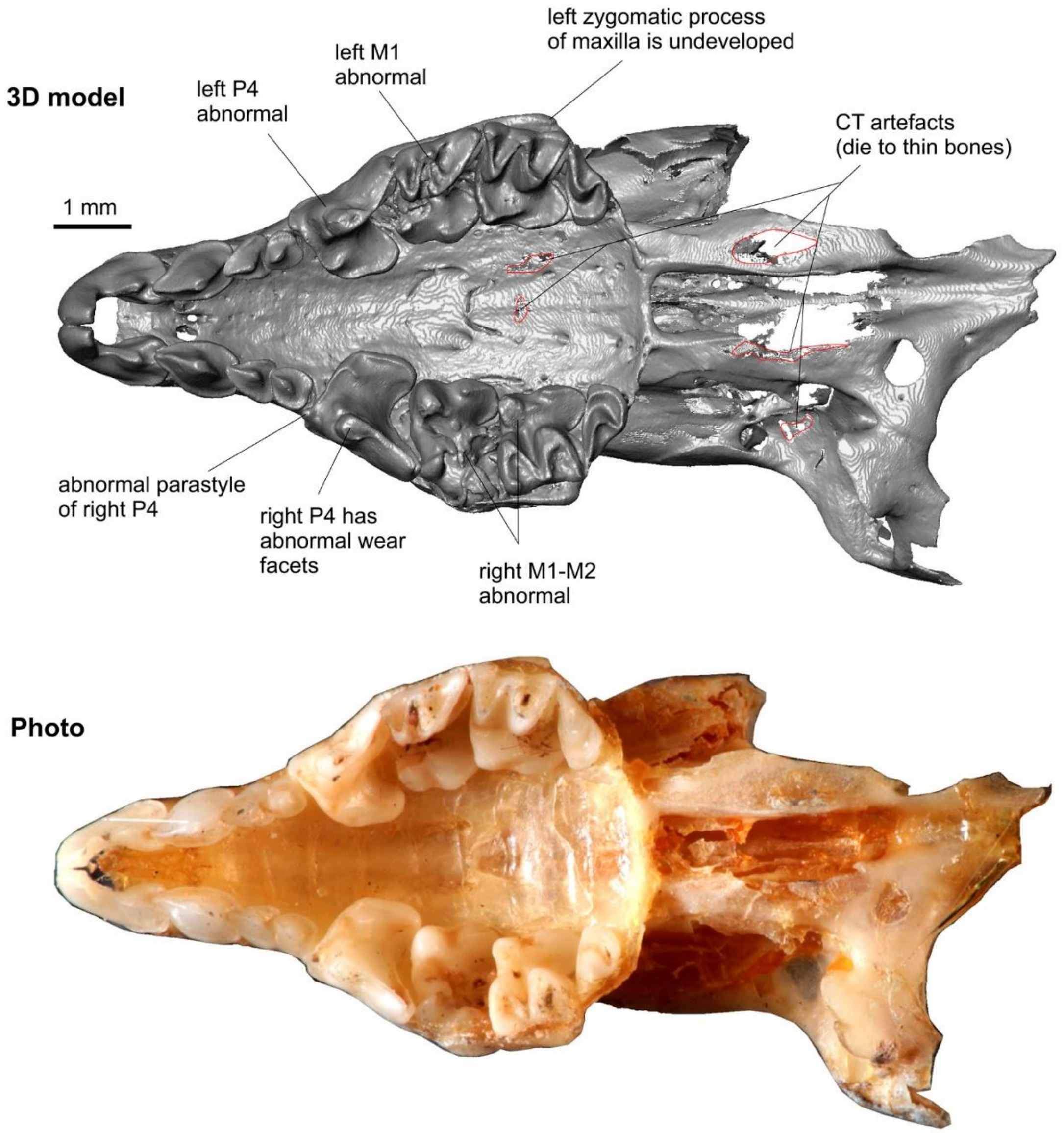

Figure S7. Anterior fragment of the holotype skull of *Crocidura armenica* ZIN 45277 in ventral view: Image of the 3D model (above) and original photo of the type (below). Scale bar is 1 mm.

Figure S8. *Crocidura armenica* ZIN 45277, upper teeth.

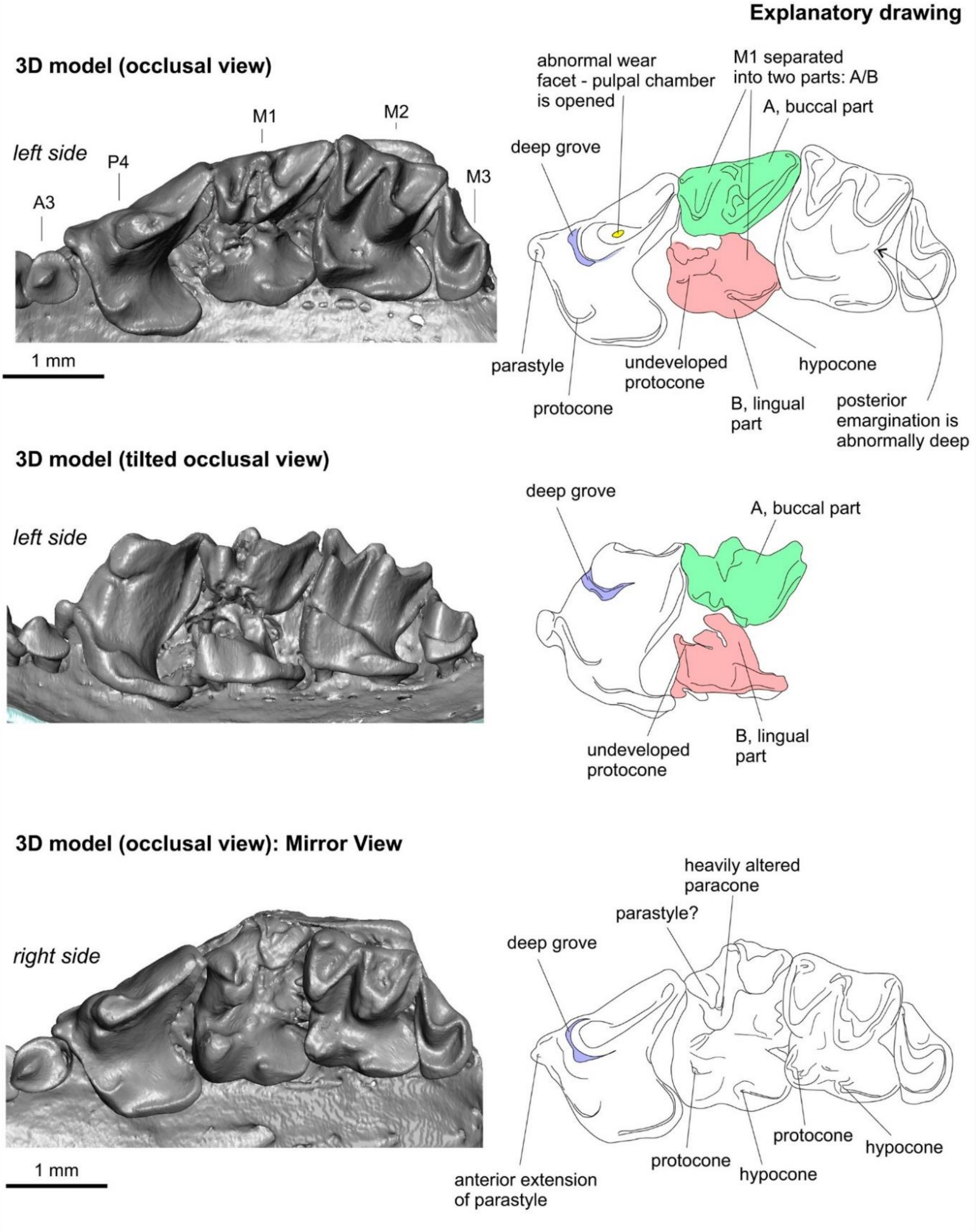

Figure S8. Upper tooth rows of the holotype of *Crocidura armenica* ZIN 45277. Provided images of the 3D model (right) of the left teeth — upper and middle lines, and the right teeth — lower line; and explanatory drawings with marking dental features and abnormalities. Scale bars are 1 mm.

Figure S9. *Crocidura armenica* ZIN 45277, left hemimandible and lower teeth.

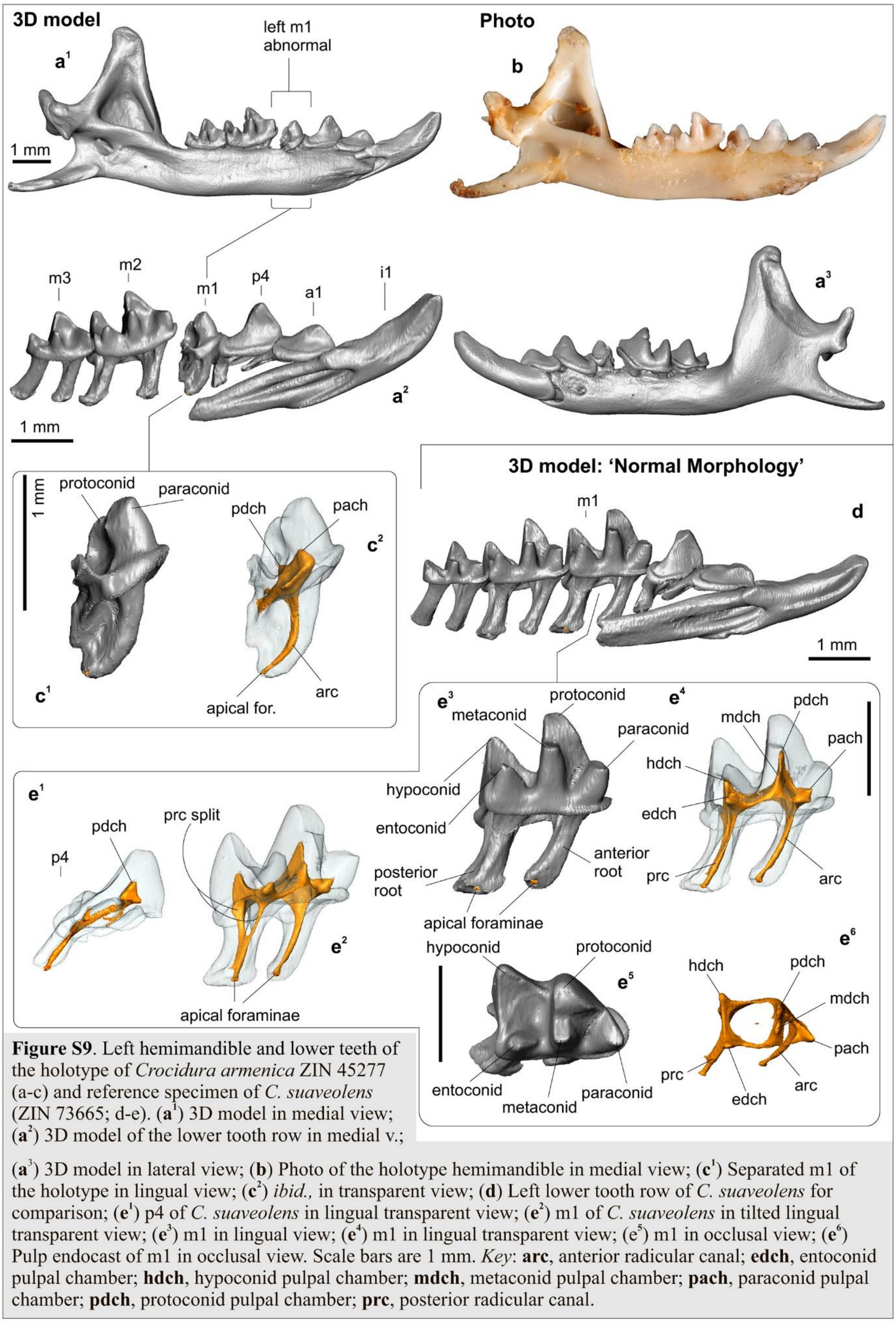

Figure S9. Left hemimandible and lower teeth of the holotype of *Crocidura armenica* ZIN 45277 (a-c) and reference specimen of *C. suaveolens* (ZIN 73665; d-e). (**a**[1]) 3D model in medial view; (**a**[2]) 3D model of the lower tooth row in medial v.; (**a**[3]) 3D model in lateral view; (**b**) Photo of the holotype hemimandible in medial view; (**c**[1]) Separated m1 of the holotype in lingual view; (**c**[2]) *ibid.,* in transparent view; (**d**) Left lower tooth row of *C. suaveolens* for comparison; (**e**[1]) p4 of *C. suaveolens* in lingual transparent view; (**e**[2]) m1 of *C. suaveolens* in tilted lingual transparent view; (**e**[3]) m1 in lingual view; (**e**[4]) m1 in lingual transparent view; (e[5]) m1 in occlusal view; (**e**[6]) Pulp endocast of m1 in occlusal view. Scale bars are 1 mm. *Key*: **arc**, anterior radicular canal; **edch**, entoconid pulpal chamber; **hdch**, hypoconid pulpal chamber; **mdch**, metaconid pulpal chamber; **pach**, paraconid pulpal chamber; **pdch**, protoconid pulpal chamber; **prc**, posterior radicular canal.

Figure S10. *Crocidura armenica* ZIN 45277, right second lower molar (unscaled).

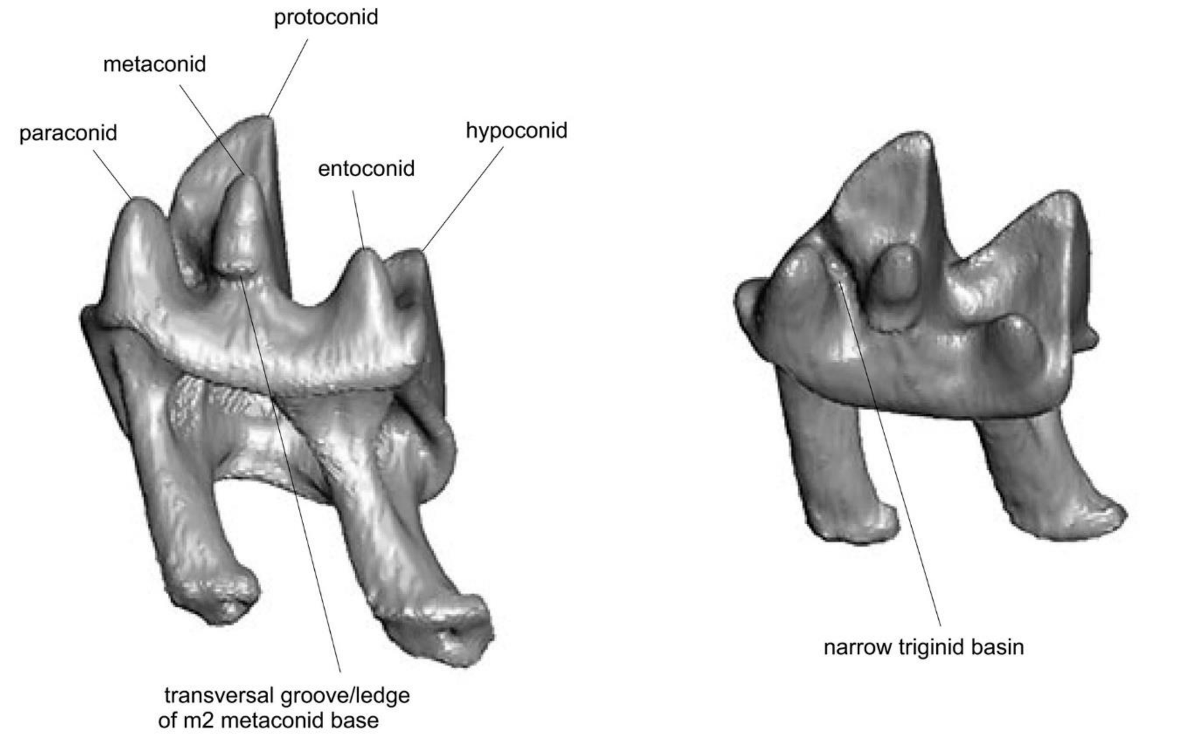

Figure S10. 3D model of the right isolated second lower molar of the *Crocidura armenica* ZIN 45277 holotype in lingual (left) and tilted lingual (right) views. Unscaled.

Figure S11. *Crocidura armenica* ZIN 45277, right hemimandible and lower teeth.

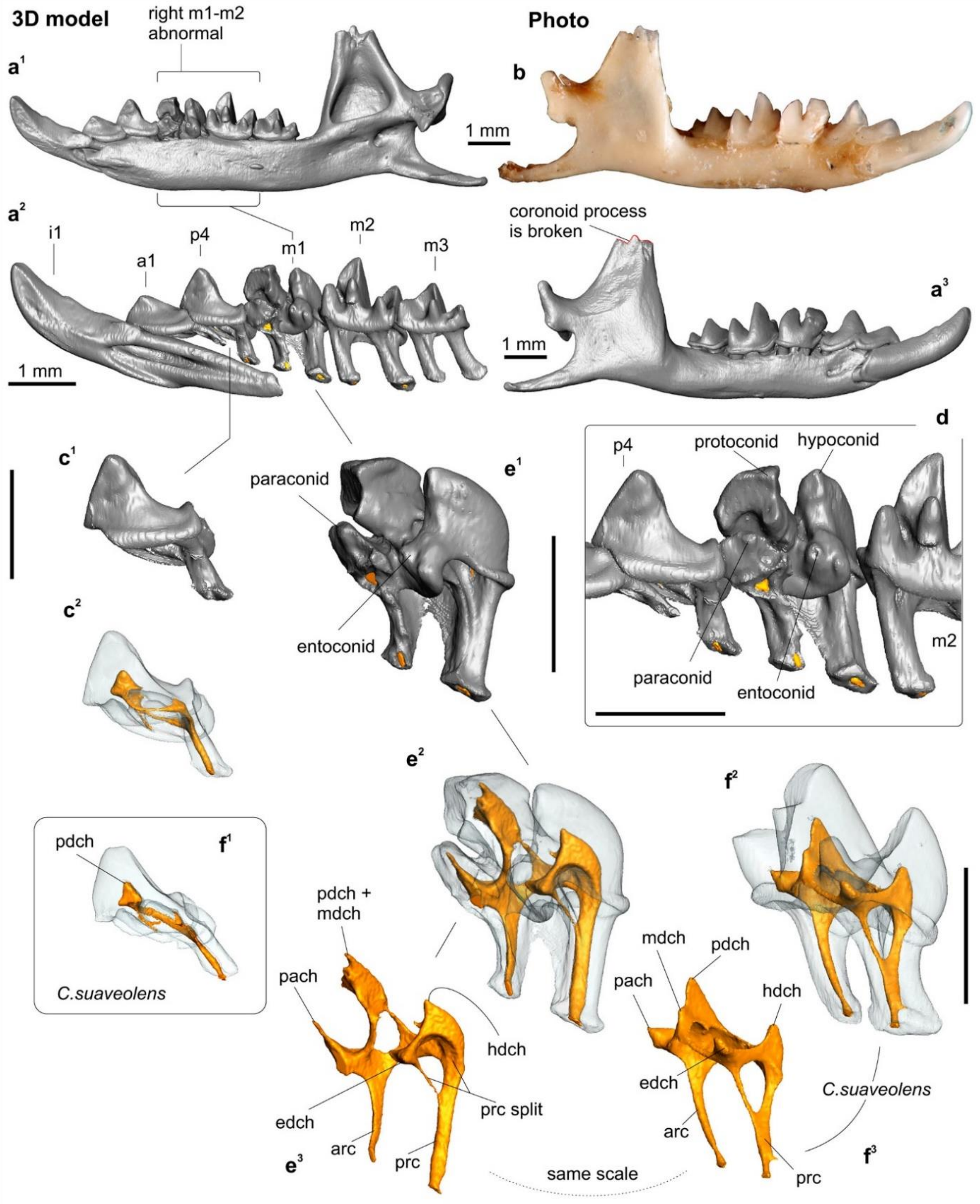

Figure S11. Right hemimandible and lower teeth of the holotype of *Crocidura armenica* ZIN 45277 (a-e) and teeth of reference specimen of *C. suaveolens* (ZIN 73665; f). (**a**[1]) 3D model in medial view; (**a**[2]) 3D model of the lower tooth row in medial view; (**a**[3]) 3D model in lateral view; (**b**) Photo of the holotype hemimandible in lateral view; (**c**[1]) Separated p4 in lingual view; (**c**[2]) *ibid.*, in transparent view; (**d**) Fragment of the tooth row of the holotype in lingual view (see m1 tilting in relation to p4 and m2); (**e**[1]) m1 in tilted lingual view; (**e**[2]) *ibid.*, in transparent view; (**e**[3]) Pulp endocast of m1 in same view to 'e[2]'; (**f**[1]) p4 of *C. suaveolens* in lingual transparent view; (**f**[2]) m1 of *C. suaveolens* in tilted lingual transparent view; (**f**[3]) Pulp endocast of m1 of *C. suaveolens*. Scale bars are 1 mm. *Key* see in Figure S9.

Figure S12. *Crocidura armenica* ZIN 55321, skull fragment.

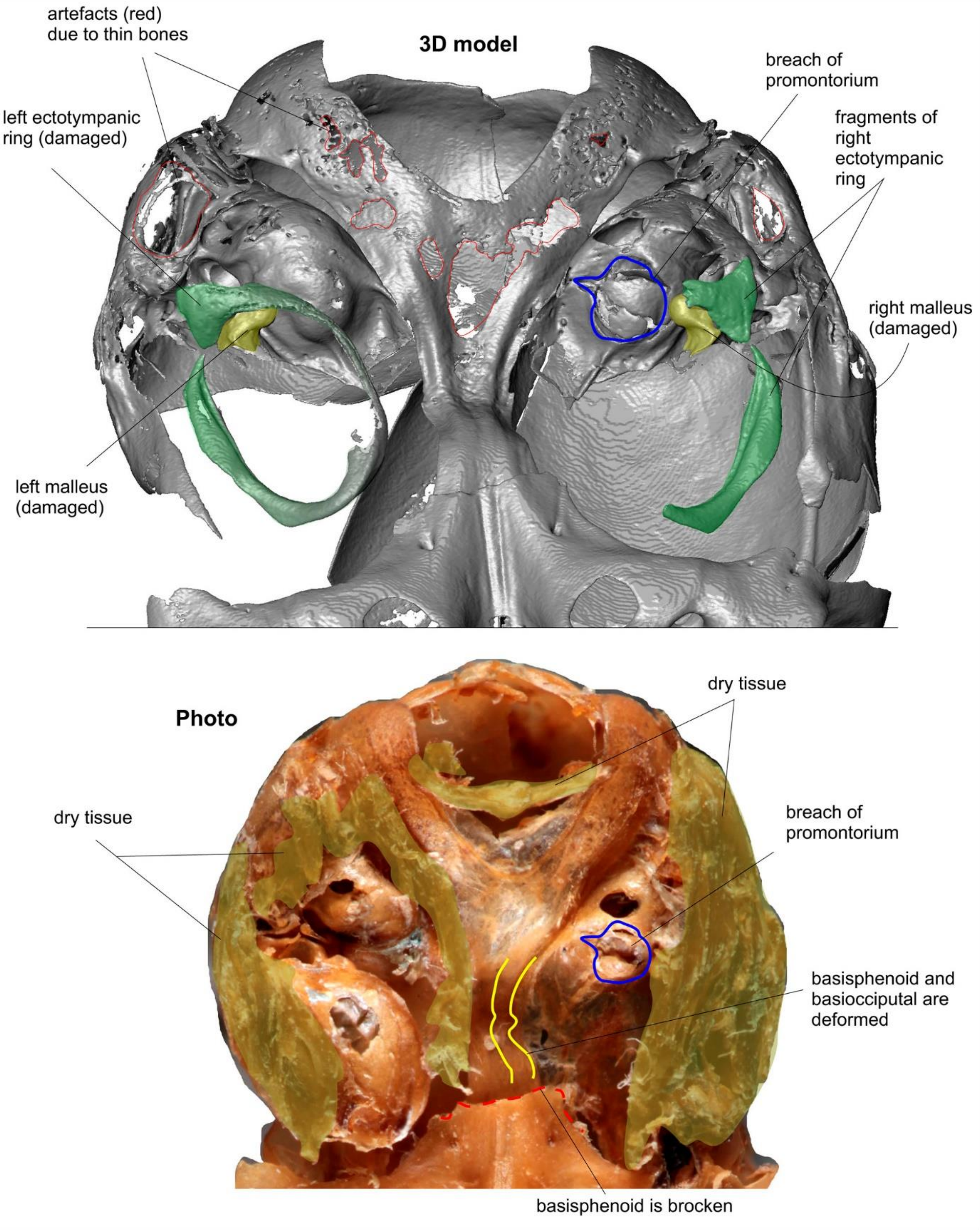

Figure S12. Fragment of the braincase of paratype of *Crocidura armenica* ZIN 55321 in ventral view: Image of the 3D model (above) and original photo of the type (below). Auditory ossicles, ectotympanic rings and tissues are shown by transparent colors. Unscaled.

Figure S13. *Crocidura armenica* ZIN 55321, skull.

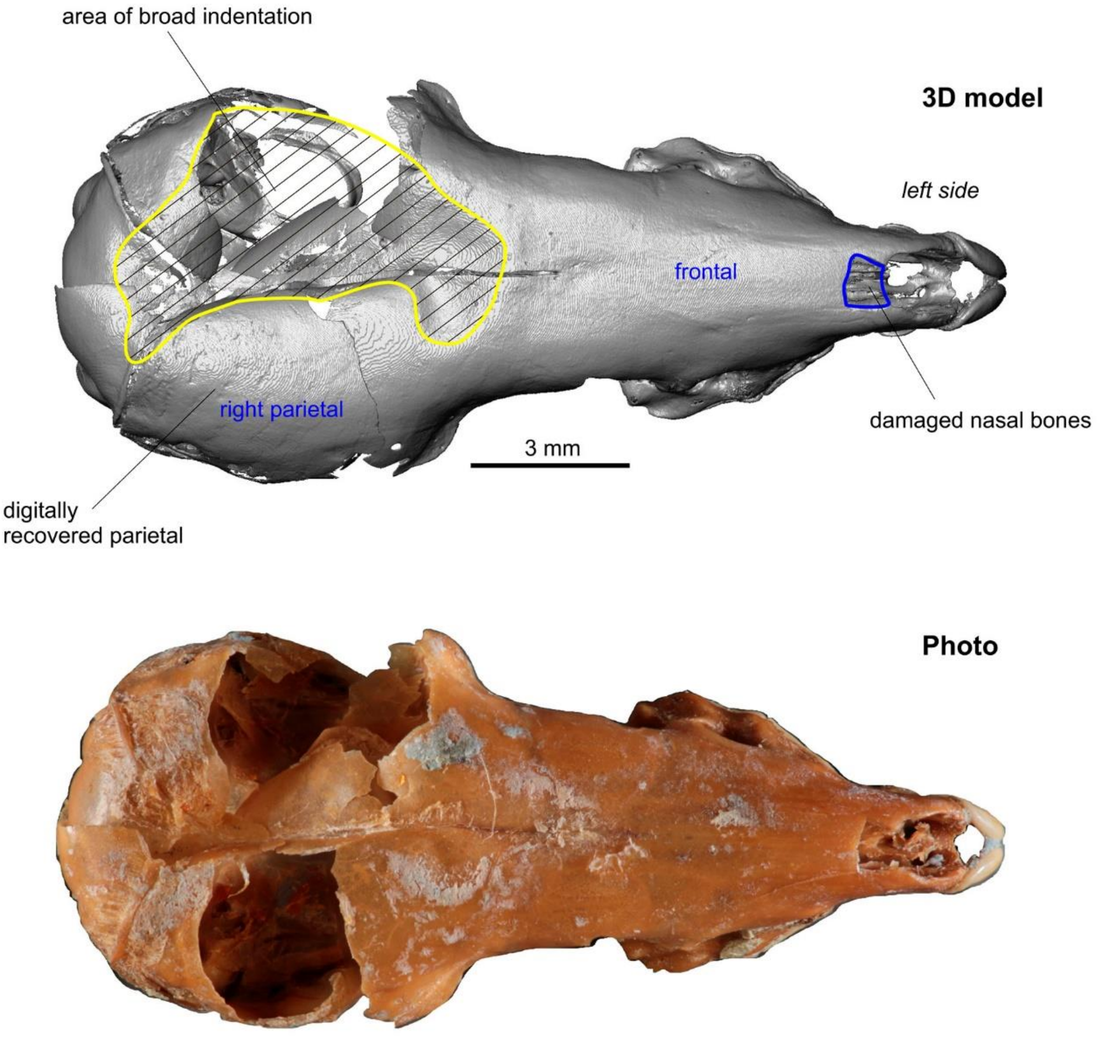

Figure S13. Skull of paratype of *Crocidura armenica* ZIN 55321 in dorsal view: Image of the 3D model (above) and original photo of the type (below). Scale bar is 3 mm.

Figure S14. *Crocidura armenica* ZIN 55321, skull.

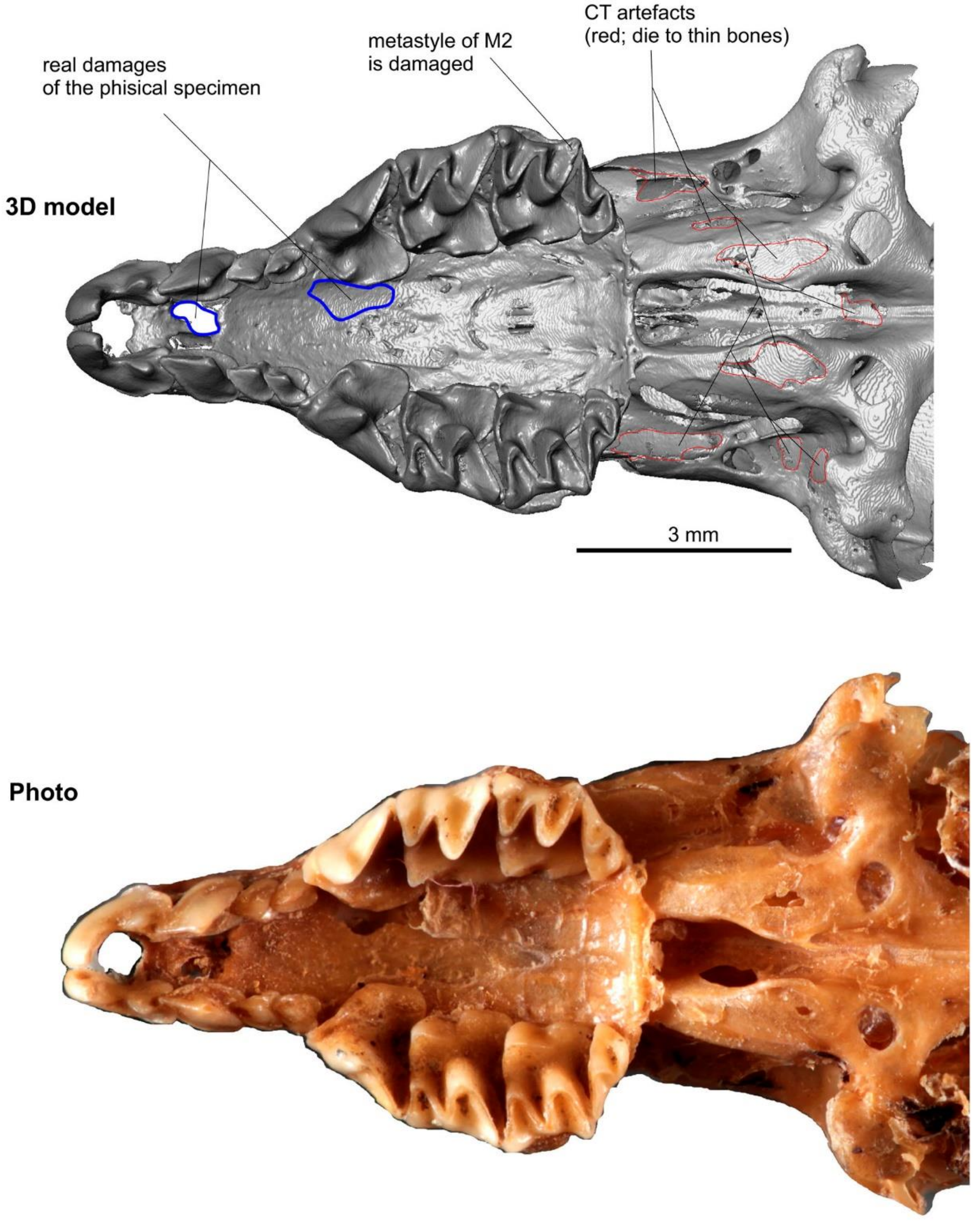

Figure S14. Anterior fragment of the paratype skull of *Crocidura armenica* ZIN 55321 in ventral view: Image of the 3D model (above) and original photo of the type (below). Scale bar is 3 mm.

Figure S15. *Crocidura armenica* ZIN 55321, skull.

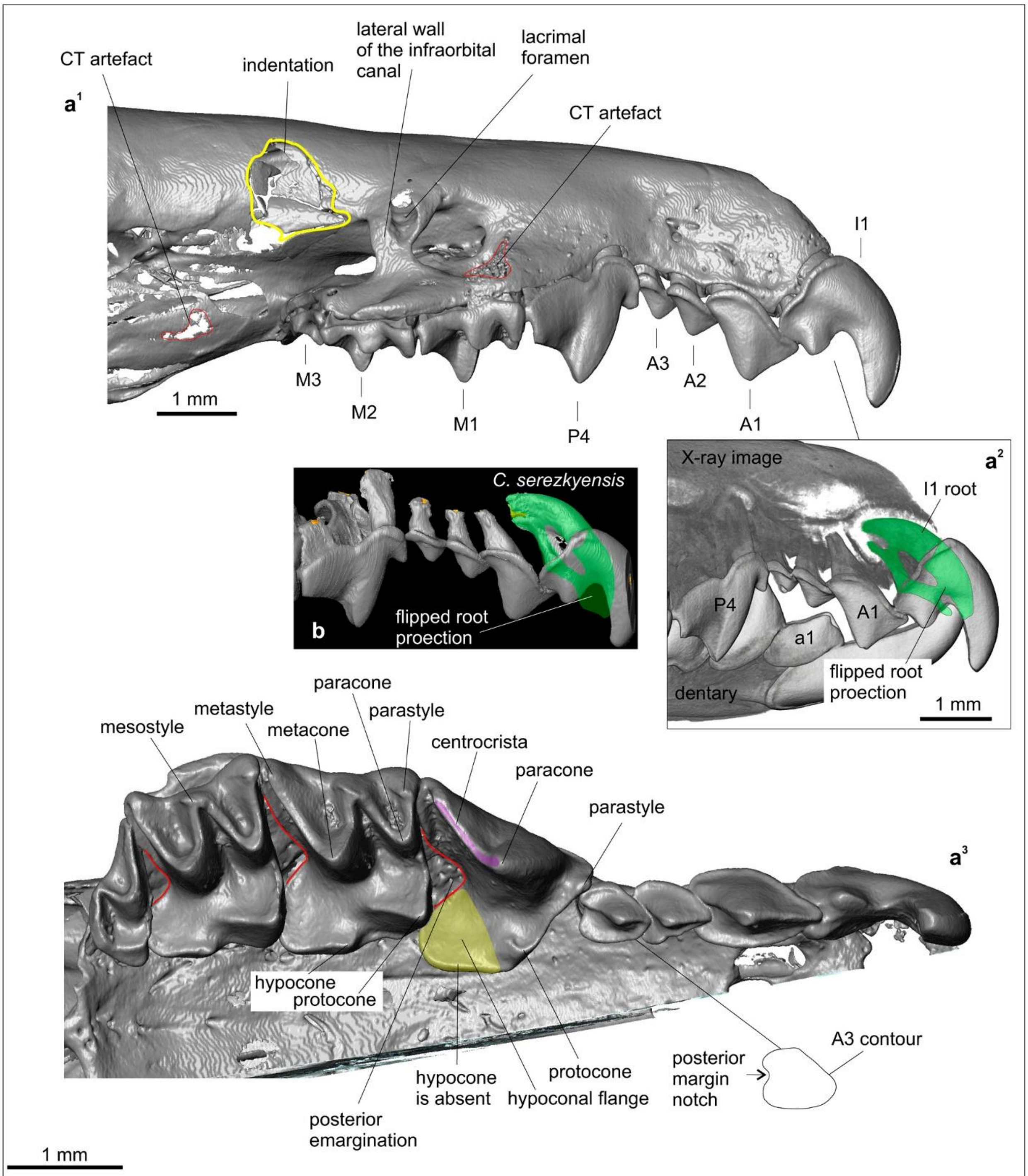

Figure S15. Anterior fragment of the paratype skull of *Crocidura armenica* ZIN 55321 in lateral view, its right upper dentition (a) and fragment of upper dentition of *C. serezkyensis* ZIN 77431 (b). (**a**^{1}) Muzzle in lateral view (right side); (**a**^{2}) X-ray image of the muzzle in lateral view, with colour marking of the incisor root; flipped root projection shows root size in relation crown size; (**a**^{3}) Muzzle right fragment in occlusal view; (**b**) *C. serezkyensis* isolated right upper teeth, I1-P4, in lateral view. All images are screenshots of 3D-models. Scale bars are 1 mm.

Figure S16. *Crocidura armenica* ZIN 55621, left hemimandible and lower teeth.

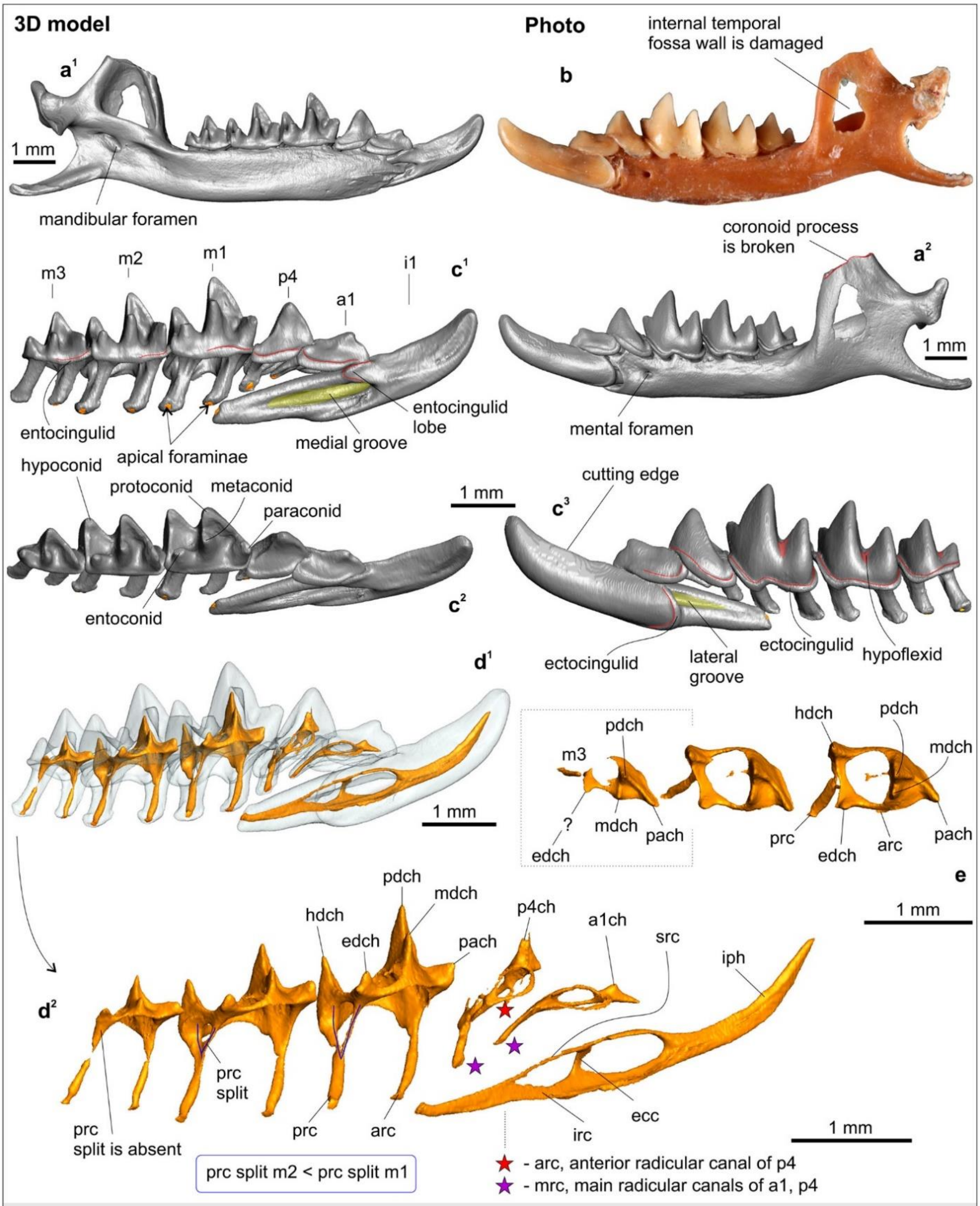

Figure S16. Left hemimandible and lower teeth of the paratype of *Crocidura armenica* ZIN 55321. (**a^1**) 3D model in medial view; (**a^2**) 3D model in lateral view; (**b**) Photo of the paratype hemimandible in lateral view; (**c^1**) 3D model of the lower tooth row in medial view; (**c^2**) 3D model of the lower tooth row in occlusal view; (**c^3**) 3D model of the lower tooth row in lateral view; (**d^1**) 3D model of the lower tooth row in posteromedial transparent view; (**d^1**) Pulp endocast of the lower tooth row in posteromedial view; (**e**) Pulp endocast of the lower molars in occlusal view. Scale bars are 1 mm. *Key*: **a1ch**, chamber of the first lower antemolar; **arc**, anterior radicular canal of p4; **ecc**, ectocingulid commissura; **iph**, incisor pulpal horn; **irc**, incisor inferior radicular canal; **mrc**, main radicular canal of a1 and p4; **src**, incisor superior radicular canal; also see in Figure S9.

Figure S17. *Crocidura armenica* ZIN 45277 (above) and *C. arispa* NMW 13284 (below; photo by Alexander Bibl), dry stuffed skins. Original label of *C. armenica* inserts into a center of the image.

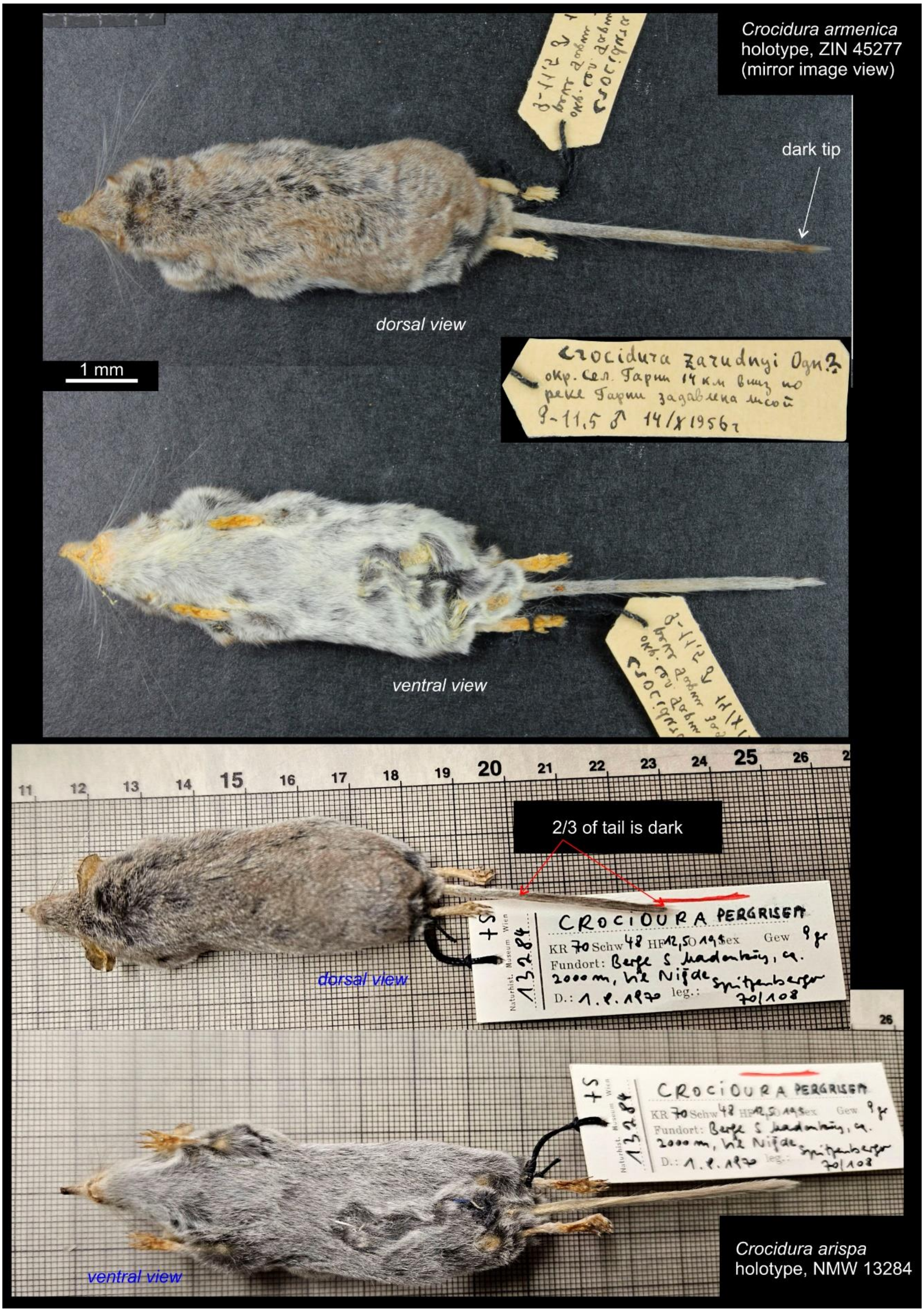

Figure S18. *Crocidura serezkyensis* ZIN 77431 dry skin. Unscaled.

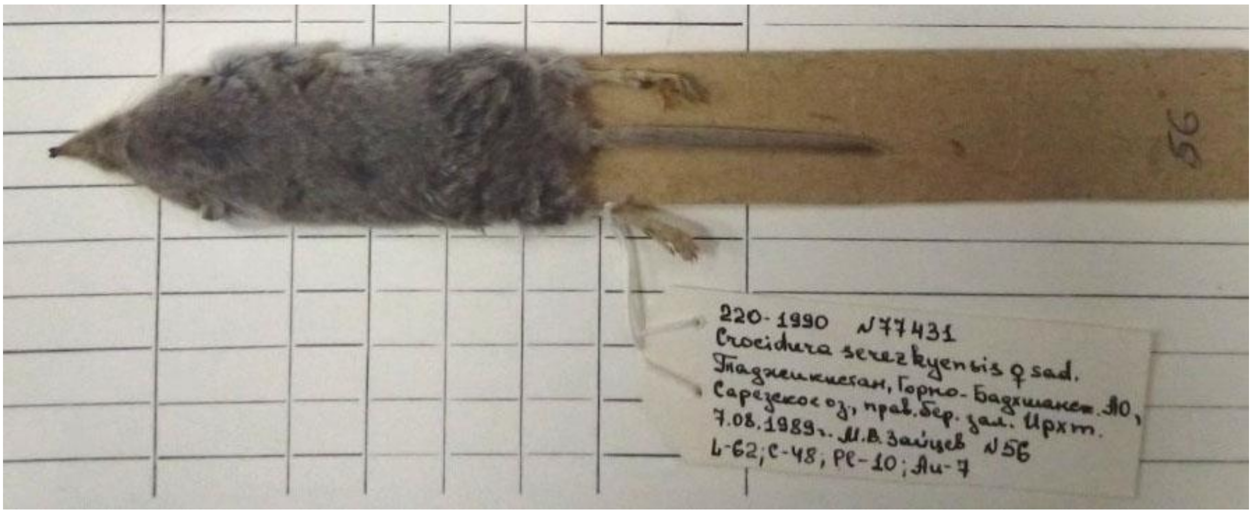

Figure S19. Bivariate (a) and univariate (b, c) plots of linear characteristic comparisons of 14 *Crocidura* species, including species of 'pergrisea' group and two outgroup species, *Suncus* and *Sorex*. (**a**) Bivariate plot of species comparison by relation of upper molar length (UML) to mandibular ramus height (MRH); (**b**) Univariate plot of species comparisons by condylo-incisive length (CIL); (**c**) Univariate plot of species comparisons by external width of zygomatic processes of maxilla (ZYG). *Key*: **α**, 3D model image of upper molars in occlusal view with a marked UML dimension; **β**, 3D model image of a mandibular ramus in lateral view with a marked MRH dimension; **γ**, 3D model image of a skull in ventral view with a marked CIL dimension; **Δ**, 3D model image of a skull fragment in ventral view with a marked ZYG dimension; **A**, a conventionally difference between species; **W**, Shapiro-Wilk normality test values, with p-value (p) (Tabs S12, S18); also see Table S1.

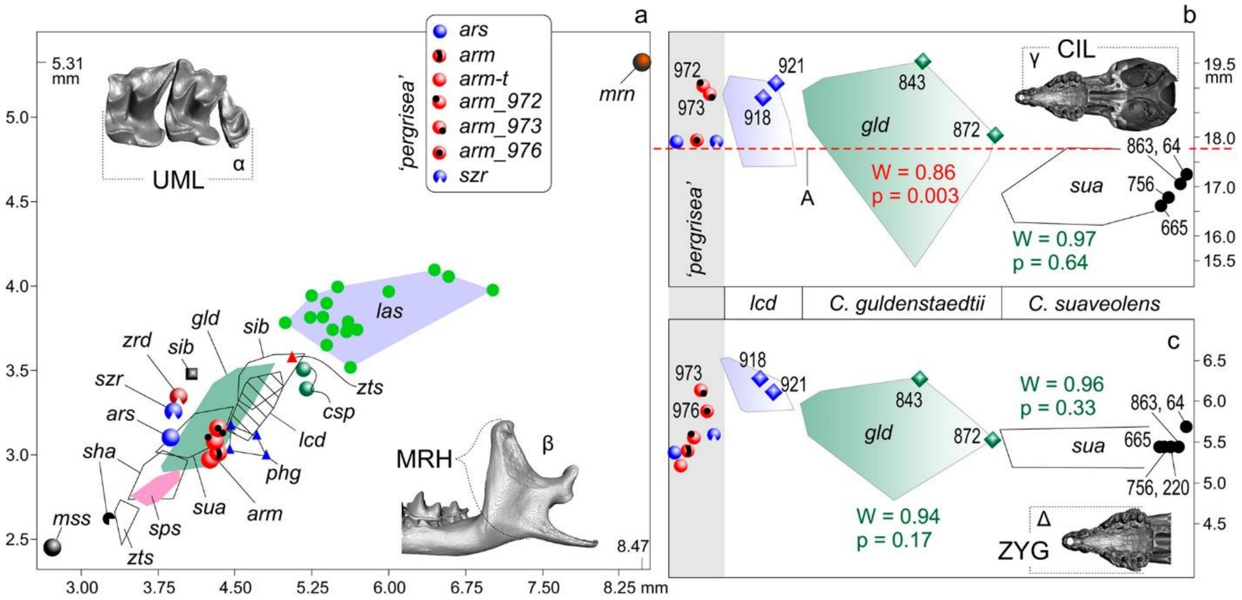

Figure 20. Comparison of skulls. All specimens in the lateral view. (**a**) *C. armenica* (paratype); (**b**) *C. armenica* from Julfa; (**c**[1]) *C. arispa* (holotype); (**c**[2]) posterior fragment of the skull; (**c**[3]) *ibid*., shown bones deformation; (**d**) *C. serezkyensis*. *Key*: **A**, downward bend of the dorsal profile. Same-scaled; scale bar is 5 mm.

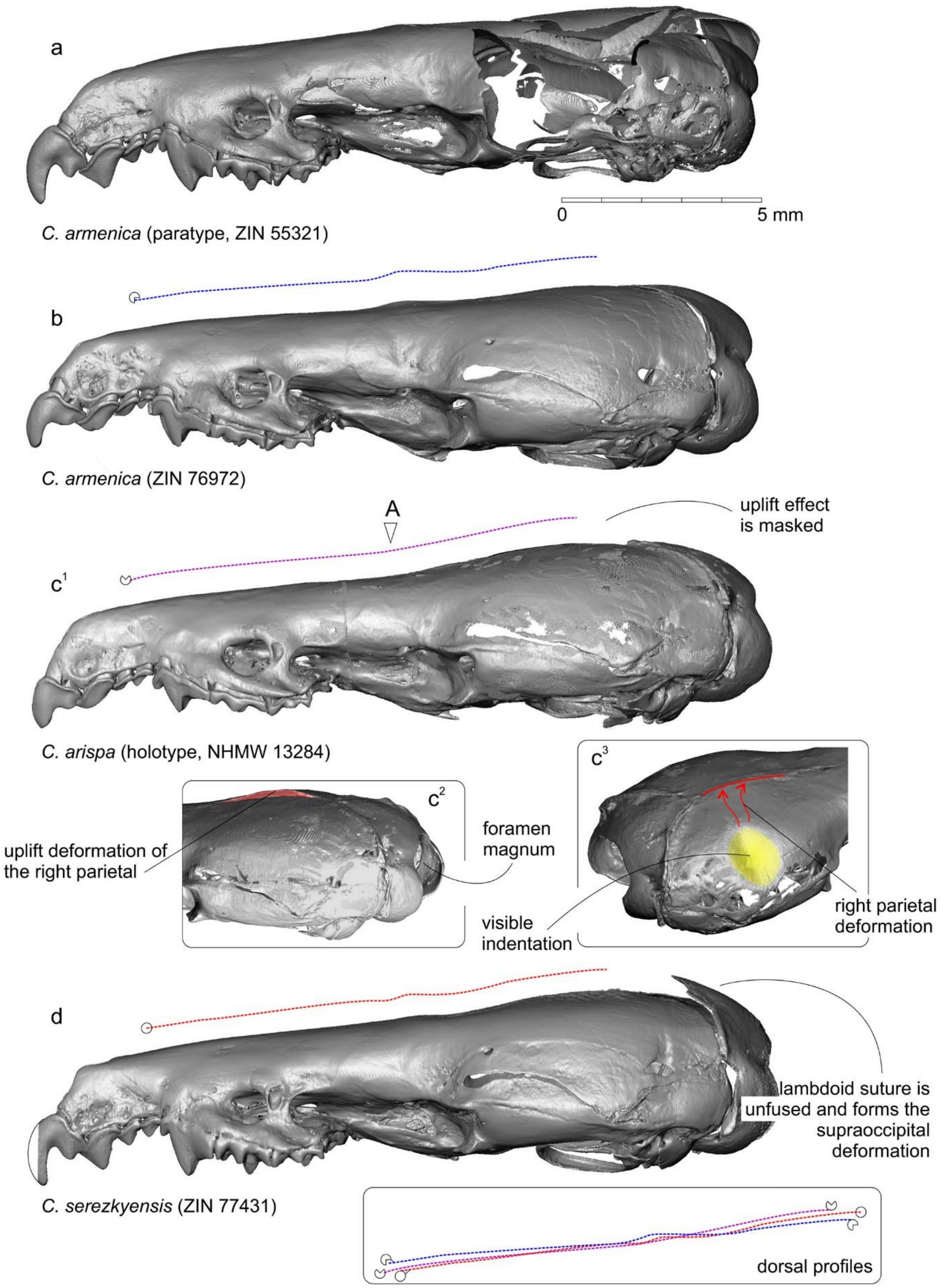

Figure S21. General information on the principal component analyses performed for the mandibular shape (a) and cranial shape (b) comparisons. The components located above the 'broken-stick' (red line) were used for the shape changes discussion (see Material and methods for explanation). Tables represent component loadings.

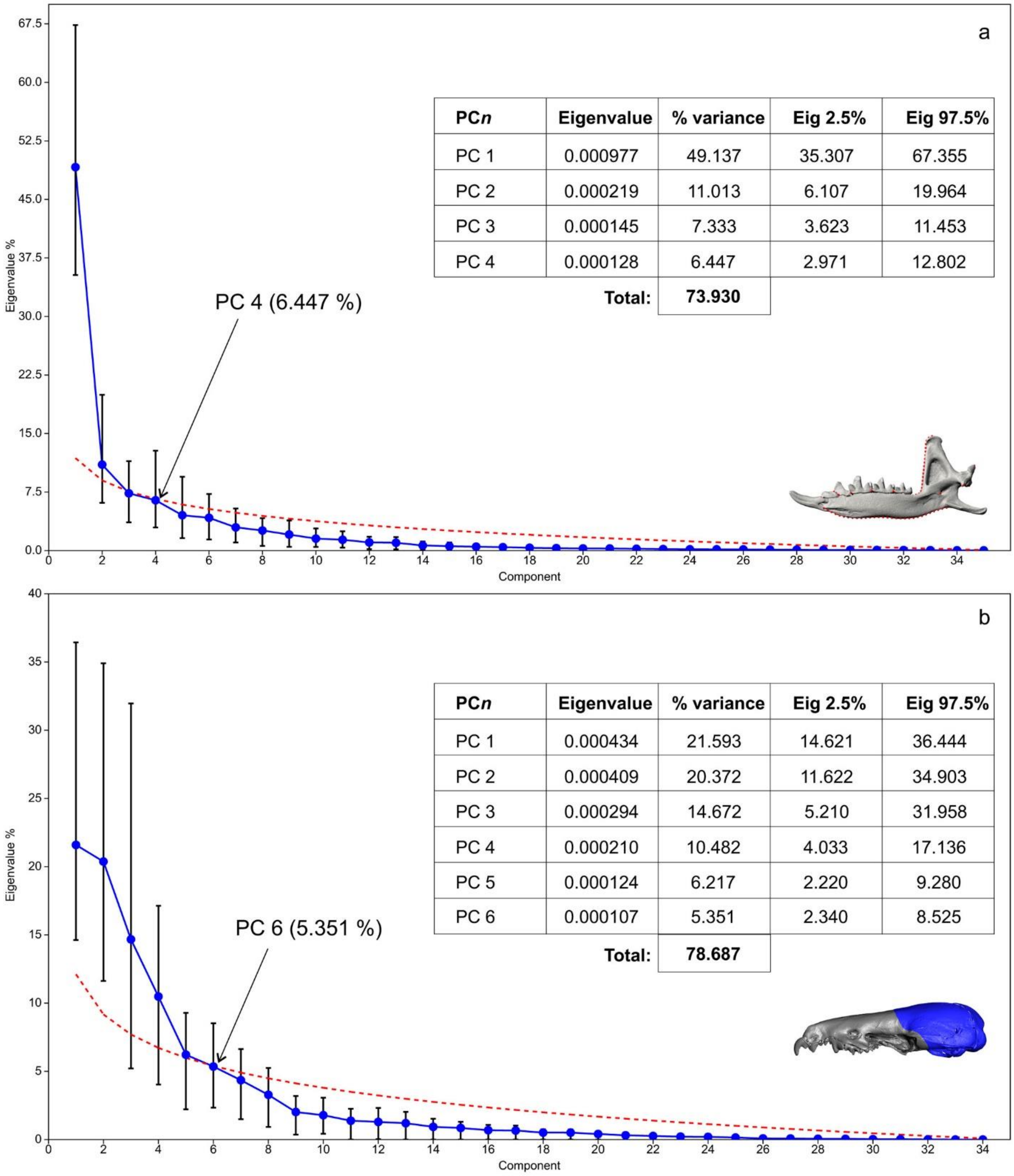

(a)

PC*n*	Eigenvalue	% variance	Eig 2.5%	Eig 97.5%
PC 1	0.000977	49.137	35.307	67.355
PC 2	0.000219	11.013	6.107	19.964
PC 3	0.000145	7.333	3.623	11.453
PC 4	0.000128	6.447	2.971	12.802
	**Total:**	**73.930**		

(b)

PC*n*	Eigenvalue	% variance	Eig 2.5%	Eig 97.5%
PC 1	0.000434	21.593	14.621	36.444
PC 2	0.000409	20.372	11.622	34.903
PC 3	0.000294	14.672	5.210	31.958
PC 4	0.000210	10.482	4.033	17.136
PC 5	0.000124	6.217	2.220	9.280
PC 6	0.000107	5.351	2.340	8.525
	**Total:**	**78.687**		

Figure S22. Mandibular shape transformation: explanation of the dorsal profile changes for first component between positive end (above; *Suncus*-like morphotype) and negative end (below; *Sorex*-like morphotype).

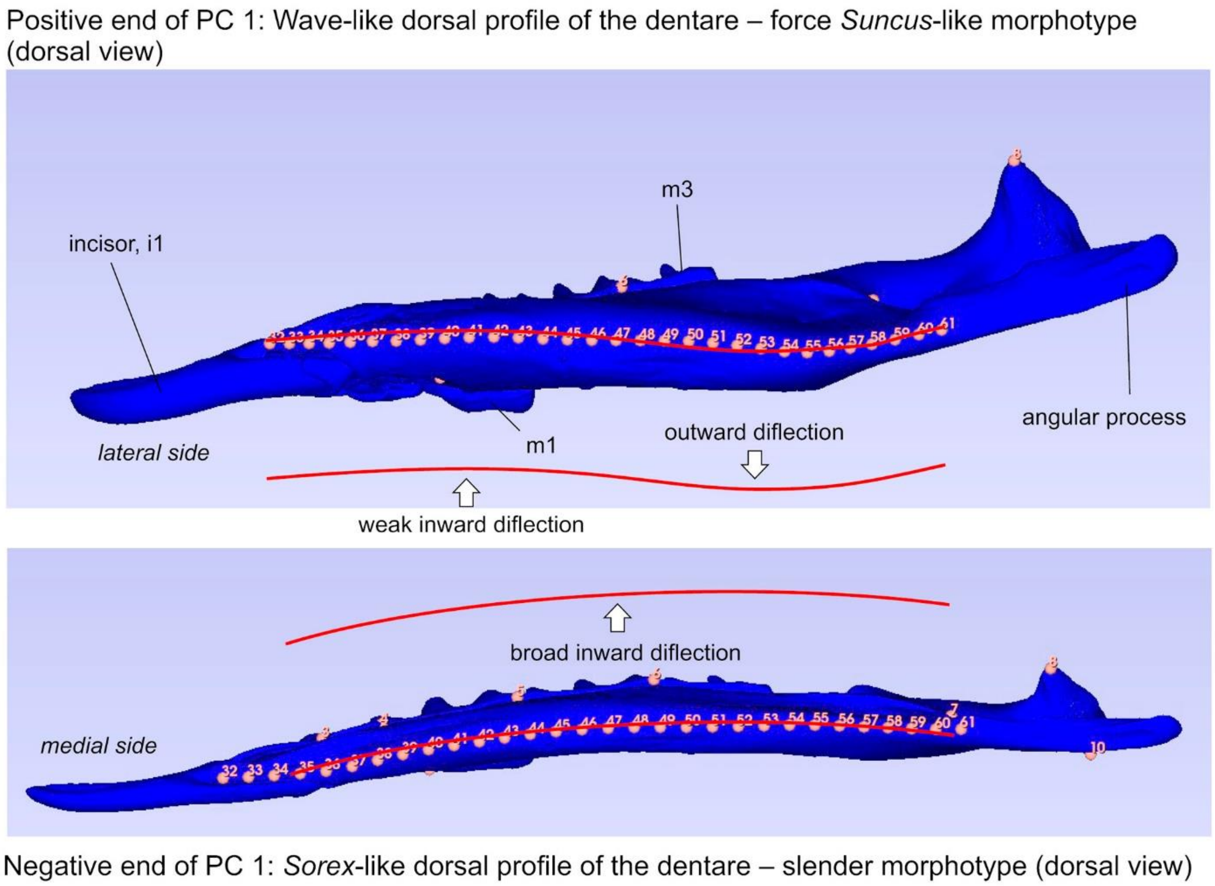

Figure S23. 3D plot of the mandibular shape morphospace along PC1–PC3 axes in two projections. (**a**) Projection is similar to 2D plot of the morphospace along of PC1–PC2 on Figure 8 (main text); (**b**) Projection is rotated by ca. 75 deg. counterclockwise. *Key*: **arm**, *C. armenica* (holotype, ZIN 45277); **arm-t**, *C. armenica* (paratype, ZIN 55321); **arm_972**, *C. armenica* (ZIN 77972); **arm_973**, *C. armenica* (ZIN 767973); **arm_976**, *C. armenica* (ZIN 77976); **ars**, *C. arispa* (holotype, NHMW 13284); **mrn**, *Suncus murinus* (ZIN 15885; outgroup); **mss**, *Sorex minutissimus* (ZIN 98582; outgroup); **szr**, *C. serezkyensis* (ZIN 77431).

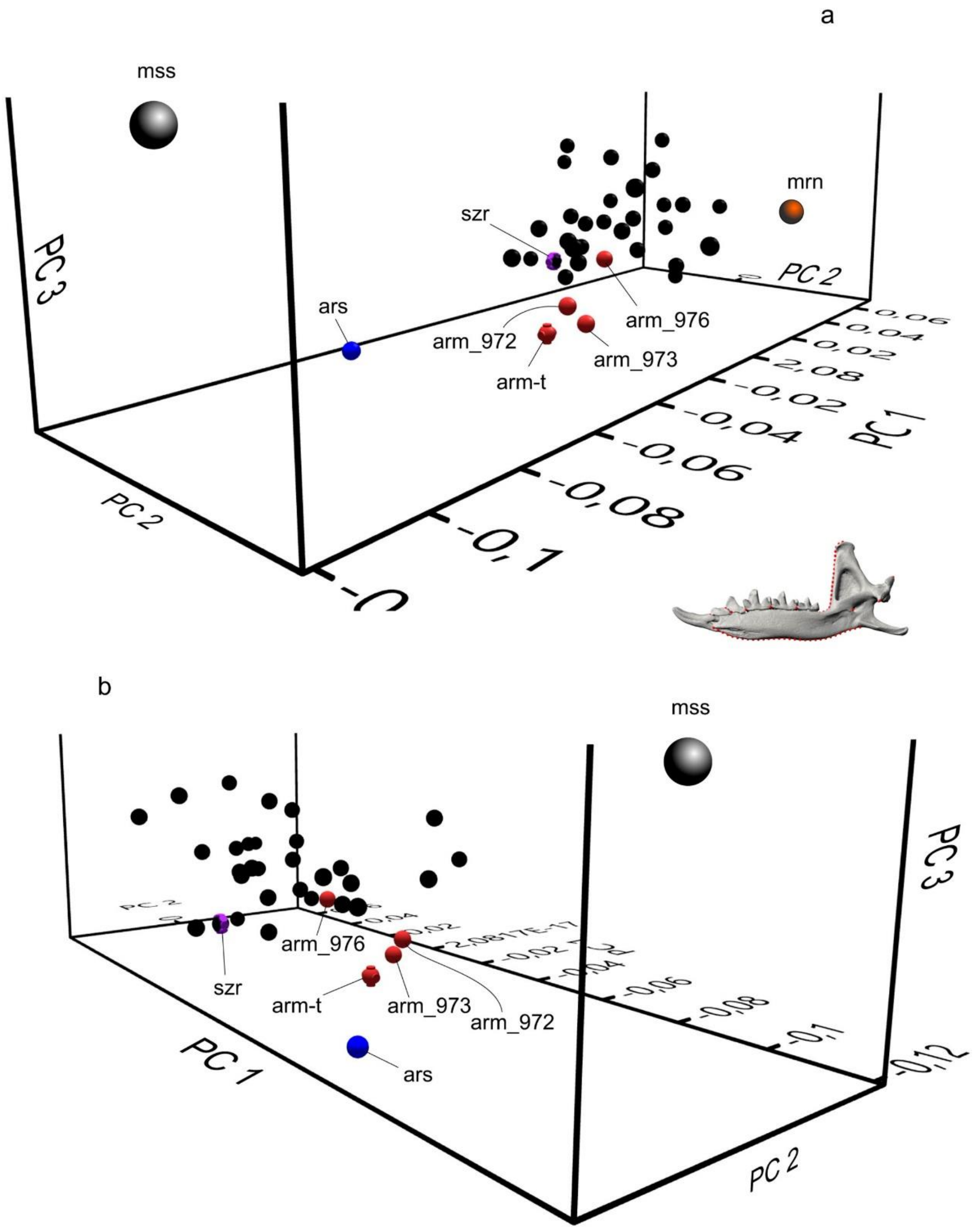

Figure S24. Result of the cluster analysis of Procrustes distances of the mandibular 3D shape data set of 36 specimens of shrews. Key see in the Table S1.

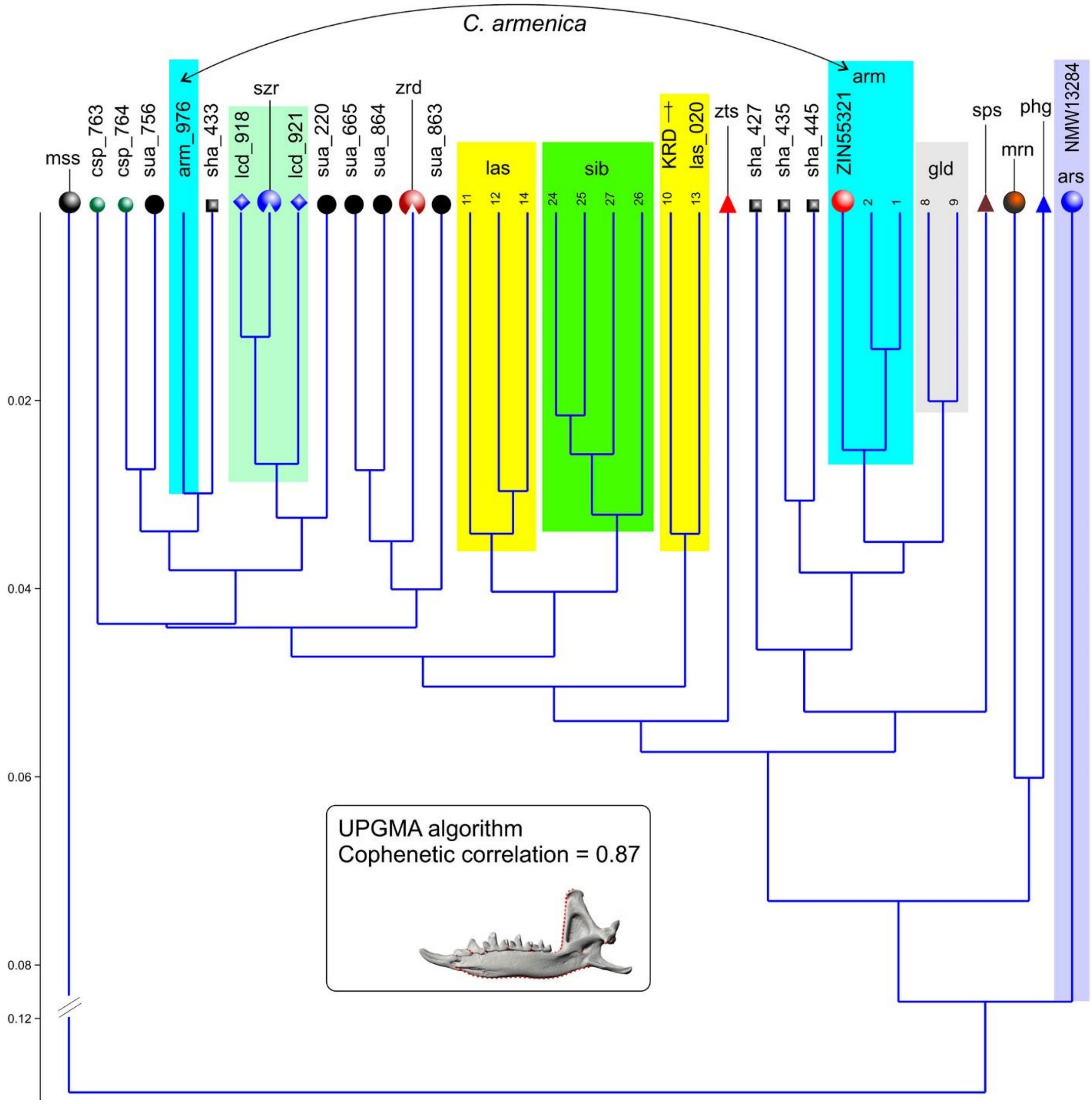

Figure S25. Result of the pairwise comparison of two 3D models of *C. arispa* (red) and *C. serezkyensis* (green) of the muzzle using 'Morpho' and 'rgl' packages of the R environment. For the correct transformation of 3D models during particular comparisons via R the left-side landmark data set was copied to the right side. Unscaled.

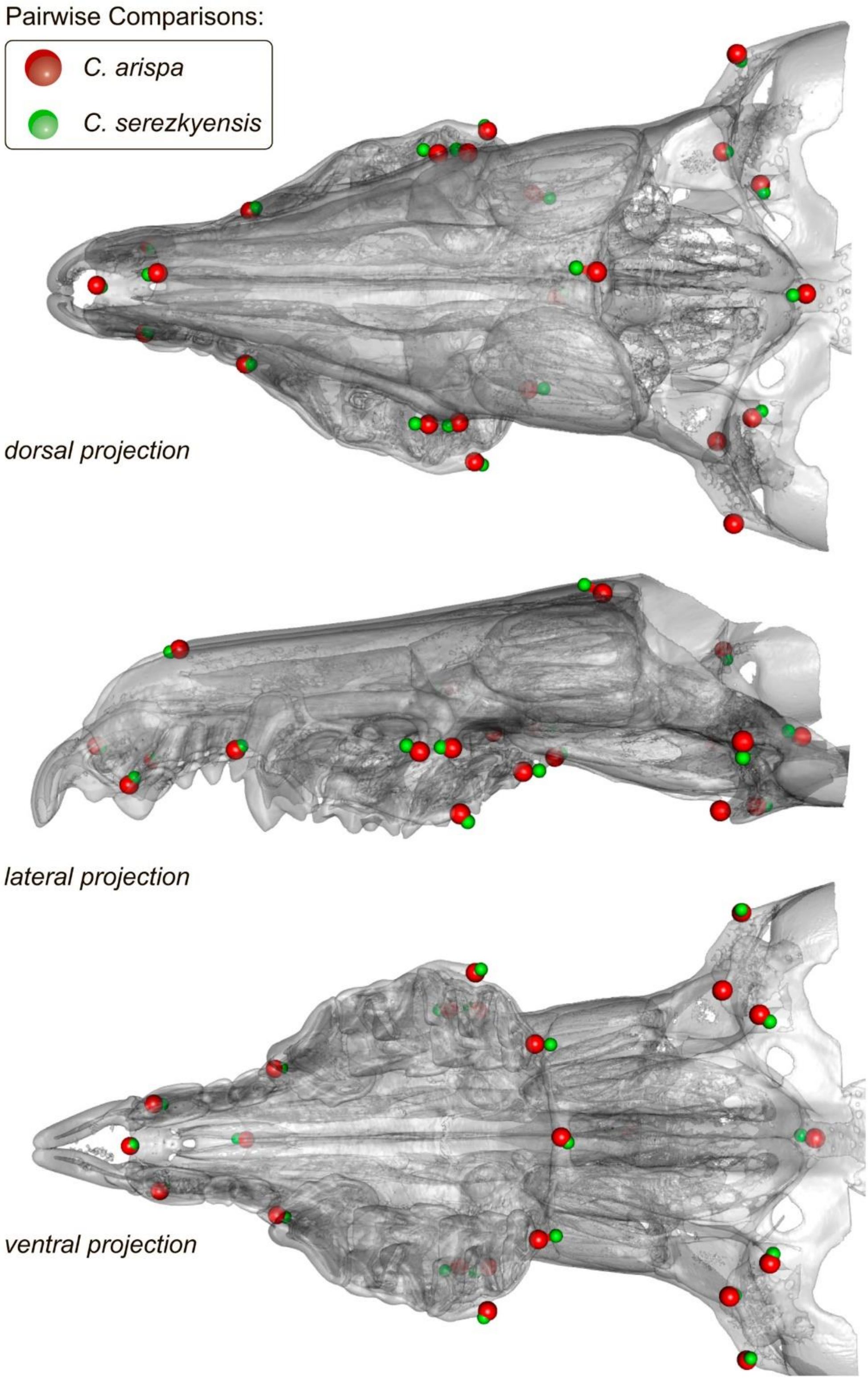

Figure S26. Result of the pairwise comparison of two 3D models of *C. serezkyensis* (green) and *C. armenica* (blue) of the muzzle using 'Morpho' and 'rgl' packages of the R environment. For the correct transformation of 3D models during particular comparisons via R the left-side landmark data set was copied to the right side. Unscaled.

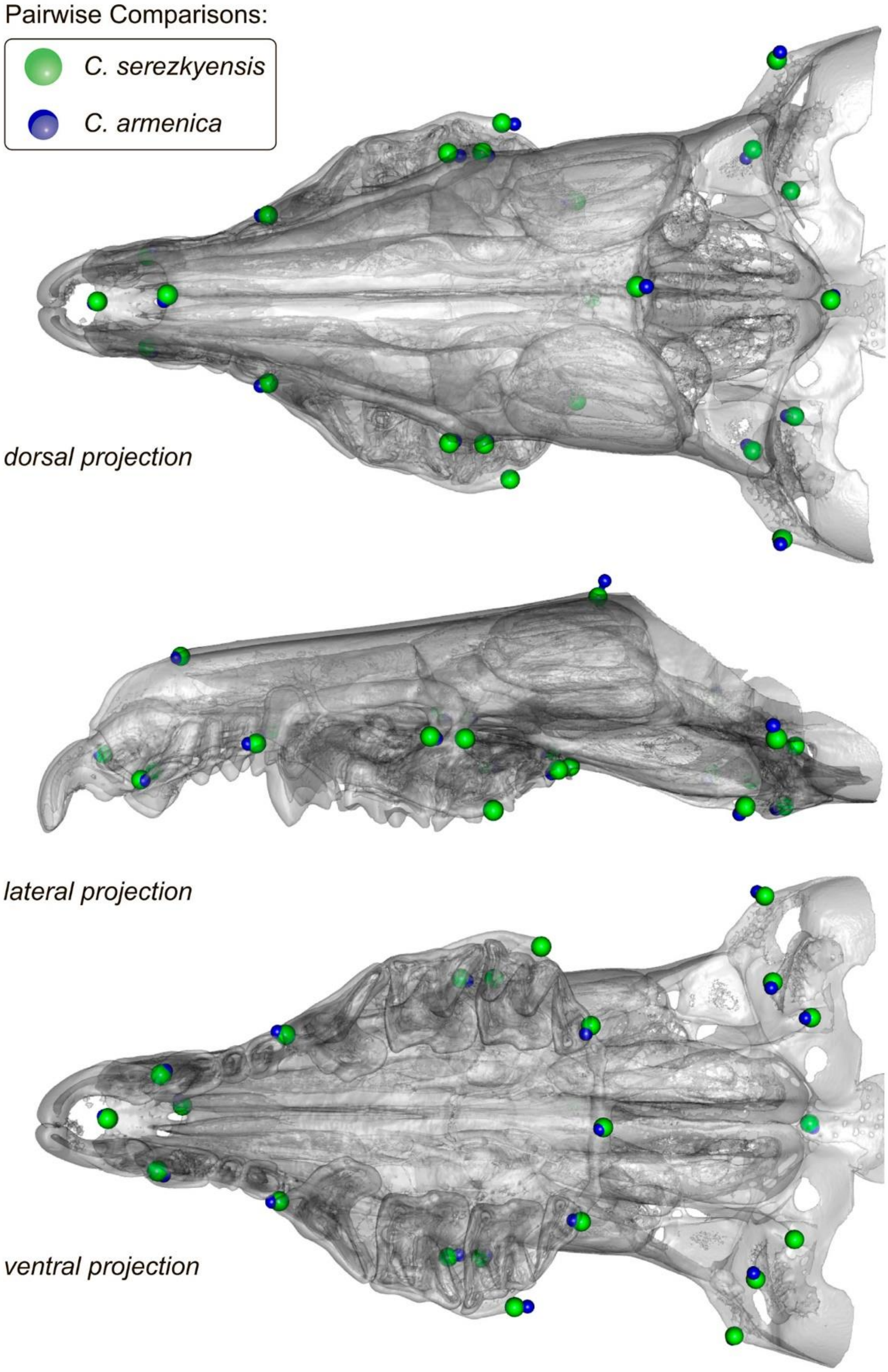

Figure S27. Result of the cluster analysis of Procrustes distances of the muzzle 3D shape data set of 35 specimens of shrews. Key see in the Table S1.

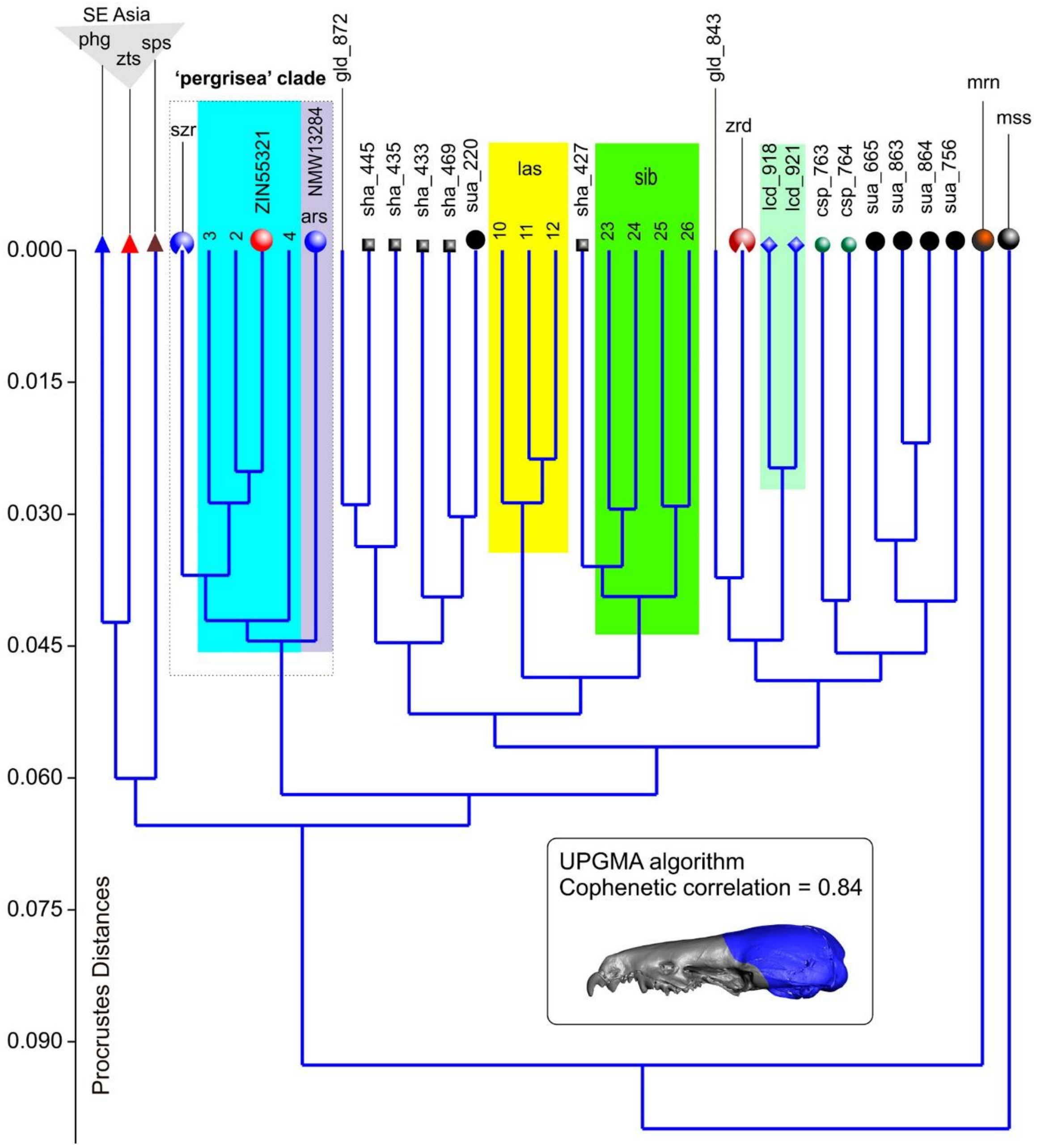

Figure S28. Screenshots of two 3D models of the skull of *Crocidura arispa* (holotype, NHMW 13284) under same-setting (a) and separate-setting (b) segmentation. (**a¹, b¹**) Models in lateral view; (**a², b²**) Halves of the muzzle model in occlusal view; (**a³**) Magnified part of the antemolar row with uncertain edges between A2 and A3 due to the dense matter imbalance; (**b³**) *ibid.*, edges are more clear.

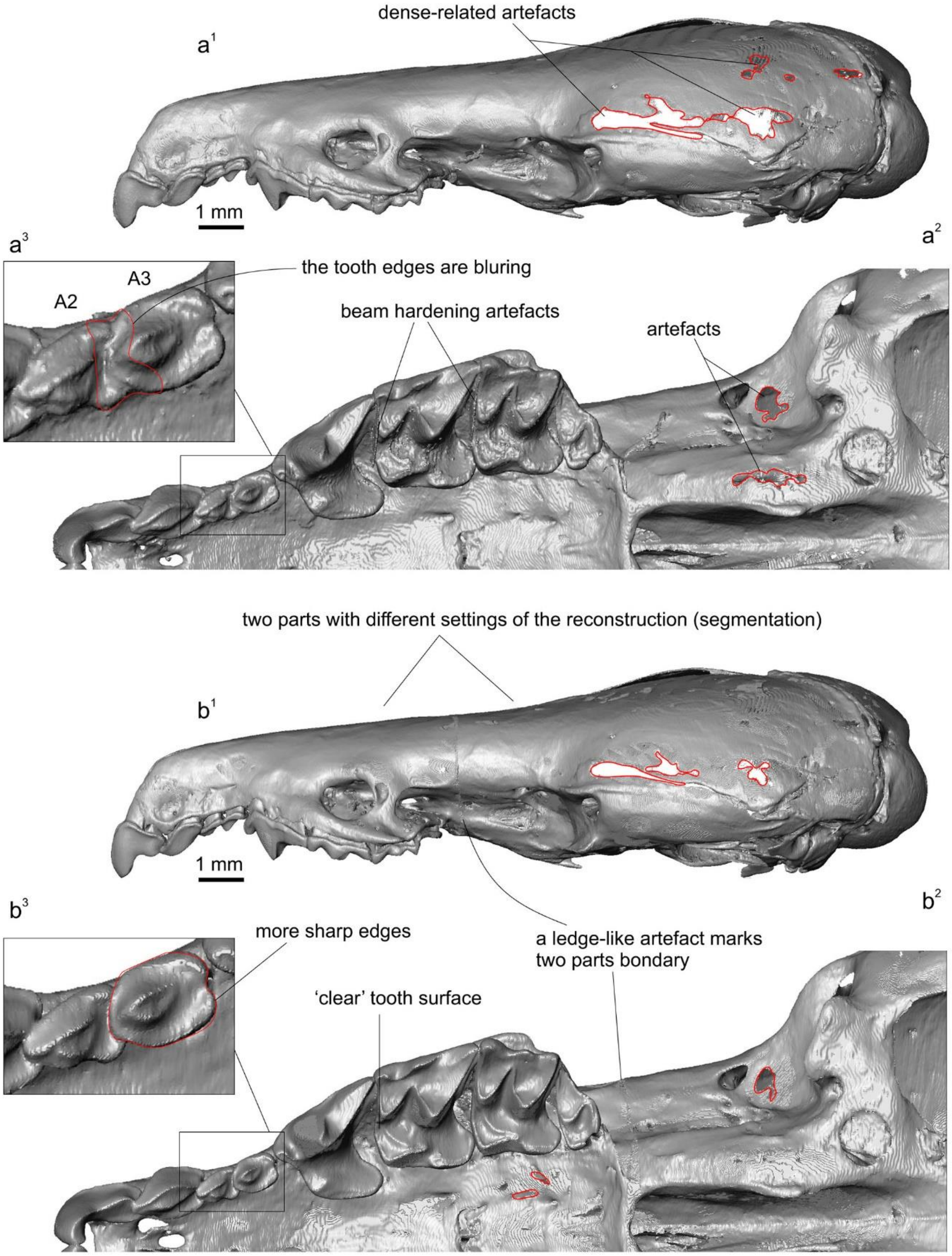

Supplement: Supplementary file 1 [file biology-13-00448-s001.zip › biology-3017363-supplementary.pdf]
